# 16S rRNA Gene-Based Metagenomic Analysis of Ozark Cave Bacteria

**DOI:** 10.3390/d9030031

**Published:** 2017-08-15

**Authors:** Cássia Oliveira, Lauren Gunderman, Cathryn A. Coles, Jason Lochmann, Megan Parks, Ethan Ballard, Galina Glazko, Yasir Rahmatallah, Alan J. Tackett, David J. Thomas

**Affiliations:** 1 Math and Science Division, Lyon College, 2300 Highland Road, Batesville, AR 72501, USA; 2 Department of Biomedical Informatics, University of Arkansas for Medical Sciences, Little Rock, AR 72205, USA; 3 Department of Biochemistry and Molecular Biology, University of Arkansas for Medical Sciences, Little Rock, AR 72205, USA

**Keywords:** bacteria, caves, chemoautotrophs, Illumina MiSeq Sequencing, 16S rRNA gene

## Abstract

The microbial diversity within cave ecosystems is largely unknown. Ozark caves maintain a year-round stable temperature (12–14 °C), but most parts of the caves experience complete darkness. The lack of sunlight and geological isolation from surface-energy inputs generate nutrient-poor conditions that may limit species diversity in such environments. Although microorganisms play a crucial role in sustaining life on Earth and impacting human health, little is known about their diversity, ecology, and evolution in community structures. We used five Ozark region caves as test sites for exploring bacterial diversity and monitoring long-term biodiversity. Illumina MiSeq sequencing of five cave soil samples and a control sample revealed a total of 49 bacterial phyla, with seven major phyla: Proteobacteria, Acidobacteria, Actinobacteria, Firmicutes, Chloroflexi, Bacteroidetes, and Nitrospirae. Variation in bacterial composition was observed among the five caves studied. Sandtown Cave had the lowest richness and most divergent community composition. 16S rRNA gene-based metagenomic analysis of cave-dwelling microbial communities in the Ozark caves revealed that species abundance and diversity are vast and included ecologically, agriculturally, and economically relevant taxa.

## 1. Introduction

Caves, caverns and other karst formations represent unique ecosystems that are relatively unexplored due to their subsurface location. Potential challenges include darkness, difficult access, and potential hazards such as slippery surfaces, loose rocks, and deep pits [1–4]. In caves, the lack of sunlight precludes photosynthesis except at cave openings where photosynthesis occurs at low levels. Reduced light intensity and illumination period select for organisms that can survive in reduced-light habitats, e.g., bryophytes, epilithic cyanobacteria, and algae. Most cave ecosystems are heterotrophic and depend upon organic materials that fall in through cave openings, carried by water, or deposited by cave animals that travel to the surface.

Typically, cave biologists focus on the macro-biological communities of caves while ignoring microbial life [4]. Therefore, the microbial diversity within the soils, streams and ponds in caves is largely unknown. While the lack of sunlight prevents the cavern from being an ideal habitat for most light-dependent macro-organisms, studies have shown that microbial life is diverse, including prokaryotes such as Proteobacteria, Actinobacteria, Acidobacteria, and Firmicutes and eukaryotes like yeasts and saprophytic fungi [2,5,6]. Subterranean microbes tend to have a low metabolic rate, such that maintenance is favored over growth [7]. A number of cave-dwelling microbial species are autotrophic and use chemosynthesis to drive biosynthetic reactions [8,9]. Furthermore, cave microorganisms have been implicated in mineral precipitation, which can lead to the formation of speleothems including pool fingers, moonmilk, stalactites, and stalagmites [2,10,11].

Although microbes play a key role in sustaining life on Earth [12], little is known about their diversity, ecology, and evolution. Despite all advances using classical microbiology techniques, our knowledge of microbial diversity is limited since only a small fraction of microbes can be isolated from natural environments [13,14]. Even when prokaryotes are amenable to the lab environment, species identification based on morphology and physiology can be challenging [15]. The number of bacterial phyla is estimated to be around one hundred [16,17]. Thirty percent of the phyla were characterized using culturing techniques, while the remaining seventy percent were identified using 16S rRNA [16]. Interestingly, none of the new species identified with 16S rRNA were found in cultures. The lack of a global and unified system to classify environmental 16S rRNA gene sequences has resulted in highly variable estimates of the total number of bacterial and archaeal species, ranging from 100,000 to over 1,000,000 microbial species per gram of soil [17–19]. In spite of this variation on the estimates, surface soil microbes display a significant stable community structure, in which nine major bacterial phyla are dominant: Proteobacteria, Actinobacteria, Acidobacteria, Chloroflexi, Bacteroidetes, Firmicutes, Planctomycetes, Verrucomicrobia and Gemmatimonadetes [20].

Five caves in north-central Arkansas were the subjects of this study: Cave Point Cave (Stone County), Bell Cave, Coon Creek Cave, Meacham Cave, and Sandtown Cave (Independence County). Coon Creek and Sandtown Caves are sandstone caves; the other three are limestone caves. All of the caves are on private property, but unauthorized entry into the caves occurs with varying frequency. Particularly in the limestone caves, vandals have caused extensive damage by spray-paint graffiti and breakage and removal of speleothem formations.

All five caves are considered “typical Ozark Caves” [21] in terms of their size, origin and biodiversity. The length of each cave is on the order of 200–2000 m. The caves were formed by water erosion within sandstone or limestone. All caves except Sandtown were formed within the Pitkin Limestone/Batesville Sandstone formations (approximately 323–346 Ma) [22]. Sandtown Cave formed within the St. Peter Sandstone (445–458 Ma). Each cave contains on the order of 15 conspicuous animal species, including salamanders (mostly *Eurycea* spp.), bats (usually *Perimyotis lucifugus*), other small mammals, crickets, spiders, springtails and millipedes. Notably, we have never found more than three bats of any species in Meacham Cave and Coon Creek Cave at any time, while the other three caves support hibernating bat populations in the range of 50–150 individuals. A detailed bioinventory of Meacham Cave was published previously [23]; published bioinventories of the other caves are not currently available.

All of the caves have mean air temperatures of 13–14 °C with less than 1 °C diurnal or seasonal variation past the twilight zone. Relative humidity is near or at 100% past the twilight zones in all of the caves. Water moves through all of the caves via seepage from the surface. Bell Cave and Cave Point Cave contain intermittent streams that flow through the main passages. Meacham Cave contains a seasonal pool in the main chamber, and Coon Creek Cave contains at least two pools that may be permanent. Sandtown Cave contains no bodies of water larger than puddles.

The present study explores bacterial diversity in five Arkansas Ozark caves using an Illumina-based 16S rRNA gene approach. We hypothesize that there exists a vast biodiversity of cave-dwelling microbes in these caves that culturing techniques have failed to uncover. These microbial communities are likely dominated by heterotrophic bacteria, but would also include low light and/or light independent autotrophic bacteria. These results will shed light on the subterranean ecosystems on Earth and aid in better long-term monitoring of their microbial biodiversity.

## 2. Materials and Methods

### 2.1. Cave Description and Sample Collection

All cave exploration was done in accordance with National Speleological Society guidelines [24]. Cave soil samples were collected from the region of 10–50 m from the entrance in 15 mL sterile centrifuge tubes. Samples were transported back to the lab at room temperature and stored at 4 °C prior to DNA extraction. We also extracted total metagenomic DNA from a lawn soil sample on the Lyon College campus for the comparison of bacterial composition and abundance. Previous studies have shown distinct differences between subterranean and surface environments (e.g., forests and agricultural fields) [25,26]. Therefore, we expected the lawn soil sample to show greater dissimilarity with other cave samples and function as an outgroup.

### 2.2. DNA Extraction and DNA Sequence Analysis

Total metagenomic DNA from one soil sample for each cave was extracted from freshly collected soil samples (within two months after collection) using PowerLyzer^®^ PowerSoil^®^ DNA isolation kit (MO BIO Laboratories, Inc., Carlsbad, CA, USA) according to the manufacturer’s instructions. DNA quantification was performed using a Qubit^™^ 3.0 Fluorometer (Life Technology Ltd., Paisley, UK). Presence of DNA was also verified by gel electrophoresing 10 μL of total DNA on 2% agarose using SYBR^®^ safe DNA gel stain (Invitrogen, Groningen, The Netherlands). The 260/280 ratio was measured using a Biophotometer (Eppendorf, Hamburg, Germany). DNA samples were stored at −20 °C until DNA sequencing was performed.

Samples were sequenced at the UAMS Sequencing Core Facility. V3 and V4 regions of the bacterial 16S rRNA gene were amplified using primers containing Illumina adapters following Illumina’s 16S Metagenomics Protocol (Part # 15044223 Rev. B). Briefly, Kapa Library Amplification Kit was used for PCR and products were cleaned using Beckman Coulter Agencourt AMPure XP Beads according to the 16S Metagenomics protocol. We used universal primers reported by Klindworth et al. [27]. Forward and reverse primer sequences are, respectively, 5′-TCGTCGGCAGCGTCAGATGTGTATAAGAG ACAGCCTACGGGNGGCWGCAG-3′ and 5′-GTCTCGTGGGCTCGGAGATGTGTATAAGAGAC AGGACTACHVGGGTATCTAATCC-3′, and create a single amplicon of approximately 460 bp. Concentrations were adjusted to 4 uM and prepared for loading on the Illumina Miseq according to Illumina’s 16S Metagenomics Protocol (Part # 15044223 Rev. B). Samples were pooled, denatured, and loaded on the Illumina Miseq at 8 pM and sequenced paired end (2 × 300) using a MiSeq^®^ Reagent Kit v3 (600 cycle) (Illumina, Inc., San Diego, CA, USA).

### 2.3. Bioinformatics and Statistical Analysis

16S rRNA metagenome sequencing data is available at the NCBI Sequence Read Archive (SRA) (http://www.ncbi.nlm.nih.gov/sra) under accession number PRJNA392322. Raw sequence data was processed in multiple steps using the Quantitative Insights into Microbial Ecology (QIIME) pipeline [28]. First, the 300 bp paired-end reads were joined using the *fastq-join* method with minimum allowed overlap of 120 bp and 15% maximum allowed difference within region of overlap. Second, reads with more than three consecutive base calls having Phred score <20 were truncated, reads with any ambiguous base call were discarded, and reads from different samples were tagged with sample identifiers and merged into a single FASTA file. Third, sequence reads were aligned against the core reference alignments of the Greengenes database (GG13_5; greengenes.lbl.gov) [29] using pyNAST [30] and operational taxonomic units (OTUs) were identified at the 97% DNA similarity level using UCLUST [31]. The counts of each OTU were normalized by the total number of aligned reads per sample. Sequences that failed the closed-reference alignment to the Greengenes database were aligned de novo and OTUs with <2 aligned sequences were discarded. While close-reference OTU picking detected 11,526 OTUs, 59,960 OTUs were picked de novo. We considered 64,395 OTUs associated with bacteria. Alpha and beta diversity metrics (Chao1 and UniFrac, respectively) were used to measure bacterial community composition [32–36]. We used hierarchical clustering and principal coordinate analysis (PCoA) of weighted Unifrac pairwise sample dissimilarity to show the similarity in OTU abundance profiles between samples. To test if the observed diversity in samples is associated with the total number of reads, we created a distance matrix using the pairwise difference in the number of aligned reads in samples and tested if the Pearson correlation coefficient of this matrix and the UniFrac (weighted or unweighted) distance matrix was significant.

## 3. Results

**Figure 1:**
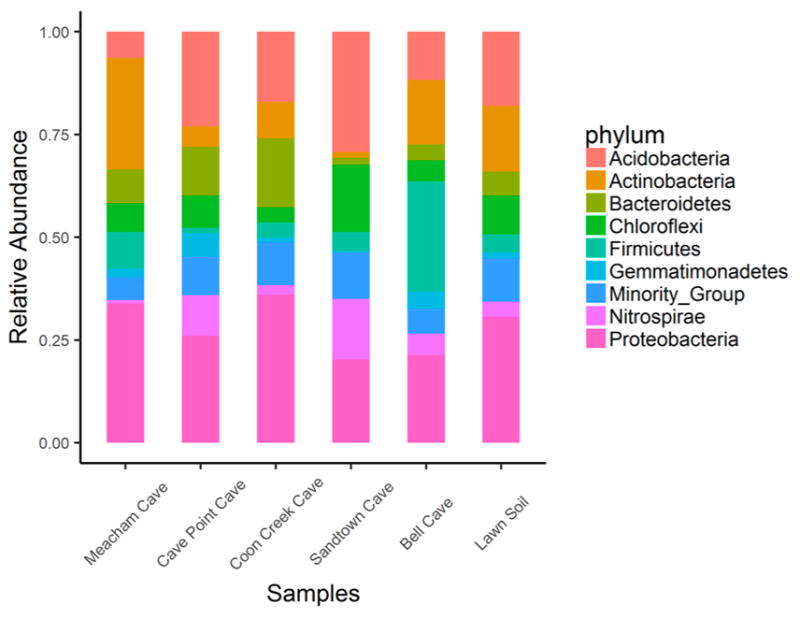
Barplot illustrating the diversity at the phylum level for cave and surface soil samples. Only the top eight phyla by average relative abundance across samples are shown and all other phyla were merged together into one group called Minority Group. Relative abundance of each bacterial phylum refers to the proportion of reads that aligned to OTUs associated with the phylum in each sample. Lawn soil was used as a control sample.

The total number of merged reads aligned successfully to each sample varied among the six samples. The sample from Bell Cave had the highest number of aligned reads (990,292), while Sandtown had the lowest number (193,424) (Table 1). Given the rarity of Archaea in our data (0.01–0.2% of reads), we decided to omit Archaea and focus on the bacterial communities. A total of 49 bacterial phyla were identified with seven major phyla (by average relative abundance across samples) represented among the five studied caves and lawn soil sample: Proteobacteria (average: 27.7%; range: 19.77–35.68%), Acidobacteria (17.3%; 6.33–28.55%), Actinobacteria (12.2%; 1.34–26.85%), Firmicutes (8.2%; 1.22–26.47%), Chloroflexi (8.1%; 3.64–15.88%), Bacteroidetes (8%; 1.78–16.62%), and Nitrospirae (6%, 0.9–14.4%). Figure 1 shows the relative abundance of the top eight bacterial phyla in all samples. All other phyla were merged together into one group called Minority group as indicated in the legend.

At the class level, Actinobacteria was present in all six locations. Alpha-, Gamma-, Beta-, and Delta-proteobacteria were also observed in all six locations, except for Sandtown Cave, where Delta-proteobacteria was absent, and Meacham Cave, which lacked Beta-proteobacteria. For Firmicutes, the class Clostridia was observed in all six locations, while Bacilli was present at Meacham Cave, Sandtown Cave, and Bell Cave. Sphingobacteriia (Phylum Bacteroidetes) was present at Meacham Cave, Coon Creek Cave, and Cave Point Cave. Nitrospira (Phylum Nitrospirae) was a top class in Sandtown Cave and Cave Point Cave, while Ktedonobacteria (Phylum Chloroflexi) and Acidobacteria (Phylum Acidobacteria) were restricted to Sandtown Cave (Supplemental Figure S1).

**Figure 2:**
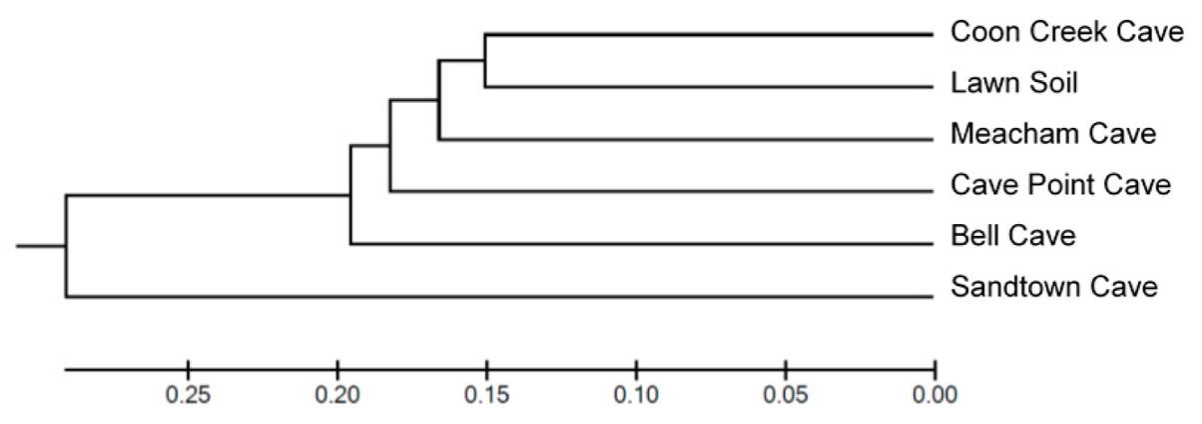
Dendrogram plot showing the similarity of soil microbial communities for five Ozark Caves and one lawn soil sample.

**Figure 3:**
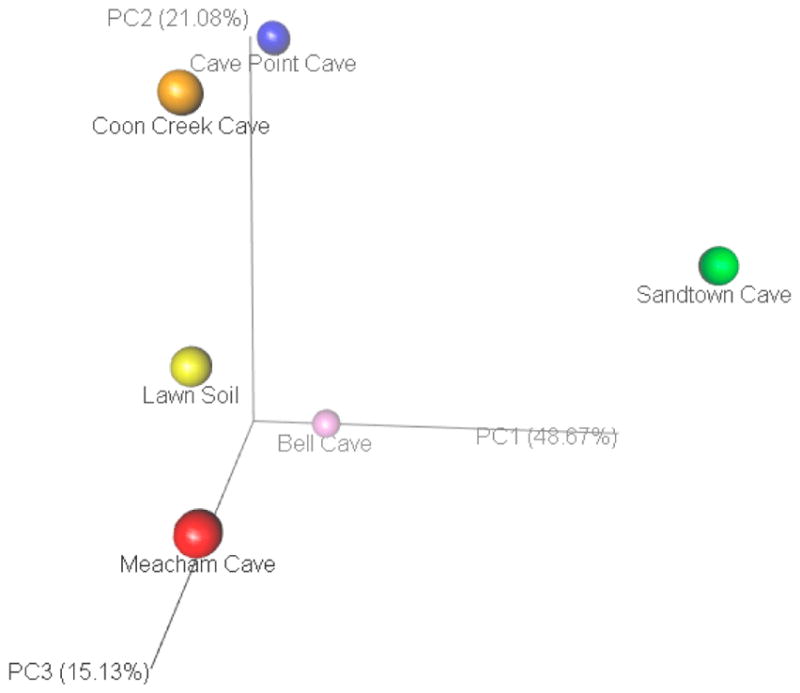
Principal Coordinate Analysis (PCoA) plot of weighted Unifrac distance matrix for five Ozark Caves and one lawn soil sample. First, three PCs explained 85% of the variation (shown in parentheses). Sandtown Cave had the most divergent microbial composition, whereas the other four caves and control sample had a more similar community composition.

Hierarchical clustering based on weighted Unifrac pairwise sample dissimilarity showed that Sandtown Cave is the most divergent sample among the five caves studied (Figure 2). Although the lawn soil was used as a control and was expected to be the outgroup, it clustered with Coon Creek Cave. Principal Coordinate Analysis (PCoA) of the weighted UniFrac matrix revealed a similar conclusion (Figure 3). Sandtown Cave had the largest average relative abundance of three phyla: Acidobacteria (28.55%), Chloroflexi (15.88%), and Nitrospirae (14.4%), but the lowest average relative abundance for Actinobacteria (1.34%) and Bacteriodetes (1.78%). It also contained several candidate phyla, e.g., GAL15, AD3, FCPU426, WPS-2 (Supplemental Table S1), that were over-represented.

**Figure 4:**
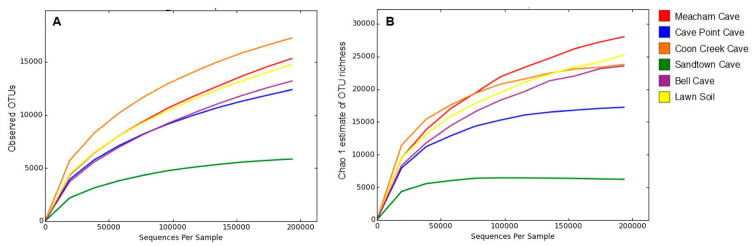
Rarefaction and Chao1 estimate of taxonomical richness for five Ozark Caves and a control sample (lawn soil). (**A**) rarefaction curves indicate the number of detected OTUs as more sequences are considered per sample; (**B**) Chao1 estimate indicates that Meacham Cave had the highest diversity, whereas Sandtown Cave had the lowest diversity.

The rarefaction curves showed that the full extent of the diversity was not sampled for four out of the five caves (Figure 4). Such results have been observed in other studies of bacterial diversity [5,37]. Chao1 metric revealed that Meacham Cave had the highest diversity, followed by the control sample (Lawn soil), Coon Creek Cave, Bell cave, Cave Point Cave, and Sandtown Cave (Figure 4). Since Sandtown yielded the minimum number of aligned merged reads among all samples (Table 1) and had the lowest taxonomic diversity (Figure 4), we checked if the UniFrac distance between the samples is associated with the number of aligned reads. Pearson correlation coefficient was low and non-significant (r = 0.23, *p* = 0.21 for weighted Unifrac, r = 0.12, *p* = 0.51 for unweighted Unifrac), indicating that the differences in microbial diversity were not associated with the number of total aligned reads per sample.

Ecologically relevant taxa identified in our study included a number of chemoautotrophs such as those involved in ammonium oxidation, e.g., *Bacillus* and *Nitrospira* [38], iron and manganese oxidation, e.g., *Pedimicrobium*, *Leptothrix*, and *Geobacter* [39–41], sulfur oxidation, e.g., *Paracoccus* and *Thiobacillus* [42] and methane oxidation, e.g., *Methylocaldum*, *Methylomonas*, and *Methylosarcina* [43] (Supplemental Table S1). Iron-, sulfur-, and manganese-oxidizing bacteria are implicated in cave dissolution processes, while species involved in constructive processes, like mineral precipitation, are linked with the formation of speleothems [2,10,11]. We also recovered taxa relevant to agriculture and human health, e.g., members of the Saccharopolyspora family have pesticide properties [44] and *Streptomyces* produce antibiotics and anti-cancer agents [45]. We observed low levels of human indicator bacteria: *Enterococcus spp*. and *Staphylococcus spp.* were present, but *Escherichia coli* was absent from all five caves.

## 4. Discussion

Our study represents the first culture-independent profiling of the microbial diversity in the Ozark caves. 16S rRNA gene-based metagenomic analysis identified 49 bacterial phyla, seven of which were over-represented in one or more caves: Proteobacteria, Acidobacteria, Actinobacteria, Firmicutes, Chloroflexi, Bacteroidetes, and Nitrospirae. All seven phyla, but Nitrospirae, are also dominant taxa in soil studies [20,46]. Our results are in agreement with other molecular microbial studies, showing that microbial communities in oligotrophic cave environments are phylogenetically diverse [2,5,10], but with a certain degree of stable microbial community structure.

Proteobacteria are widely distributed in terrestrial and marine environments, where they play key roles in biogeochemical cycles [47]. Proteobacteria is a highly diverse phylum composed of chemolithoautotrophs, heterotrophs, and mixotrophs and subdivided into five classes (Alpha-, Beta-, Gamma-, Delta- and Epsilon-proteobacteria) [36,37], all of which, except for the last class, were present in our samples (Supplemental Figure S1). Acidobacteria was the second highest represented phylum (17.3%) in our study and it is comprised of species distributed across a wide range of habitats [48]. Despite their ubiquitous distribution, little is known about Acidobacteria ecology and metabolism, mainly because of difficulties in cultivating these bacteria using classical techniques [49]. The Class Acidobacteria was only overrepresented in the Sandtown Cave.

The third most common phylum in our samples was Actinobacteria. This dominant bacterial phylum is composed of species with great ecological importance due to their roles in decomposition, humus formation, and nitrogen fixation in soil systems. Actinobacteria members are also economically and agriculturally relevant as a source of antibiotics [50] and pesticides [44]. Members of the Saccharopolyspora family produce metabolites named spinosyns, which have pesticide properties [44]. The Saccharopolyspora family was dominant in our caves, except for Coon Creek Cave. The *Streptomyces* genus (Streptomycetaceae family) are extremely relevant to human health as they can produce a wide range of secondary metabolites, including clinically useful antibiotics and anti-cancer agents [45]. *Streptomyces* were dominant in Meacham Cave.

Members of the fourth phylum, Firmicutes, are found in various environments and can be divided into anaerobes (e.g., Clostridia) and aerobes (e.g., Bacillus). The class Clostridia was over-represented in all six studied locations, while the class Bacillus was only observed at Meacham Cave, Sandtown Cave, and Bell Cave. Several *Bacillus* species can reduce nitrate to ammonia [38]. The fifth most common phylum, Chloroflexi, is best known to include photosynthetic bacteria [51]. In our study, we mostly observed members of the Ktedonobacteria class, which are aerobic heterotrophs. Bacteroidetes, the sixth phylum, include aerobes, anaerobes or facultative anaerobes depending on oxygen availability [46]. Finally, the Nitrospirae phylum is still largely unknown and include a number of nitrite-oxidizing bacteria such as those in the *Nitrospira* genus, which can transform ammonia (NH_3_) to nitrite (NO_2−_) [38]. The genus *Nitrospira* was dominant in all of our samples.

Several studies have shown that variation in microbial communities is higher across different environments. For instance, microbial communities in desert soils are more similar taxonomically, phylogenetically, and functionally than in non-desert soils [28]. Surface environments like forests and agricultural fields have a distinct composition from subterranean environments [25,26]. In subterranean soil samples Acidobacteria and Choroflexi seem to be more prevalent while Verrucomicrobia was absent [26]. Photosynthetic autotrophs like cyanobacteria are also commonly observed in the surface environment, but either absent or rarely found in aphotic cave environments. For instance, Hathaway et al. [5] observed very low levels of cyanobacteria in their study of lava caves. In the Ozark caves, we also observed only negligible numbers of cyanobacteria.

Microbial diversity can be determined by several factors including physical, chemical, and biological characteristics of ecosystems. For instance, pH has been shown to be an important abiotic predictor of soil bacterial diversity 54–55. Bacterial communities are more diverse in soils with near-neutral pH than acidic or basic soils. All of our soil cave samples had similar pH (6.5 ± 0.5) (Table 1). Although seven phyla were overrepresented in our samples, we also observed significant variation in bacterial composition in all five caves (Figures 1 and 3). Sandtown had the most divergent bacterial composition (Figure 2). Other important determinants of bacterial diversity include soil temperature, moisture, and nutrient availability (e.g., organic carbon) [12]. Sandtown Cave had the lowest community diversity, while Meacham Cave had the highest diversity (Figure 4). The differences in diversity between caves may be due in part to the caves’ origins and to the soils within. Sandtown and Coon Creek Caves are both sandstone caves. The cave soils are derived, at least in part, from the erosion of the parent materials. Sandtown Cave has very sandy soil, which does not hold nutrients well. Coon Creek Cave also has sandy soil, but it also has a higher clay content, which binds to nutrients, and potentially supports more and diverse microbes. The other three caves are limestone caves with soils comprised of mostly clay.

Low levels of human indicator bacteria, e.g., *Enterococcus*, *Staphylococcus*, and *E. coli*, observed in our study do not necessary imply that these caves are pristine, but rather that they have the potential to recover after several weeks following human contamination [52]. Thomas et al. [23] observed few vertebrates, high coliform bacterial load and vandalism in Meacham Cave in 2011, showing that human presence had significantly disrupted the cave ecosystem. In our present study of Meacham Cave, we also observed a low number of vertebrates, but we did not observe human indicator bacteria or recent vandalism, confirming that the cave ecosystem, including its microbial community, can revert back to a natural state.

## 5. Conclusions

Our results add evidence to a growing number of studies that have shown that the microbial diversity in caves goes well beyond samples being brought by water, air, or animals [53]. Instead, many of the cave microbes are genetically divergent from surface microbes and adapted to the aphotic and oligotrophic cave environment. Our samples were dominated by heterotrophic bacteria, but we also observed a number of autotrophic species, mainly chemoautotrophs. Overall, we were able to confirm that the biodiversity of cave-dwelling microbial communities in the Ozark caves is much vaster than we have been able to identify with classical microbiology techniques.

## Supplementary Material

Supplemental_Fig1

Supplemental_Table1

## Figures and Tables

**Table 1:** Abiotic characteristics of study caves. All five caves are located in north-central Arkansas, USA. Lawn soil from Lyon College, Batesville, AR, USA was used as a control sample. All soil samples were collected and DNA extracted in the Summer of 2015. 16S metagenomics sequencing statistics is shown on the last four columns.

Location	Cave Entrance Elevation (m)	Cave Temp. (°C)	Cave pH	Total Reads	Reads Passing Quality Filtering	% Reads Passing Quality Filtering	Aligned Merged Reads per Sample
Bell Cave	102	13.5 ± 0.5	6.5 ± 0.5	7,325,566	6,394,842	87.3%	990,292
Cave Point Cave	315	13.5 ± 0.5	6.5 ± 0.5	2,432,496	2,120,460	87.2%	292,934
Coon Creek Cave	159	13.5 ± 0.5	6.5 ± 0.5	2,592,318	2,225,312	85.8%	350,957
Meacham Cave	161	13.5 ± 0.5	6.5 ± 0.5	7,492,255	6,438,011	85.9%	865,302
Sandtown Cave	158	13.5 ± 0.5	6.5 ± 0.5	3,082,676	2,682,596	87.0%	193,424
Lawn Soil	NA	NA	NA	8,208,663	6,959,192	84.8%	918,247
