# Supplementary material for: 16S rRNA Gene-Based Metagenomic Analysis of Ozark Cave Bacteria
Source: Diversity (Basel). Author manuscript; Available in PMC 2018 Mar 16. (PMC5856467; doi:10.3390/d9030031)
Supplement: Supplemental_Fig1 [file NIHMS943117-supplement-Supplemental_Fig1.pdf]

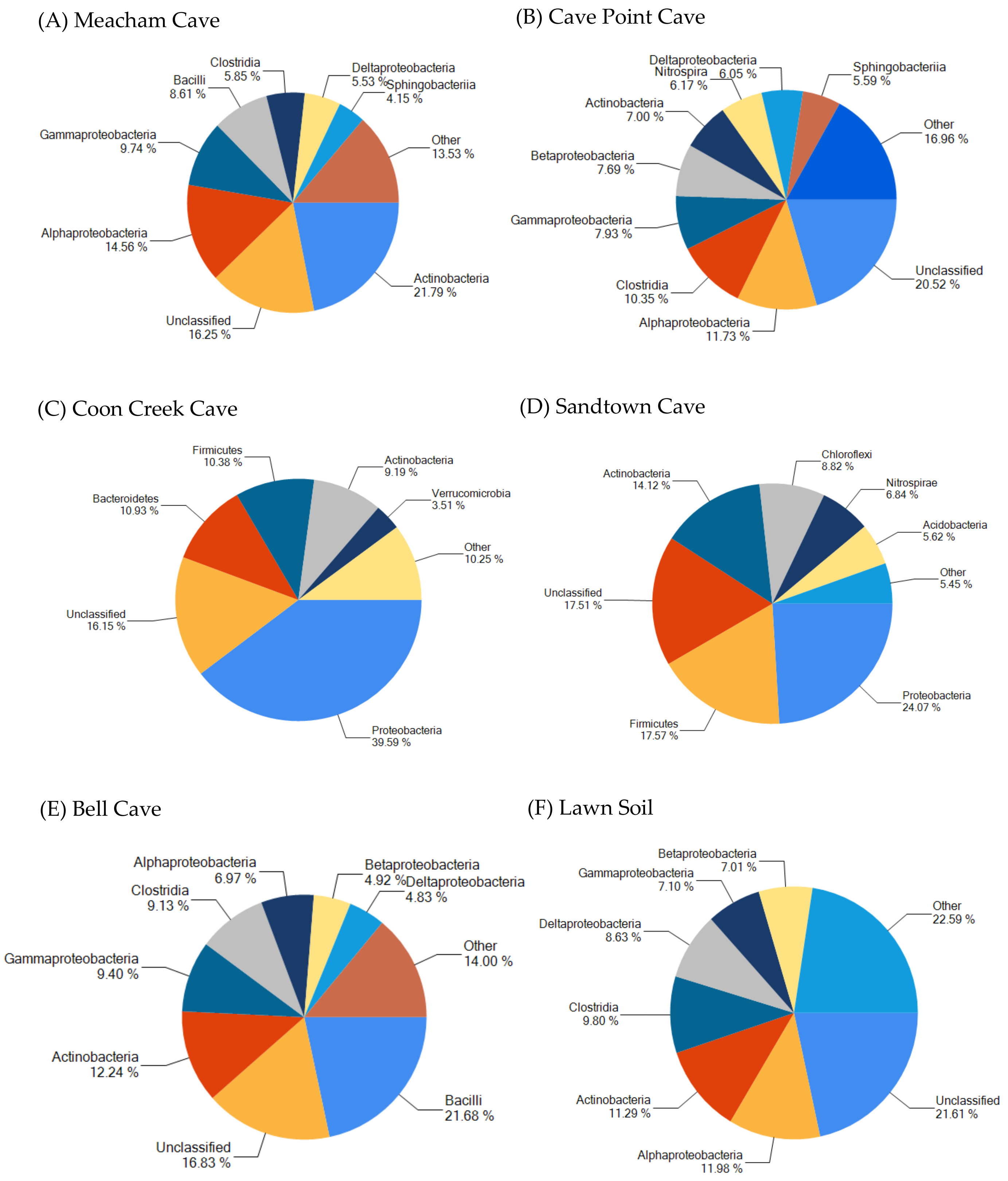


Supplemental Figure 1. Pie chart illustrating the diversity at the Class level for cave and surface soil samples. Number of Classes ranged from 60 to 67. The “Other" category in this pie chart is the sum of all classifications with less than 3.5% abundance.
