# Supplementary material for: 16S rRNA Gene-Based Metagenomic Analysis of Ozark Cave Bacteria
Source: Diversity (Basel). Author manuscript; Available in PMC 2018 Mar 16. (PMC5856467; doi:10.3390/d9030031)
Supplement: Supplemental_Table1 [file NIHMS943117-supplement-Supplemental_Table1.pdf]

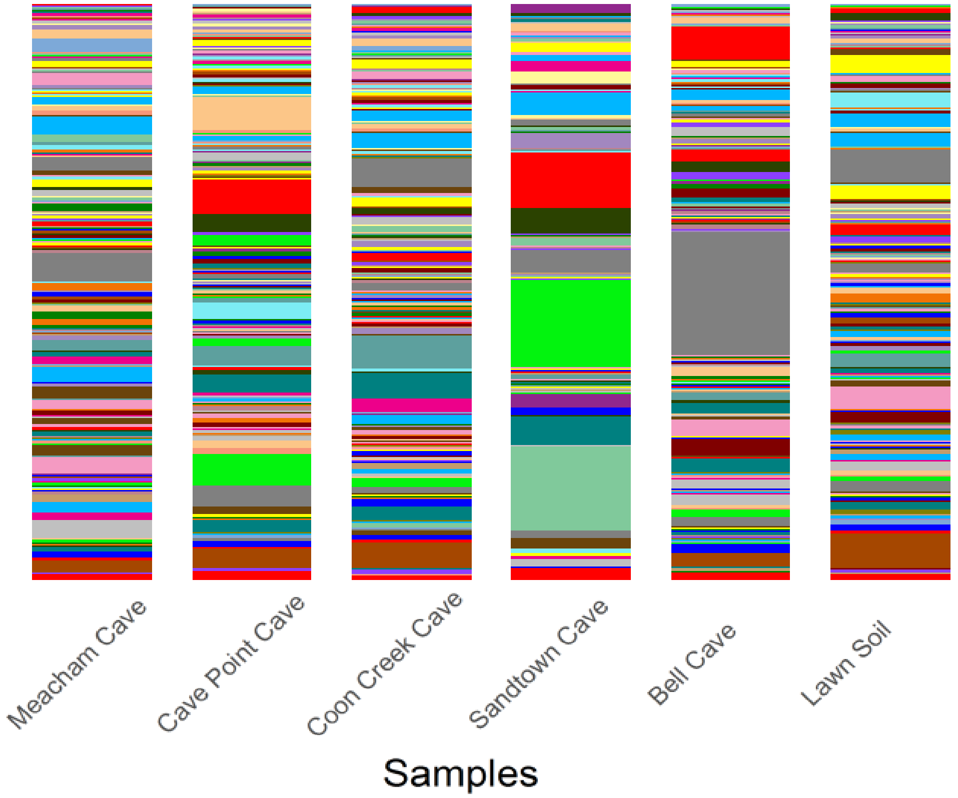

Legend	Taxonomy	Total	MGPLP13	MGPLP14	MGPLP17	MGPLP18	MGPLP20	MGPLP21
		%	%	%	%	%	%	%
	Unassigned;Other;Other;Other;Other;Other	1.3%	1.0%	1.4%	0.9%	2.2%	1.4%	1.1%
	k__Archaea;p__Crenarchaeota;c__MBGA;o__NRP-J;f__;g__	0.0%	0.0%	0.0%	0.0%	0.0%	0.0%	0.0%
	k__Archaea;p__Crenarchaeota;c__MCG;o__pGrfC26;f__;g__	0.0%	0.0%	0.0%	0.0%	0.0%	0.0%	0.0%
	k__Archaea;p__Crenarchaeota;c__Thaumarchaeota;o__AK31;f__;g__	0.0%	0.0%	0.0%	0.0%	0.0%	0.0%	0.0%
	k__Archaea;p__Crenarchaeota;c__Thaumarchaeota;o__Cenarchaeales;f__SAGMA-X;g__	0.0%	0.0%	0.0%	0.0%	0.0%	0.0%	0.0%
	k__Archaea;p__Crenarchaeota;c__Thaumarchaeota;o__Nitrososphaerales;f__Nitrososphaeraceae;g__Candidatus Nitrososphaera	0.0%	0.0%	0.0%	0.0%	0.0%	0.0%	0.0%
	k__Archaea;p__Crenarchaeota;c__Thermoprotei;o__;f__;g__	0.0%	0.0%	0.0%	0.0%	0.0%	0.0%	0.0%
	k__Archaea;p__Euryarchaeota;c__Methanobacteria;o__Methanobacteriales;f__Methanobacteriaceae;g__Methanobacterium	0.0%	0.0%	0.0%	0.0%	0.0%	0.0%	0.0%
	k__Archaea;p__Euryarchaeota;c__Methanomicrobia;o__Methanocellales;f__Methanocellaceae;g__Methanocella	0.0%	0.0%	0.0%	0.0%	0.0%	0.0%	0.0%
	k__Archaea;p__Euryarchaeota;c__Methanomicrobia;o__Methanosarcinales;f__Methanosarcinaceae;g__Methanosarcina	0.0%	0.0%	0.0%	0.0%	0.0%	0.0%	0.0%
	k__Archaea;p__Euryarchaeota;c__Methanomicrobia;o__YC-E6;f__;g__	0.0%	0.0%	0.0%	0.0%	0.0%	0.0%	0.0%
	k__Archaea;p__Euryarchaeota;c__Thermoplasmata;o__E2;f__;g__	0.0%	0.0%	0.0%	0.0%	0.0%	0.0%	0.0%
	k__Archaea;p__Euryarchaeota;c__Thermoplasmata;o__E2;f__[Methanomassiliicoccaceae];g__	0.0%	0.0%	0.0%	0.0%	0.2%	0.0%	0.0%
	k__Archaea;p__[Parvarchaeota];c__[Micrarchaea];o__[Micrarchaeles];f__;g__	0.0%	0.0%	0.0%	0.0%	0.0%	0.0%	0.0%
	k__Archaea;p__[Parvarchaeota];c__[Parvarchaea];o__WCHD3-30;f__;g__	0.0%	0.0%	0.0%	0.0%	0.0%	0.0%	0.0%
	k__Archaea;p__[Parvarchaeota];c__[Parvarchaea];o__YLA114;f__;g__	0.0%	0.0%	0.0%	0.0%	0.0%	0.0%	0.0%
	k__Bacteria;Other;Other;Other;Other;Other	0.0%	0.0%	0.0%	0.0%	0.0%	0.0%	0.0%
	k__Bacteria;p__;c__;o__;f__;g__	0.0%	0.0%	0.0%	0.0%	0.0%	0.0%	0.0%
	k__Bacteria;p__AD3;c__ABS-6;o__;f__;g__	0.2%	0.0%	0.0%	0.1%	1.3%	0.0%	0.0%
	k__Bacteria;p__AD3;c__JG37-AG-4;o__;f__;g__	0.1%	0.0%	0.0%	0.0%	0.4%	0.0%	0.0%
	k__Bacteria;p__Acidobacteria;Other;Other;Other;Other	0.0%	0.0%	0.0%	0.0%	0.0%	0.0%	0.0%
	k__Bacteria;p__Acidobacteria;c__;o__;f__;g__	0.1%	0.0%	0.2%	0.1%	0.0%	0.5%	0.1%
	k__Bacteria;p__Acidobacteria;c__AT-s54;o__;f__;g__	0.0%	0.0%	0.0%	0.0%	0.0%	0.0%	0.0%
	k__Bacteria;p__Acidobacteria;c__Acidobacteria-5;o__;f__;g__	0.4%	0.2%	0.3%	0.8%	0.0%	0.2%	0.7%
	k__Bacteria;p__Acidobacteria;c__Acidobacteria-6;Other;Other;Other	0.0%	0.0%	0.0%	0.0%	0.0%	0.0%	0.0%
	k__Bacteria;p__Acidobacteria;c__Acidobacteria-6;o__BPC015;f__;g__	0.0%	0.0%	0.0%	0.0%	0.0%	0.0%	0.0%
	k__Bacteria;p__Acidobacteria;c__Acidobacteria-6;o__CCU21;f__;g__	0.1%	0.0%	0.1%	0.1%	0.0%	0.1%	0.1%
	k__Bacteria;p__Acidobacteria;c__Acidobacteria-6;o__iii1-15;Other;Other	0.0%	0.0%	0.0%	0.0%	0.0%	0.0%	0.0%
	k__Bacteria;p__Acidobacteria;c__Acidobacteria-6;o__iii1-15;f__;g__	3.1%	2.2%	3.3%	4.5%	0.1%	2.2%	5.9%
	k__Bacteria;p__Acidobacteria;c__Acidobacteria-6;o__iii1-15;f__RB40;g__	0.3%	0.4%	0.4%	0.5%	0.0%	0.1%	0.6%
	k__Bacteria;p__Acidobacteria;c__Acidobacteria-6;o__iii1-15;f__mb2424;g__	0.9%	1.0%	1.1%	0.7%	0.0%	1.5%	1.0%

	k__Bacteria;p__Acidobacteria;c__Acidobacteriia;o__Acidobacteriales;Other;Other	0.0%	0.0%	0.0%	0.0%	0.0%	0.0%	0.0%
	k__Bacteria;p__Acidobacteria;c__Acidobacteriia;o__Acidobacteriales;f__;g__	0.0%	0.0%	0.0%	0.0%	0.0%	0.0%	0.0%
	k__Bacteria;p__Acidobacteria;c__Acidobacteriia;o__Acidobacteriales;f__Acidobacteriaceae;Other	0.0%	0.0%	0.0%	0.0%	0.0%	0.0%	0.0%
	k__Bacteria;p__Acidobacteria;c__Acidobacteriia;o__Acidobacteriales;f__Acidobacteriaceae;g__	0.1%	0.0%	0.0%	0.1%	0.6%	0.0%	0.0%
	k__Bacteria;p__Acidobacteria;c__Acidobacteriia;o__Acidobacteriales;f__Acidobacteriaceae;g__Edaphobacter	0.1%	0.0%	0.0%	0.0%	0.7%	0.0%	0.0%
	k__Bacteria;p__Acidobacteria;c__Acidobacteriia;o__Acidobacteriales;f__Acidobacteriaceae;g__Terriglobus	0.0%	0.0%	0.0%	0.0%	0.0%	0.0%	0.0%
	k__Bacteria;p__Acidobacteria;c__Acidobacteriia;o__Acidobacteriales;f__Koribacteraceae;Other	0.0%	0.0%	0.0%	0.0%	0.0%	0.0%	0.0%
	k__Bacteria;p__Acidobacteria;c__Acidobacteriia;o__Acidobacteriales;f__Koribacteraceae;g__	0.5%	0.0%	0.1%	0.7%	1.9%	0.1%	0.0%
	k__Bacteria;p__Acidobacteria;c__Acidobacteriia;o__Acidobacteriales;f__Koribacteraceae;g__Candidatus Koribacter	0.4%	0.0%	0.4%	0.4%	1.3%	0.0%	0.1%
	k__Bacteria;p__Acidobacteria;c__BPC102;o__;f__;g__	0.0%	0.0%	0.0%	0.0%	0.0%	0.0%	0.0%
	k__Bacteria;p__Acidobacteria;c__BPC102;o__B110;f__;g__	0.0%	0.0%	0.0%	0.0%	0.0%	0.0%	0.0%
	k__Bacteria;p__Acidobacteria;c__BPC102;o__MVS-40;f__;g__	0.3%	0.0%	0.4%	0.0%	0.0%	0.3%	0.8%
	k__Bacteria;p__Acidobacteria;c__DA052;o__E29;f__;g__	0.0%	0.0%	0.0%	0.0%	0.0%	0.0%	0.0%
	k__Bacteria;p__Acidobacteria;c__DA052;o__Ellin6513;f__;g__	2.6%	0.0%	0.0%	0.9%	14.5%	0.0%	0.0%
	k__Bacteria;p__Acidobacteria;c__EC1113;o__;f__;g__	0.0%	0.0%	0.1%	0.0%	0.0%	0.0%	0.0%
	k__Bacteria;p__Acidobacteria;c__GAL08;o__;f__;g__	0.0%	0.0%	0.0%	0.0%	0.0%	0.0%	0.0%
	k__Bacteria;p__Acidobacteria;c__Holophagae;o__Holophagales;f__Holophagaceae;g__	0.0%	0.0%	0.0%	0.0%	0.2%	0.0%	0.0%
	k__Bacteria;p__Acidobacteria;c__Holophagae;o__Holophagales;f__Holophagaceae;g__Geothrix	0.0%	0.0%	0.0%	0.0%	0.0%	0.0%	0.0%
	k__Bacteria;p__Acidobacteria;c__PAUC37f;o__;f__;g__	0.2%	0.0%	0.2%	0.2%	0.0%	0.2%	0.7%
	k__Bacteria;p__Acidobacteria;c__RB25;o__;f__;g__	0.1%	0.0%	0.1%	0.2%	0.0%	0.1%	0.2%
	k__Bacteria;p__Acidobacteria;c__S035;o__;f__;g__	0.3%	0.0%	0.2%	0.2%	0.0%	0.3%	1.0%
	k__Bacteria;p__Acidobacteria;c__Solibacteres;o__JH-WHS99;f__;g__	0.1%	0.0%	0.1%	0.0%	0.2%	0.1%	0.0%
	k__Bacteria;p__Acidobacteria;c__Solibacteres;o__Solibacterales;Other;Other	0.0%	0.0%	0.0%	0.0%	0.0%	0.2%	0.0%
	k__Bacteria;p__Acidobacteria;c__Solibacteres;o__Solibacterales;f__;g__	1.9%	0.8%	1.9%	2.2%	4.8%	0.6%	1.3%
	k__Bacteria;p__Acidobacteria;c__Solibacteres;o__Solibacterales;f__AKIW659;g__	0.0%	0.0%	0.0%	0.0%	0.0%	0.0%	0.0%
	k__Bacteria;p__Acidobacteria;c__Solibacteres;o__Solibacterales;f__PAUC26f;g__	0.1%	0.1%	0.0%	0.1%	0.2%	0.1%	0.0%
	k__Bacteria;p__Acidobacteria;c__Solibacteres;o__Solibacterales;f__PK34;g__	0.0%	0.0%	0.0%	0.0%	0.0%	0.0%	0.0%
	k__Bacteria;p__Acidobacteria;c__Solibacteres;o__Solibacterales;f__Solibacteraceae;g__	0.0%	0.0%	0.0%	0.1%	0.0%	0.0%	0.0%
	k__Bacteria;p__Acidobacteria;c__Solibacteres;o__Solibacterales;f__Solibacteraceae;g__Candidatus Solibacter	0.6%	0.1%	0.1%	1.3%	1.5%	0.2%	0.6%
	k__Bacteria;p__Acidobacteria;c__Solibacteres;o__Solibacterales;f__[Bryobacteraceae];g__	0.1%	0.1%	0.3%	0.2%	0.0%	0.0%	0.0%
	k__Bacteria;p__Acidobacteria;c__Sva0725;o__Sva0725;f__;g__	0.2%	0.3%	0.1%	0.1%	0.0%	0.1%	0.3%
	k__Bacteria;p__Acidobacteria;c__TM1;o__;f__;g__	0.4%	0.0%	0.0%	0.0%	2.3%	0.0%	0.0%
	k__Bacteria;p__Acidobacteria;c__[Chloracidobacteria];o__;f__;g__	0.0%	0.0%	0.0%	0.0%	0.0%	0.0%	0.0%
	k__Bacteria;p__Acidobacteria;c__[Chloracidobacteria];o__11-24;f__;g__	0.2%	0.0%	0.5%	0.2%	0.0%	0.2%	0.3%
	k__Bacteria;p__Acidobacteria;c__[Chloracidobacteria];o__DS-100;f__;g__	0.0%	0.0%	0.0%	0.0%	0.0%	0.0%	0.1%
	k__Bacteria;p__Acidobacteria;c__[Chloracidobacteria];o__Ellin7246;f__;g__	0.0%	0.0%	0.0%	0.0%	0.0%	0.0%	0.0%
	k__Bacteria;p__Acidobacteria;c__[Chloracidobacteria];o__PK29;f__;g__	0.4%	0.1%	1.4%	0.2%	0.0%	0.4%	0.4%
	k__Bacteria;p__Acidobacteria;c__[Chloracidobacteria];o__RB41;f__;g__	1.4%	0.1%	3.5%	1.3%	0.0%	1.7%	1.7%
	k__Bacteria;p__Acidobacteria;c__[Chloracidobacteria];o__RB41;f__Ellin6075;g__	1.6%	0.5%	5.5%	1.4%	0.2%	1.3%	0.9%
	k__Bacteria;p__Acidobacteria;c__iii1-8;o__;f__;g__	0.0%	0.0%	0.0%	0.0%	0.0%	0.0%	0.0%
	k__Bacteria;p__Acidobacteria;c__iii1-8;o__32-20;f__;g__	0.2%	0.0%	0.9%	0.0%	0.0%	0.3%	0.2%
	k__Bacteria;p__Acidobacteria;c__iii1-8;o__DS-18;f__;g__	0.5%	0.2%	1.3%	0.2%	0.0%	0.5%	0.7%
	k__Bacteria;p__Acidobacteria;c__iii1-8;o__SJA-36;f__;g__	0.0%	0.0%	0.0%	0.0%	0.0%	0.0%	0.1%
	k__Bacteria;p__Actinobacteria;Other;Other;Other;Other	0.0%	0.0%	0.0%	0.0%	0.0%	0.0%	0.0%
	k__Bacteria;p__Actinobacteria;c__Acidimicrobiia;o__Acidimicrobiales;Other;Other	0.0%	0.0%	0.0%	0.0%	0.0%	0.0%	0.0%
	k__Bacteria;p__Actinobacteria;c__Acidimicrobiia;o__Acidimicrobiales;f__;g__	1.4%	3.2%	0.7%	0.7%	0.3%	1.8%	1.7%
	k__Bacteria;p__Actinobacteria;c__Acidimicrobiia;o__Acidimicrobiales;f__AKIW874;g__	0.3%	1.2%	0.0%	0.0%	0.0%	0.2%	0.2%
	k__Bacteria;p__Actinobacteria;c__Acidimicrobiia;o__Acidimicrobiales;f__C111;g__	0.7%	1.8%	0.2%	0.7%	0.0%	0.3%	1.1%
	k__Bacteria;p__Actinobacteria;c__Acidimicrobiia;o__Acidimicrobiales;f__EB1017;g__	0.6%	1.4%	0.2%	1.0%	0.1%	0.3%	0.8%
	k__Bacteria;p__Actinobacteria;c__Acidimicrobiia;o__Acidimicrobiales;f__Iamiaceae;Other	0.0%	0.0%	0.0%	0.0%	0.0%	0.0%	0.0%
	k__Bacteria;p__Actinobacteria;c__Acidimicrobiia;o__Acidimicrobiales;f__Iamiaceae;g__	0.0%	0.0%	0.0%	0.0%	0.0%	0.0%	0.0%
	k__Bacteria;p__Actinobacteria;c__Acidimicrobiia;o__Acidimicrobiales;f__Iamiaceae;g__Iamia	0.1%	0.4%	0.0%	0.0%	0.0%	0.0%	0.0%
	k__Bacteria;p__Actinobacteria;c__Acidimicrobiia;o__Acidimicrobiales;f__Microthrixaceae;g__	0.0%	0.0%	0.0%	0.0%	0.0%	0.0%	0.0%
	k__Bacteria;p__Actinobacteria;c__Acidimicrobiia;o__Acidimicrobiales;f__TK06;g__	0.0%	0.0%	0.0%	0.0%	0.0%	0.0%	0.0%
	k__Bacteria;p__Actinobacteria;c__Acidimicrobiia;o__Acidimicrobiales;f__koll13;g__	0.0%	0.0%	0.0%	0.0%	0.0%	0.0%	0.2%
	k__Bacteria;p__Actinobacteria;c__Acidimicrobiia;o__Acidimicrobiales;f__ntu14;g__	0.0%	0.0%	0.0%	0.0%	0.0%	0.0%	0.0%
	k__Bacteria;p__Actinobacteria;c__Actinobacteria;o__Actinomycetales;Other;Other	0.0%	0.1%	0.0%	0.1%	0.0%	0.0%	0.1%
	k__Bacteria;p__Actinobacteria;c__Actinobacteria;o__Actinomycetales;f__;g__	0.2%	0.3%	0.0%	0.3%	0.1%	0.1%	0.1%
	k__Bacteria;p__Actinobacteria;c__Actinobacteria;o__Actinomycetales;f__ACK-M1;g__	0.0%	0.0%	0.0%	0.0%	0.0%	0.0%	0.0%
	k__Bacteria;p__Actinobacteria;c__Actinobacteria;o__Actinomycetales;f__Actinomycetaceae;g__Actinomyces	0.0%	0.0%	0.0%	0.0%	0.0%	0.0%	0.0%
	k__Bacteria;p__Actinobacteria;c__Actinobacteria;o__Actinomycetales;f__Actinospicaceae;g__	0.0%	0.0%	0.0%	0.0%	0.0%	0.0%	0.0%
	k__Bacteria;p__Actinobacteria;c__Actinobacteria;o__Actinomycetales;f__Actinosynnemataceae;Other	0.0%	0.1%	0.0%	0.0%	0.0%	0.0%	0.0%
	k__Bacteria;p__Actinobacteria;c__Actinobacteria;o__Actinomycetales;f__Actinosynnemataceae;g__	0.0%	0.3%	0.0%	0.0%	0.0%	0.0%	0.0%
	k__Bacteria;p__Actinobacteria;c__Actinobacteria;o__Actinomycetales;f__Actinosynnemataceae;g__Kibdelosporangium	0.0%	0.0%	0.0%	0.0%	0.0%	0.0%	0.0%
	k__Bacteria;p__Actinobacteria;c__Actinobacteria;o__Actinomycetales;f__Actinosynnemataceae;g__Lentzea	0.0%	0.0%	0.0%	0.0%	0.0%	0.0%	0.0%
	k__Bacteria;p__Actinobacteria;c__Actinobacteria;o__Actinomycetales;f__Beutenbergiaceae;Other	0.0%	0.0%	0.0%	0.0%	0.0%	0.0%	0.0%
	k__Bacteria;p__Actinobacteria;c__Actinobacteria;o__Actinomycetales;f__Beutenbergiaceae;g__Salana	0.0%	0.0%	0.0%	0.0%	0.0%	0.0%	0.0%

k__Bacteria;p__Actinobacteria;c__Actinobacteria;o__Actinomycetales;f__Bogoriellaceae;g__Georgenia	0.0%	0.0%	0.0%	0.0%	0.0%	0.0%	0.0%
k__Bacteria;p__Actinobacteria;c__Actinobacteria;o__Actinomycetales;f__Brevibacteriaceae;g__Brevibacterium	0.0%	0.0%	0.0%	0.0%	0.0%	0.0%	0.0%
k__Bacteria;p__Actinobacteria;c__Actinobacteria;o__Actinomycetales;f__Cellulomonadaceae;Other	0.0%	0.0%	0.0%	0.0%	0.0%	0.0%	0.0%
k__Bacteria;p__Actinobacteria;c__Actinobacteria;o__Actinomycetales;f__Cellulomonadaceae;g__Cellulomonas	0.0%	0.0%	0.0%	0.0%	0.0%	0.0%	0.0%
k__Bacteria;p__Actinobacteria;c__Actinobacteria;o__Actinomycetales;f__Cellulomonadaceae;g__Demequina	0.0%	0.0%	0.0%	0.0%	0.0%	0.0%	0.0%
k__Bacteria;p__Actinobacteria;c__Actinobacteria;o__Actinomycetales;f__Corynebacteriaceae;g__Corynebacterium	0.0%	0.0%	0.0%	0.0%	0.0%	0.0%	0.0%
k__Bacteria;p__Actinobacteria;c__Actinobacteria;o__Actinomycetales;f__Cryptosporangiaceae;Other	0.0%	0.0%	0.0%	0.0%	0.0%	0.0%	0.0%
k__Bacteria;p__Actinobacteria;c__Actinobacteria;o__Actinomycetales;f__Cryptosporangiaceae;g__	0.0%	0.0%	0.0%	0.0%	0.0%	0.0%	0.0%
k__Bacteria;p__Actinobacteria;c__Actinobacteria;o__Actinomycetales;f__Dermabacteraceae;g__Brachybacterium	0.0%	0.0%	0.0%	0.0%	0.0%	0.0%	0.0%
k__Bacteria;p__Actinobacteria;c__Actinobacteria;o__Actinomycetales;f__Dermacoccaceae;g__Dermacoccus	0.0%	0.0%	0.0%	0.0%	0.0%	0.0%	0.0%
k__Bacteria;p__Actinobacteria;c__Actinobacteria;o__Actinomycetales;f__Dietziaceae;g__Dietzia	0.0%	0.0%	0.0%	0.0%	0.0%	0.0%	0.0%
k__Bacteria;p__Actinobacteria;c__Actinobacteria;o__Actinomycetales;f__Frankiaceae;Other	0.0%	0.0%	0.0%	0.0%	0.0%	0.0%	0.0%
k__Bacteria;p__Actinobacteria;c__Actinobacteria;o__Actinomycetales;f__Frankiaceae;g__	0.0%	0.0%	0.0%	0.0%	0.0%	0.0%	0.0%
k__Bacteria;p__Actinobacteria;c__Actinobacteria;o__Actinomycetales;f__Frankiaceae;g__Frankia	0.0%	0.0%	0.0%	0.0%	0.0%	0.0%	0.0%
k__Bacteria;p__Actinobacteria;c__Actinobacteria;o__Actinomycetales;f__Geodermatophilaceae;Other	0.0%	0.0%	0.0%	0.0%	0.0%	0.0%	0.0%
k__Bacteria;p__Actinobacteria;c__Actinobacteria;o__Actinomycetales;f__Geodermatophilaceae;g__	0.0%	0.0%	0.0%	0.0%	0.0%	0.0%	0.0%
k__Bacteria;p__Actinobacteria;c__Actinobacteria;o__Actinomycetales;f__Geodermatophilaceae;g__Geodermatophilus	0.0%	0.0%	0.0%	0.0%	0.0%	0.0%	0.0%
k__Bacteria;p__Actinobacteria;c__Actinobacteria;o__Actinomycetales;f__Geodermatophilaceae;g__Modestobacter	0.0%	0.0%	0.0%	0.0%	0.0%	0.0%	0.0%
k__Bacteria;p__Actinobacteria;c__Actinobacteria;o__Actinomycetales;f__Glycomycetaceae;g__Glycomyces	0.0%	0.0%	0.0%	0.0%	0.0%	0.0%	0.0%
k__Bacteria;p__Actinobacteria;c__Actinobacteria;o__Actinomycetales;f__Gordoniaceae;g__Gordonia	0.0%	0.0%	0.0%	0.0%	0.0%	0.0%	0.0%
k__Bacteria;p__Actinobacteria;c__Actinobacteria;o__Actinomycetales;f__Intrasporangiaceae;Other	0.1%	0.0%	0.0%	0.0%	0.0%	0.1%	0.2%
k__Bacteria;p__Actinobacteria;c__Actinobacteria;o__Actinomycetales;f__Intrasporangiaceae;g__	0.0%	0.1%	0.0%	0.0%	0.0%	0.0%	0.0%
k__Bacteria;p__Actinobacteria;c__Actinobacteria;o__Actinomycetales;f__Intrasporangiaceae;g__Knoellia	0.0%	0.0%	0.0%	0.0%	0.0%	0.0%	0.0%
k__Bacteria;p__Actinobacteria;c__Actinobacteria;o__Actinomycetales;f__Intrasporangiaceae;g__Phycicoccus	0.0%	0.0%	0.0%	0.0%	0.0%	0.0%	0.0%
k__Bacteria;p__Actinobacteria;c__Actinobacteria;o__Actinomycetales;f__Intrasporangiaceae;g__Terracoccus	0.0%	0.0%	0.0%	0.0%	0.0%	0.0%	0.0%
k__Bacteria;p__Actinobacteria;c__Actinobacteria;o__Actinomycetales;f__Kineosporiaceae;Other	0.0%	0.0%	0.0%	0.0%	0.0%	0.0%	0.0%
k__Bacteria;p__Actinobacteria;c__Actinobacteria;o__Actinomycetales;f__Kineosporiaceae;g__	0.0%	0.0%	0.0%	0.0%	0.0%	0.0%	0.0%
k__Bacteria;p__Actinobacteria;c__Actinobacteria;o__Actinomycetales;f__Microbacteriaceae;Other	0.0%	0.0%	0.0%	0.0%	0.0%	0.0%	0.0%
k__Bacteria;p__Actinobacteria;c__Actinobacteria;o__Actinomycetales;f__Microbacteriaceae;g__	0.0%	0.1%	0.0%	0.1%	0.0%	0.0%	0.0%
k__Bacteria;p__Actinobacteria;c__Actinobacteria;o__Actinomycetales;f__Microbacteriaceae;g__Agromyces	0.1%	0.4%	0.0%	0.0%	0.0%	0.0%	0.0%
k__Bacteria;p__Actinobacteria;c__Actinobacteria;o__Actinomycetales;f__Microbacteriaceae;g__Cryocola	0.0%	0.0%	0.0%	0.0%	0.0%	0.0%	0.0%
k__Bacteria;p__Actinobacteria;c__Actinobacteria;o__Actinomycetales;f__Microbacteriaceae;g__Leucobacter	0.0%	0.0%	0.0%	0.0%	0.0%	0.0%	0.0%
k__Bacteria;p__Actinobacteria;c__Actinobacteria;o__Actinomycetales;f__Microbacteriaceae;g__Microbacterium	0.0%	0.1%	0.0%	0.0%	0.0%	0.0%	0.0%
k__Bacteria;p__Actinobacteria;c__Actinobacteria;o__Actinomycetales;f__Microbacteriaceae;g__Mycetocola	0.0%	0.0%	0.0%	0.0%	0.0%	0.0%	0.0%
k__Bacteria;p__Actinobacteria;c__Actinobacteria;o__Actinomycetales;f__Microbacteriaceae;g__Salinibacterium	0.0%	0.0%	0.0%	0.0%	0.0%	0.0%	0.0%
k__Bacteria;p__Actinobacteria;c__Actinobacteria;o__Actinomycetales;f__Micrococcaceae;Other	0.0%	0.0%	0.0%	0.0%	0.0%	0.0%	0.0%
k__Bacteria;p__Actinobacteria;c__Actinobacteria;o__Actinomycetales;f__Micrococcaceae;g__	0.5%	0.1%	0.7%	0.3%	0.1%	1.6%	0.2%
k__Bacteria;p__Actinobacteria;c__Actinobacteria;o__Actinomycetales;f__Micrococcaceae;g__Arthrobacter	0.1%	0.0%	0.1%	0.1%	0.0%	0.1%	0.0%
k__Bacteria;p__Actinobacteria;c__Actinobacteria;o__Actinomycetales;f__Micrococcaceae;g__Microbispora	0.0%	0.0%	0.0%	0.0%	0.0%	0.0%	0.0%
k__Bacteria;p__Actinobacteria;c__Actinobacteria;o__Actinomycetales;f__Micrococcaceae;g__Micrococcus	0.0%	0.0%	0.0%	0.0%	0.0%	0.0%	0.0%
k__Bacteria;p__Actinobacteria;c__Actinobacteria;o__Actinomycetales;f__Micromonosporaceae;Other	0.0%	0.2%	0.0%	0.0%	0.0%	0.0%	0.0%
k__Bacteria;p__Actinobacteria;c__Actinobacteria;o__Actinomycetales;f__Micromonosporaceae;g__	0.1%	0.3%	0.0%	0.1%	0.0%	0.0%	0.1%
k__Bacteria;p__Actinobacteria;c__Actinobacteria;o__Actinomycetales;f__Micromonosporaceae;g__Actinoplanes	0.0%	0.0%	0.0%	0.0%	0.0%	0.0%	0.0%
k__Bacteria;p__Actinobacteria;c__Actinobacteria;o__Actinomycetales;f__Micromonosporaceae;g__Catellatospora	0.0%	0.1%	0.0%	0.0%	0.0%	0.0%	0.0%
k__Bacteria;p__Actinobacteria;c__Actinobacteria;o__Actinomycetales;f__Micromonosporaceae;g__Dactylosporangium	0.0%	0.0%	0.0%	0.0%	0.0%	0.0%	0.0%
k__Bacteria;p__Actinobacteria;c__Actinobacteria;o__Actinomycetales;f__Micromonosporaceae;g__Micromonospora	0.0%	0.0%	0.0%	0.0%	0.0%	0.0%	0.0%
k__Bacteria;p__Actinobacteria;c__Actinobacteria;o__Actinomycetales;f__Micromonosporaceae;g__Pilimelia	0.0%	0.1%	0.0%	0.1%	0.0%	0.0%	0.1%
k__Bacteria;p__Actinobacteria;c__Actinobacteria;o__Actinomycetales;f__Micromonosporaceae;g__Virgisporangium	0.0%	0.0%	0.0%	0.0%	0.0%	0.0%	0.0%
k__Bacteria;p__Actinobacteria;c__Actinobacteria;o__Actinomycetales;f__Mycobacteriaceae;g__Mycobacterium	0.3%	0.4%	0.0%	0.8%	0.1%	0.1%	0.2%
k__Bacteria;p__Actinobacteria;c__Actinobacteria;o__Actinomycetales;f__Nakamurellaceae;g__	0.0%	0.0%	0.0%	0.0%	0.0%	0.0%	0.0%
k__Bacteria;p__Actinobacteria;c__Actinobacteria;o__Actinomycetales;f__Nocardiaceae;Other	0.0%	0.0%	0.0%	0.0%	0.0%	0.0%	0.0%
k__Bacteria;p__Actinobacteria;c__Actinobacteria;o__Actinomycetales;f__Nocardiaceae;g__Nocardia	0.0%	0.1%	0.0%	0.1%	0.0%	0.0%	0.0%
k__Bacteria;p__Actinobacteria;c__Actinobacteria;o__Actinomycetales;f__Nocardiaceae;g__Rhodococcus	0.1%	0.1%	0.0%	0.2%	0.1%	0.0%	0.0%
k__Bacteria;p__Actinobacteria;c__Actinobacteria;o__Actinomycetales;f__Nocardioidaceae;Other	0.0%	0.0%	0.0%	0.0%	0.0%	0.0%	0.0%
k__Bacteria;p__Actinobacteria;c__Actinobacteria;o__Actinomycetales;f__Nocardioidaceae;g__	0.7%	2.9%	0.2%	0.3%	0.2%	0.3%	0.3%
k__Bacteria;p__Actinobacteria;c__Actinobacteria;o__Actinomycetales;f__Nocardioidaceae;g__Actinopolymorpha	0.0%	0.2%	0.0%	0.0%	0.0%	0.0%	0.0%
k__Bacteria;p__Actinobacteria;c__Actinobacteria;o__Actinomycetales;f__Nocardioidaceae;g__Aeromicrobium	0.3%	1.8%	0.0%	0.0%	0.0%	0.0%	0.0%
k__Bacteria;p__Actinobacteria;c__Actinobacteria;o__Actinomycetales;f__Nocardioidaceae;g__Friedmanniella	0.0%	0.0%	0.0%	0.0%	0.0%	0.0%	0.0%
k__Bacteria;p__Actinobacteria;c__Actinobacteria;o__Actinomycetales;f__Nocardioidaceae;g__Kribbella	0.1%	0.3%	0.0%	0.1%	0.0%	0.0%	0.0%
k__Bacteria;p__Actinobacteria;c__Actinobacteria;o__Actinomycetales;f__Nocardioidaceae;g__Nocardioides	0.1%	0.5%	0.0%	0.1%	0.0%	0.0%	0.0%
k__Bacteria;p__Actinobacteria;c__Actinobacteria;o__Actinomycetales;f__Nocardioidaceae;g__Pimelobacter	0.0%	0.0%	0.0%	0.0%	0.0%	0.0%	0.0%
k__Bacteria;p__Actinobacteria;c__Actinobacteria;o__Actinomycetales;f__Nocardioidaceae;g__Propionicimonas	0.0%	0.0%	0.0%	0.0%	0.0%	0.0%	0.0%
k__Bacteria;p__Actinobacteria;c__Actinobacteria;o__Actinomycetales;f__Nocardiopsaceae;g__Nocardiopsis	0.0%	0.0%	0.0%	0.0%	0.0%	0.0%	0.0%
k__Bacteria;p__Actinobacteria;c__Actinobacteria;o__Actinomycetales;f__Promicromonosporaceae;Other	0.0%	0.0%	0.0%	0.0%	0.0%	0.0%	0.0%
k__Bacteria;p__Actinobacteria;c__Actinobacteria;o__Actinomycetales;f__Promicromonosporaceae;g__Promicromonospora	0.0%	0.0%	0.0%	0.0%	0.0%	0.0%	0.0%
k__Bacteria;p__Actinobacteria;c__Actinobacteria;o__Actinomycetales;f__Promicromonosporaceae;g__Xylanimicrobium	0.0%	0.0%	0.0%	0.0%	0.0%	0.0%	0.0%
k__Bacteria;p__Actinobacteria;c__Actinobacteria;o__Actinomycetales;f__Propionibacteriaceae;Other	0.0%	0.0%	0.0%	0.0%	0.0%	0.0%	0.0%

	k__Bacteria;p__Actinobacteria;c__Actinobacteria;o__Actinomycetales;f__Propionibacteriaceae;g__	0.3%	0.2%	0.1%	0.1%	0.0%	0.4%	0.8%
	k__Bacteria;p__Actinobacteria;c__Actinobacteria;o__Actinomycetales;f__Propionibacteriaceae;g__Luteococcus	0.0%	0.0%	0.0%	0.0%	0.0%	0.0%	0.0%
	k__Bacteria;p__Actinobacteria;c__Actinobacteria;o__Actinomycetales;f__Propionibacteriaceae;g__Microlunatus	0.3%	0.2%	0.0%	0.2%	0.0%	0.1%	1.0%
	k__Bacteria;p__Actinobacteria;c__Actinobacteria;o__Actinomycetales;f__Propionibacteriaceae;g__Propionibacterium	0.0%	0.0%	0.0%	0.0%	0.0%	0.0%	0.0%
	k__Bacteria;p__Actinobacteria;c__Actinobacteria;o__Actinomycetales;f__Pseudonocardiaceae;Other	0.0%	0.1%	0.0%	0.0%	0.0%	0.0%	0.0%
	k__Bacteria;p__Actinobacteria;c__Actinobacteria;o__Actinomycetales;f__Pseudonocardiaceae;g__	0.6%	1.0%	0.0%	0.0%	0.0%	2.6%	0.1%
	k__Bacteria;p__Actinobacteria;c__Actinobacteria;o__Actinomycetales;f__Pseudonocardiaceae;g__Actinomycetospora	0.0%	0.0%	0.0%	0.0%	0.0%	0.0%	0.0%
	k__Bacteria;p__Actinobacteria;c__Actinobacteria;o__Actinomycetales;f__Pseudonocardiaceae;g__Amycolatopsis	0.0%	0.1%	0.0%	0.0%	0.0%	0.0%	0.0%
	k__Bacteria;p__Actinobacteria;c__Actinobacteria;o__Actinomycetales;f__Pseudonocardiaceae;g__Jiangella	0.0%	0.0%	0.0%	0.0%	0.0%	0.0%	0.0%
	k__Bacteria;p__Actinobacteria;c__Actinobacteria;o__Actinomycetales;f__Pseudonocardiaceae;g__Pseudonocardia	0.1%	0.5%	0.0%	0.1%	0.0%	0.1%	0.1%
	k__Bacteria;p__Actinobacteria;c__Actinobacteria;o__Actinomycetales;f__Pseudonocardiaceae;g__Saccharopolyspora	0.0%	0.0%	0.0%	0.0%	0.0%	0.0%	0.0%
	k__Bacteria;p__Actinobacteria;c__Actinobacteria;o__Actinomycetales;f__Pseudonocardiaceae;g__Thermobispora	0.0%	0.0%	0.0%	0.0%	0.0%	0.0%	0.0%
	k__Bacteria;p__Actinobacteria;c__Actinobacteria;o__Actinomycetales;f__Sanguibacteraceae;g__Sanguibacter	0.0%	0.0%	0.0%	0.0%	0.0%	0.0%	0.0%
	k__Bacteria;p__Actinobacteria;c__Actinobacteria;o__Actinomycetales;f__Sporichthyaceae;g__	0.0%	0.0%	0.0%	0.0%	0.0%	0.0%	0.0%
	k__Bacteria;p__Actinobacteria;c__Actinobacteria;o__Actinomycetales;f__Sporichthyaceae;g__Sporichthya	0.0%	0.0%	0.0%	0.0%	0.0%	0.0%	0.0%
	k__Bacteria;p__Actinobacteria;c__Actinobacteria;o__Actinomycetales;f__Streptomycetaceae;Other	0.0%	0.0%	0.0%	0.0%	0.0%	0.0%	0.0%
	k__Bacteria;p__Actinobacteria;c__Actinobacteria;o__Actinomycetales;f__Streptomycetaceae;g__	0.2%	0.4%	0.0%	0.4%	0.0%	0.1%	0.4%
	k__Bacteria;p__Actinobacteria;c__Actinobacteria;o__Actinomycetales;f__Streptomycetaceae;g__Streptacidiphilus	0.0%	0.0%	0.0%	0.0%	0.1%	0.0%	0.0%
	k__Bacteria;p__Actinobacteria;c__Actinobacteria;o__Actinomycetales;f__Streptomycetaceae;g__Streptomyces	0.3%	1.1%	0.0%	0.2%	0.0%	0.1%	0.2%
	k__Bacteria;p__Actinobacteria;c__Actinobacteria;o__Actinomycetales;f__Streptosporangiaceae;Other	0.0%	0.0%	0.0%	0.0%	0.0%	0.0%	0.0%
	k__Bacteria;p__Actinobacteria;c__Actinobacteria;o__Actinomycetales;f__Streptosporangiaceae;g__	0.0%	0.0%	0.0%	0.1%	0.0%	0.0%	0.0%
	k__Bacteria;p__Actinobacteria;c__Actinobacteria;o__Actinomycetales;f__Streptosporangiaceae;g__Nonomuraea	0.0%	0.0%	0.0%	0.0%	0.0%	0.0%	0.0%
	k__Bacteria;p__Actinobacteria;c__Actinobacteria;o__Actinomycetales;f__Streptosporangiaceae;g__Sphaerisporangium	0.0%	0.0%	0.0%	0.0%	0.0%	0.0%	0.0%
	k__Bacteria;p__Actinobacteria;c__Actinobacteria;o__Actinomycetales;f__Streptosporangiaceae;g__Streptosporangium	0.0%	0.0%	0.0%	0.1%	0.0%	0.0%	0.0%
	k__Bacteria;p__Actinobacteria;c__Actinobacteria;o__Actinomycetales;f__Thermomonosporaceae;Other	0.0%	0.0%	0.0%	0.0%	0.0%	0.0%	0.0%
	k__Bacteria;p__Actinobacteria;c__Actinobacteria;o__Actinomycetales;f__Thermomonosporaceae;g__	0.0%	0.0%	0.0%	0.0%	0.0%	0.0%	0.0%
	k__Bacteria;p__Actinobacteria;c__Actinobacteria;o__Actinomycetales;f__Thermomonosporaceae;g__Actinoallomurus	0.0%	0.0%	0.0%	0.0%	0.0%	0.0%	0.0%
	k__Bacteria;p__Actinobacteria;c__Actinobacteria;o__Actinomycetales;f__Thermomonosporaceae;g__Actinocorallia	0.0%	0.0%	0.0%	0.0%	0.0%	0.0%	0.0%
	k__Bacteria;p__Actinobacteria;c__Actinobacteria;o__Actinomycetales;f__Thermomonosporaceae;g__Actinomadura	0.1%	0.3%	0.0%	0.0%	0.0%	0.0%	0.0%
	k__Bacteria;p__Actinobacteria;c__Actinobacteria;o__Actinomycetales;f__Williamsiaceae;g__Williamsia	0.0%	0.0%	0.0%	0.0%	0.0%	0.0%	0.0%
	k__Bacteria;p__Actinobacteria;c__Actinobacteria;o__Bifidobacteriales;f__;g__	0.0%	0.0%	0.0%	0.0%	0.0%	0.0%	0.0%
	k__Bacteria;p__Actinobacteria;c__Actinobacteria;o__Micrococcales;f__;g__	0.1%	0.1%	0.0%	0.0%	0.0%	0.1%	0.2%
	k__Bacteria;p__Actinobacteria;c__Actinobacteria;o__WCHB1-81;f__At425_EubF1;g__	0.0%	0.0%	0.0%	0.0%	0.0%	0.0%	0.0%
	k__Bacteria;p__Actinobacteria;c__Coriobacteriia;o__Coriobacteriales;f__Coriobacteriaceae;g__Adlercreutzia	0.0%	0.0%	0.0%	0.0%	0.0%	0.0%	0.0%
	k__Bacteria;p__Actinobacteria;c__KIST-JJY010;o__;f__;g__	0.0%	0.0%	0.0%	0.0%	0.0%	0.0%	0.0%
	k__Bacteria;p__Actinobacteria;c__MB-A2-108;o__;f__;g__	0.0%	0.0%	0.0%	0.0%	0.0%	0.0%	0.0%
	k__Bacteria;p__Actinobacteria;c__MB-A2-108;o__0319-7L14;f__;g__	1.1%	0.7%	0.6%	0.6%	0.0%	2.8%	1.7%
	k__Bacteria;p__Actinobacteria;c__Nitriliruptoria;o__Euzebyales;f__Euzebyaceae;g__Euzebya	0.0%	0.0%	0.0%	0.0%	0.0%	0.1%	0.0%
	k__Bacteria;p__Actinobacteria;c__OPB41;o__;f__;g__	0.0%	0.0%	0.0%	0.0%	0.0%	0.0%	0.1%
	k__Bacteria;p__Actinobacteria;c__Rubrobacteria;o__Rubrobacterales;f__Rubrobacteraceae;g__	0.1%	0.1%	0.0%	0.0%	0.0%	0.1%	0.2%
	k__Bacteria;p__Actinobacteria;c__Rubrobacteria;o__Rubrobacterales;f__Rubrobacteraceae;g__Rubrobacter	0.2%	0.1%	0.8%	0.0%	0.0%	0.2%	0.2%
	k__Bacteria;p__Actinobacteria;c__Thermoleophilia;o__;f__;g__	0.0%	0.0%	0.0%	0.0%	0.0%	0.0%	0.1%
	k__Bacteria;p__Actinobacteria;c__Thermoleophilia;o__Gaiellales;Other;Other	0.0%	0.0%	0.0%	0.0%	0.0%	0.0%	0.0%
	k__Bacteria;p__Actinobacteria;c__Thermoleophilia;o__Gaiellales;f__;g__	0.0%	0.0%	0.0%	0.1%	0.0%	0.1%	0.1%
	k__Bacteria;p__Actinobacteria;c__Thermoleophilia;o__Gaiellales;f__AK1AB1_02E;g__	0.0%	0.0%	0.0%	0.0%	0.0%	0.0%	0.0%
	k__Bacteria;p__Actinobacteria;c__Thermoleophilia;o__Gaiellales;f__Gaiellaceae;g__	1.7%	1.7%	0.8%	0.9%	0.1%	2.9%	3.8%
	k__Bacteria;p__Actinobacteria;c__Thermoleophilia;o__Solirubrobacterales;Other;Other	0.0%	0.2%	0.0%	0.0%	0.0%	0.0%	0.1%
	k__Bacteria;p__Actinobacteria;c__Thermoleophilia;o__Solirubrobacterales;f__;g__	0.7%	2.1%	0.1%	0.5%	0.0%	0.5%	0.9%
	k__Bacteria;p__Actinobacteria;c__Thermoleophilia;o__Solirubrobacterales;f__Conexibacteraceae;Other	0.0%	0.0%	0.0%	0.0%	0.0%	0.0%	0.0%
	k__Bacteria;p__Actinobacteria;c__Thermoleophilia;o__Solirubrobacterales;f__Conexibacteraceae;g__	0.0%	0.0%	0.0%	0.0%	0.0%	0.0%	0.0%
	k__Bacteria;p__Actinobacteria;c__Thermoleophilia;o__Solirubrobacterales;f__Conexibacteraceae;g__Conexibacter	0.0%	0.0%	0.0%	0.0%	0.0%	0.0%	0.0%
	k__Bacteria;p__Actinobacteria;c__Thermoleophilia;o__Solirubrobacterales;f__Patulibacteraceae;g__	0.0%	0.0%	0.0%	0.0%	0.0%	0.0%	0.0%
	k__Bacteria;p__Actinobacteria;c__Thermoleophilia;o__Solirubrobacterales;f__Patulibacteraceae;g__Patulibacter	0.0%	0.0%	0.0%	0.0%	0.0%	0.0%	0.0%
	k__Bacteria;p__Actinobacteria;c__Thermoleophilia;o__Solirubrobacterales;f__Solirubrobacteraceae;Other	0.0%	0.0%	0.0%	0.0%	0.0%	0.0%	0.0%
	k__Bacteria;p__Actinobacteria;c__Thermoleophilia;o__Solirubrobacterales;f__Solirubrobacteraceae;g__	0.1%	0.5%	0.0%	0.1%	0.0%	0.1%	0.1%
	k__Bacteria;p__Actinobacteria;c__Thermoleophilia;o__Solirubrobacterales;f__Solirubrobacteraceae;g__Solirubrobacter	0.0%	0.0%	0.0%	0.0%	0.0%	0.0%	0.0%
	k__Bacteria;p__Armatimonadetes;c__0319-6E2;o__;f__;g__	0.1%	0.0%	0.2%	0.0%	0.0%	0.1%	0.1%
	k__Bacteria;p__Armatimonadetes;c__Armatimonadia;o__Armatimonadales;f__Armatimonadaceae;g__	0.0%	0.0%	0.0%	0.0%	0.0%	0.0%	0.0%
	k__Bacteria;p__Armatimonadetes;c__Armatimonadia;o__Armatimonadales;f__Armatimonadaceae;g__Armatimonas	0.0%	0.0%	0.0%	0.0%	0.0%	0.0%	0.0%
	k__Bacteria;p__Armatimonadetes;c__Armatimonadia;o__FW68;f__;g__	0.0%	0.0%	0.0%	0.0%	0.0%	0.0%	0.0%
	k__Bacteria;p__Armatimonadetes;c__Chthonomonadetes;o__Chthonomonadales;f__Chthonomonadaceae;g__	0.0%	0.0%	0.1%	0.0%	0.0%	0.0%	0.0%
	k__Bacteria;p__Armatimonadetes;c__Chthonomonadetes;o__Chthonomonadales;f__Chthonomonadaceae;g__Chthonomonas	0.0%	0.0%	0.0%	0.0%	0.0%	0.0%	0.0%
	k__Bacteria;p__Armatimonadetes;c__Chthonomonadetes;o__SJA-22;f__;g__	0.1%	0.0%	0.7%	0.0%	0.0%	0.1%	0.0%
	k__Bacteria;p__Armatimonadetes;c__OPB50;o__;f__;g__	0.0%	0.0%	0.0%	0.0%	0.0%	0.0%	0.0%
	k__Bacteria;p__Armatimonadetes;c__OS-L;o__;f__;g__	0.0%	0.0%	0.0%	0.0%	0.0%	0.0%	0.0%
	k__Bacteria;p__Armatimonadetes;c__SHA-37;o__;f__;g__	0.0%	0.0%	0.0%	0.0%	0.0%	0.0%	0.0%
	k__Bacteria;p__Armatimonadetes;c__SJA-176;o__GAB-B06;f__;g__	0.0%	0.0%	0.0%	0.0%	0.0%	0.0%	0.0%

	k__Bacteria;p__Armatimonadetes;c__SJA-176;o__RB046;f__;g__	0.0%	0.0%	0.0%	0.0%	0.0%	0.0%	0.0%
	k__Bacteria;p__Armatimonadetes;c__[Fimbriimonadia];o__[Fimbriimonadales];f__;g__	0.0%	0.0%	0.1%	0.0%	0.0%	0.0%	0.0%
	k__Bacteria;p__Armatimonadetes;c__[Fimbriimonadia];o__[Fimbriimonadales];f__[Fimbriimonadaceae];g__	0.1%	0.0%	0.3%	0.0%	0.0%	0.0%	0.0%
	k__Bacteria;p__Armatimonadetes;c__[Fimbriimonadia];o__[Fimbriimonadales];f__[Fimbriimonadaceae];g__Fimbriimonas	0.1%	0.0%	0.3%	0.1%	0.0%	0.0%	0.0%
	k__Bacteria;p__BHI80-139;c__;o__;f__;g__	0.0%	0.0%	0.0%	0.0%	0.0%	0.0%	0.0%
	k__Bacteria;p__BRC1;c__NPL-UPA2;o__;f__;g__	0.0%	0.0%	0.0%	0.0%	0.0%	0.0%	0.0%
	k__Bacteria;p__BRC1;c__PRR-11;o__;f__;g__	0.0%	0.0%	0.0%	0.0%	0.0%	0.0%	0.0%
	k__Bacteria;p__Bacteroidetes;Other;Other;Other;Other	0.0%	0.0%	0.1%	0.0%	0.0%	0.0%	0.0%
	k__Bacteria;p__Bacteroidetes;c__;o__;f__;g__	0.0%	0.0%	0.0%	0.0%	0.0%	0.0%	0.0%
	k__Bacteria;p__Bacteroidetes;c__At12OctB3;o__;f__;g__	0.0%	0.0%	0.0%	0.0%	0.0%	0.0%	0.1%
	k__Bacteria;p__Bacteroidetes;c__BME43;o__;f__;g__	0.0%	0.0%	0.0%	0.0%	0.0%	0.0%	0.0%
	k__Bacteria;p__Bacteroidetes;c__Bacteroidia;o__Bacteroidales;f__;g__	0.0%	0.0%	0.0%	0.0%	0.0%	0.0%	0.1%
	k__Bacteria;p__Bacteroidetes;c__Bacteroidia;o__Bacteroidales;f__BA008;g__	0.0%	0.0%	0.0%	0.0%	0.0%	0.0%	0.0%
	k__Bacteria;p__Bacteroidetes;c__Bacteroidia;o__Bacteroidales;f__Rikenellaceae;g__Blvii28	0.0%	0.0%	0.0%	0.0%	0.0%	0.0%	0.0%
	k__Bacteria;p__Bacteroidetes;c__Bacteroidia;o__Bacteroidales;f__S24-7;g__	0.0%	0.0%	0.0%	0.0%	0.0%	0.0%	0.0%
	k__Bacteria;p__Bacteroidetes;c__Cytophagia;o__Cytophagales;Other;Other	0.0%	0.0%	0.0%	0.0%	0.0%	0.0%	0.0%
	k__Bacteria;p__Bacteroidetes;c__Cytophagia;o__Cytophagales;f__;g__	0.0%	0.0%	0.0%	0.0%	0.0%	0.0%	0.0%
	k__Bacteria;p__Bacteroidetes;c__Cytophagia;o__Cytophagales;f__Cyclobacteriaceae;Other	0.0%	0.0%	0.0%	0.0%	0.0%	0.0%	0.0%
	k__Bacteria;p__Bacteroidetes;c__Cytophagia;o__Cytophagales;f__Cyclobacteriaceae;g__	0.0%	0.0%	0.0%	0.0%	0.0%	0.0%	0.0%
	k__Bacteria;p__Bacteroidetes;c__Cytophagia;o__Cytophagales;f__Cyclobacteriaceae;g__Algoriphagus	0.0%	0.0%	0.0%	0.0%	0.0%	0.0%	0.0%
	k__Bacteria;p__Bacteroidetes;c__Cytophagia;o__Cytophagales;f__Cytophagaceae;g__	0.8%	2.7%	0.5%	1.4%	0.0%	0.0%	0.3%
	k__Bacteria;p__Bacteroidetes;c__Cytophagia;o__Cytophagales;f__Cytophagaceae;g__Adhaeribacter	0.1%	0.1%	0.1%	0.1%	0.0%	0.0%	0.1%
	k__Bacteria;p__Bacteroidetes;c__Cytophagia;o__Cytophagales;f__Cytophagaceae;g__Cytophaga	0.0%	0.0%	0.0%	0.0%	0.0%	0.0%	0.0%
	k__Bacteria;p__Bacteroidetes;c__Cytophagia;o__Cytophagales;f__Cytophagaceae;g__Dyadobacter	0.0%	0.0%	0.0%	0.0%	0.0%	0.0%	0.0%
	k__Bacteria;p__Bacteroidetes;c__Cytophagia;o__Cytophagales;f__Cytophagaceae;g__Emticicia	0.0%	0.0%	0.0%	0.0%	0.0%	0.0%	0.0%
	k__Bacteria;p__Bacteroidetes;c__Cytophagia;o__Cytophagales;f__Cytophagaceae;g__Flectobacillus	0.0%	0.0%	0.0%	0.0%	0.0%	0.0%	0.0%
	k__Bacteria;p__Bacteroidetes;c__Cytophagia;o__Cytophagales;f__Cytophagaceae;g__Hymenobacter	0.0%	0.0%	0.0%	0.1%	0.0%	0.0%	0.0%
	k__Bacteria;p__Bacteroidetes;c__Cytophagia;o__Cytophagales;f__Cytophagaceae;g__Larkinella	0.0%	0.0%	0.0%	0.0%	0.0%	0.0%	0.0%
	k__Bacteria;p__Bacteroidetes;c__Cytophagia;o__Cytophagales;f__Cytophagaceae;g__Leadbetterella	0.0%	0.0%	0.0%	0.0%	0.0%	0.0%	0.0%
	k__Bacteria;p__Bacteroidetes;c__Cytophagia;o__Cytophagales;f__Cytophagaceae;g__Pontibacter	0.0%	0.0%	0.0%	0.0%	0.0%	0.0%	0.0%
	k__Bacteria;p__Bacteroidetes;c__Cytophagia;o__Cytophagales;f__Cytophagaceae;g__Rhodocytophaga	0.0%	0.0%	0.0%	0.0%	0.0%	0.0%	0.0%
	k__Bacteria;p__Bacteroidetes;c__Cytophagia;o__Cytophagales;f__Cytophagaceae;g__Runella	0.0%	0.0%	0.0%	0.0%	0.0%	0.0%	0.0%
	k__Bacteria;p__Bacteroidetes;c__Cytophagia;o__Cytophagales;f__Cytophagaceae;g__Spirosoma	0.0%	0.0%	0.0%	0.0%	0.0%	0.0%	0.0%
	k__Bacteria;p__Bacteroidetes;c__Cytophagia;o__Cytophagales;f__Cytophagaceae;g__Sporocytophaga	0.1%	0.1%	0.0%	0.2%	0.0%	0.0%	0.0%
	k__Bacteria;p__Bacteroidetes;c__Cytophagia;o__Cytophagales;f__Flammeovirgaceae;g__	0.0%	0.0%	0.0%	0.0%	0.0%	0.0%	0.0%
	k__Bacteria;p__Bacteroidetes;c__Cytophagia;o__Cytophagales;f__Flammeovirgaceae;g__Fulvivirga	0.0%	0.0%	0.0%	0.0%	0.0%	0.0%	0.0%
	k__Bacteria;p__Bacteroidetes;c__Cytophagia;o__Cytophagales;f__Flammeovirgaceae;g__Roseivirga	0.0%	0.0%	0.0%	0.0%	0.0%	0.0%	0.0%
	k__Bacteria;p__Bacteroidetes;c__Cytophagia;o__Cytophagales;f__[Amoebophilaceae];g__	0.0%	0.0%	0.0%	0.0%	0.0%	0.0%	0.0%
	k__Bacteria;p__Bacteroidetes;c__Cytophagia;o__Cytophagales;f__[Amoebophilaceae];g__Candidatus Amoebophilus	0.0%	0.0%	0.0%	0.0%	0.0%	0.0%	0.0%
	k__Bacteria;p__Bacteroidetes;c__Cytophagia;o__Cytophagales;f__[Amoebophilaceae];g__SC3-56	0.0%	0.0%	0.0%	0.0%	0.0%	0.0%	0.0%
	k__Bacteria;p__Bacteroidetes;c__Flavobacteriia;o__Flavobacteriales;Other;Other	0.0%	0.0%	0.0%	0.0%	0.0%	0.0%	0.0%
	k__Bacteria;p__Bacteroidetes;c__Flavobacteriia;o__Flavobacteriales;f__;g__	0.0%	0.0%	0.0%	0.0%	0.0%	0.0%	0.0%
	k__Bacteria;p__Bacteroidetes;c__Flavobacteriia;o__Flavobacteriales;f__Cryomorphaceae;g__	0.1%	0.1%	0.2%	0.1%	0.0%	0.0%	0.0%
	k__Bacteria;p__Bacteroidetes;c__Flavobacteriia;o__Flavobacteriales;f__Cryomorphaceae;g__Crocinitomix	0.0%	0.0%	0.0%	0.0%	0.0%	0.0%	0.0%
	k__Bacteria;p__Bacteroidetes;c__Flavobacteriia;o__Flavobacteriales;f__Cryomorphaceae;g__Fluviicola	0.0%	0.0%	0.1%	0.0%	0.0%	0.1%	0.0%
	k__Bacteria;p__Bacteroidetes;c__Flavobacteriia;o__Flavobacteriales;f__Cryomorphaceae;g__Owenweeksia	0.0%	0.0%	0.0%	0.0%	0.0%	0.0%	0.0%
	k__Bacteria;p__Bacteroidetes;c__Flavobacteriia;o__Flavobacteriales;f__Flavobacteriaceae;Other	0.0%	0.0%	0.0%	0.0%	0.0%	0.0%	0.0%
	k__Bacteria;p__Bacteroidetes;c__Flavobacteriia;o__Flavobacteriales;f__Flavobacteriaceae;g__	0.0%	0.0%	0.1%	0.0%	0.0%	0.0%	0.0%
	k__Bacteria;p__Bacteroidetes;c__Flavobacteriia;o__Flavobacteriales;f__Flavobacteriaceae;g__Flavobacterium	0.8%	1.2%	0.7%	2.5%	0.0%	0.1%	0.2%
	k__Bacteria;p__Bacteroidetes;c__Flavobacteriia;o__Flavobacteriales;f__Flavobacteriaceae;g__Gillisia	0.0%	0.0%	0.0%	0.0%	0.0%	0.0%	0.0%
	k__Bacteria;p__Bacteroidetes;c__Flavobacteriia;o__Flavobacteriales;f__Flavobacteriaceae;g__Myroides	0.0%	0.0%	0.0%	0.0%	0.0%	0.0%	0.0%
	k__Bacteria;p__Bacteroidetes;c__Flavobacteriia;o__Flavobacteriales;f__[Weeksellaceae];g__Chryseobacterium	0.0%	0.0%	0.0%	0.0%	0.0%	0.0%	0.0%
	k__Bacteria;p__Bacteroidetes;c__Flavobacteriia;o__Flavobacteriales;f__[Weeksellaceae];g__Elizabethkingia	0.0%	0.0%	0.0%	0.0%	0.0%	0.0%	0.0%
	k__Bacteria;p__Bacteroidetes;c__Sphingobacteriia;o__Sphingobacteriales;Other;Other	0.0%	0.0%	0.0%	0.0%	0.0%	0.0%	0.0%
	k__Bacteria;p__Bacteroidetes;c__Sphingobacteriia;o__Sphingobacteriales;f__;g__	1.8%	0.8%	3.0%	4.2%	0.3%	1.7%	0.7%
	k__Bacteria;p__Bacteroidetes;c__Sphingobacteriia;o__Sphingobacteriales;f__Sphingobacteriaceae;Other	0.0%	0.0%	0.0%	0.0%	0.0%	0.0%	0.0%
	k__Bacteria;p__Bacteroidetes;c__Sphingobacteriia;o__Sphingobacteriales;f__Sphingobacteriaceae;g__	0.4%	0.2%	0.8%	0.3%	0.3%	0.5%	0.2%
	k__Bacteria;p__Bacteroidetes;c__Sphingobacteriia;o__Sphingobacteriales;f__Sphingobacteriaceae;g__Olivibacter	0.0%	0.0%	0.0%	0.0%	0.0%	0.0%	0.0%
	k__Bacteria;p__Bacteroidetes;c__Sphingobacteriia;o__Sphingobacteriales;f__Sphingobacteriaceae;g__Pedobacter	0.1%	0.0%	0.4%	0.1%	0.0%	0.0%	0.0%
	k__Bacteria;p__Bacteroidetes;c__Sphingobacteriia;o__Sphingobacteriales;f__Sphingobacteriaceae;g__Solitalea	0.0%	0.0%	0.0%	0.0%	0.0%	0.0%	0.0%
	k__Bacteria;p__Bacteroidetes;c__Sphingobacteriia;o__Sphingobacteriales;f__Sphingobacteriaceae;g__Sphingobacterium	0.0%	0.0%	0.0%	0.0%	0.0%	0.0%	0.0%
	k__Bacteria;p__Bacteroidetes;c__VC2_1_Bac22;o__;f__;g__	0.0%	0.0%	0.0%	0.0%	0.0%	0.0%	0.0%
	k__Bacteria;p__Bacteroidetes;c__[Rhodothermi];o__[Rhodothermales];f__Rhodothermaceae;g__	0.0%	0.0%	0.0%	0.0%	0.0%	0.0%	0.0%
	k__Bacteria;p__Bacteroidetes;c__[Rhodothermi];o__[Rhodothermales];f__Rhodothermaceae;g__Rubricoccus	0.0%	0.0%	0.1%	0.0%	0.0%	0.0%	0.0%
	k__Bacteria;p__Bacteroidetes;c__[Saprospirae];o__[Saprospirales];f__;g__	0.1%	0.1%	0.1%	0.3%	0.0%	0.0%	0.0%
	k__Bacteria;p__Bacteroidetes;c__[Saprospirae];o__[Saprospirales];f__Chitinophagaceae;Other	0.0%	0.0%	0.0%	0.0%	0.0%	0.0%	0.0%
	k__Bacteria;p__Bacteroidetes;c__[Saprospirae];o__[Saprospirales];f__Chitinophagaceae;g__	2.6%	1.7%	3.4%	5.9%	1.0%	1.3%	2.3%

k__Bacteria;p__Bacteroidetes;c__[Saprospirae];o__[Saprospirales];f__Chitinophagaceae;g__Chitinophaga	0.0%	0.0%	0.0%	0.1%	0.0%	0.0%	0.0%
k__Bacteria;p__Bacteroidetes;c__[Saprospirae];o__[Saprospirales];f__Chitinophagaceae;g__Flavihumibacter	0.0%	0.0%	0.1%	0.0%	0.0%	0.0%	0.0%
k__Bacteria;p__Bacteroidetes;c__[Saprospirae];o__[Saprospirales];f__Chitinophagaceae;g__Flavisolibacter	0.3%	0.1%	1.3%	0.1%	0.0%	0.1%	0.5%
k__Bacteria;p__Bacteroidetes;c__[Saprospirae];o__[Saprospirales];f__Chitinophagaceae;g__Niabella	0.0%	0.0%	0.0%	0.0%	0.0%	0.0%	0.0%
k__Bacteria;p__Bacteroidetes;c__[Saprospirae];o__[Saprospirales];f__Chitinophagaceae;g__Niastella	0.0%	0.0%	0.0%	0.0%	0.0%	0.0%	0.0%
k__Bacteria;p__Bacteroidetes;c__[Saprospirae];o__[Saprospirales];f__Chitinophagaceae;g__Sediminibacterium	0.0%	0.0%	0.0%	0.1%	0.0%	0.0%	0.0%
k__Bacteria;p__Bacteroidetes;c__[Saprospirae];o__[Saprospirales];f__Chitinophagaceae;g__Segetibacter	0.0%	0.0%	0.0%	0.0%	0.0%	0.0%	0.0%
k__Bacteria;p__Bacteroidetes;c__[Saprospirae];o__[Saprospirales];f__Saprospiraceae;g__	0.5%	0.8%	0.6%	0.9%	0.0%	0.0%	0.8%
k__Bacteria;p__Bacteroidetes;c__[Saprospirae];o__[Saprospirales];f__Saprospiraceae;g__Haliscomenobacter	0.0%	0.0%	0.0%	0.0%	0.0%	0.0%	0.0%
k__Bacteria;p__Bacteroidetes;c__[Saprospirae];o__[Saprospirales];f__Saprospiraceae;g__Saprospira	0.0%	0.0%	0.0%	0.0%	0.0%	0.0%	0.0%
k__Bacteria;p__Chlamydiae;c__Chlamydiia;o__;f__;g__	0.1%	0.1%	0.0%	0.2%	0.0%	0.0%	0.0%
k__Bacteria;p__Chlamydiae;c__Chlamydiia;o__Chlamydiales;Other;Other	0.0%	0.0%	0.0%	0.0%	0.0%	0.0%	0.0%
k__Bacteria;p__Chlamydiae;c__Chlamydiia;o__Chlamydiales;f__;g__	0.0%	0.0%	0.1%	0.1%	0.0%	0.0%	0.0%
k__Bacteria;p__Chlamydiae;c__Chlamydiia;o__Chlamydiales;f__Chlamydiaceae;g__	0.0%	0.0%	0.0%	0.0%	0.0%	0.0%	0.0%
k__Bacteria;p__Chlamydiae;c__Chlamydiia;o__Chlamydiales;f__Chlamydiaceae;g__Chlamydia	0.0%	0.0%	0.0%	0.0%	0.0%	0.0%	0.0%
k__Bacteria;p__Chlamydiae;c__Chlamydiia;o__Chlamydiales;f__Criblamydiaceae;Other	0.0%	0.0%	0.0%	0.0%	0.0%	0.0%	0.0%
k__Bacteria;p__Chlamydiae;c__Chlamydiia;o__Chlamydiales;f__Criblamydiaceae;g__	0.0%	0.0%	0.0%	0.0%	0.0%	0.0%	0.0%
k__Bacteria;p__Chlamydiae;c__Chlamydiia;o__Chlamydiales;f__Parachlamydiaceae;Other	0.2%	0.2%	0.1%	0.5%	0.1%	0.1%	0.3%
k__Bacteria;p__Chlamydiae;c__Chlamydiia;o__Chlamydiales;f__Parachlamydiaceae;g__	0.0%	0.0%	0.0%	0.1%	0.1%	0.0%	0.0%
k__Bacteria;p__Chlamydiae;c__Chlamydiia;o__Chlamydiales;f__Parachlamydiaceae;g__Candidatus Protochlamydia	0.1%	0.1%	0.0%	0.1%	0.0%	0.0%	0.1%
k__Bacteria;p__Chlamydiae;c__Chlamydiia;o__Chlamydiales;f__Parachlamydiaceae;g__Parachlamydia	0.1%	0.1%	0.0%	0.1%	0.3%	0.0%	0.1%
k__Bacteria;p__Chlamydiae;c__Chlamydiia;o__Chlamydiales;f__Rhabdochlamydiaceae;g__Candidatus Rhabdochlamydia	0.1%	0.0%	0.0%	0.1%	0.2%	0.0%	0.0%
k__Bacteria;p__Chlamydiae;c__Chlamydiia;o__Chlamydiales;f__Simkaniaceae;Other	0.0%	0.0%	0.0%	0.0%	0.0%	0.0%	0.0%
k__Bacteria;p__Chlamydiae;c__Chlamydiia;o__Chlamydiales;f__Simkaniaceae;g__Simkania	0.0%	0.0%	0.0%	0.0%	0.0%	0.0%	0.0%
k__Bacteria;p__Chlamydiae;c__Chlamydiia;o__Chlamydiales;f__Waddliaceae;Other	0.0%	0.0%	0.0%	0.0%	0.0%	0.0%	0.0%
k__Bacteria;p__Chlamydiae;c__Chlamydiia;o__Chlamydiales;f__Waddliaceae;g__	0.0%	0.0%	0.0%	0.0%	0.0%	0.0%	0.0%
k__Bacteria;p__Chlamydiae;c__Chlamydiia;o__Chlamydiales;f__Waddliaceae;g__Waddlia	0.0%	0.0%	0.0%	0.0%	0.0%	0.0%	0.0%
k__Bacteria;p__Chlorobi;c__;o__;f__;g__	0.1%	0.0%	0.2%	0.1%	0.1%	0.0%	0.1%
k__Bacteria;p__Chlorobi;c__BSV26;o__A89;f__;g__	0.0%	0.0%	0.0%	0.0%	0.0%	0.0%	0.0%
k__Bacteria;p__Chlorobi;c__BSV26;o__C20;f__;g__	0.0%	0.0%	0.0%	0.0%	0.0%	0.0%	0.0%
k__Bacteria;p__Chlorobi;c__BSV26;o__PK329;f__;g__	0.0%	0.0%	0.1%	0.1%	0.0%	0.0%	0.1%
k__Bacteria;p__Chlorobi;c__BSV26;o__VC38;f__;g__	0.0%	0.0%	0.0%	0.0%	0.0%	0.0%	0.0%
k__Bacteria;p__Chlorobi;c__Ignavibacteria;o__Ignavibacteriales;f__Ignavibacteriaceae;g__	0.0%	0.0%	0.0%	0.0%	0.0%	0.0%	0.0%
k__Bacteria;p__Chlorobi;c__OPB56;o__;f__;g__	0.1%	0.1%	0.1%	0.0%	0.0%	0.0%	0.1%
k__Bacteria;p__Chlorobi;c__SJA-28;o__;f__;g__	0.2%	0.0%	0.3%	0.2%	0.0%	0.1%	0.6%
k__Bacteria;p__Chloroflexi;Other;Other;Other;Other	0.0%	0.0%	0.0%	0.0%	0.0%	0.0%	0.0%
k__Bacteria;p__Chloroflexi;c__;o__;f__;g__	0.0%	0.1%	0.0%	0.0%	0.0%	0.0%	0.0%
k__Bacteria;p__Chloroflexi;c__Anaerolineae;Other;Other;Other	0.0%	0.0%	0.0%	0.0%	0.0%	0.0%	0.0%
k__Bacteria;p__Chloroflexi;c__Anaerolineae;o__;f__;g__	0.1%	0.0%	0.2%	0.0%	0.0%	0.1%	0.0%
k__Bacteria;p__Chloroflexi;c__Anaerolineae;o__A31;f__;g__	0.0%	0.0%	0.1%	0.0%	0.0%	0.0%	0.0%
k__Bacteria;p__Chloroflexi;c__Anaerolineae;o__A31;f__S47;g__	0.2%	0.0%	0.1%	0.2%	0.0%	0.0%	1.0%
k__Bacteria;p__Chloroflexi;c__Anaerolineae;o__Anaerolineales;f__Anaerolinaceae;g__	0.0%	0.0%	0.0%	0.0%	0.0%	0.0%	0.0%
k__Bacteria;p__Chloroflexi;c__Anaerolineae;o__Anaerolineales;f__Anaerolinaceae;g__Anaerolinea	0.0%	0.0%	0.0%	0.0%	0.0%	0.0%	0.2%
k__Bacteria;p__Chloroflexi;c__Anaerolineae;o__Ardenscatenales;f__Ardenscatenaceae;g__Ardenscatena	0.0%	0.0%	0.1%	0.0%	0.0%	0.0%	0.0%
k__Bacteria;p__Chloroflexi;c__Anaerolineae;o__CFB-26;f__;g__	0.1%	0.1%	0.1%	0.0%	0.0%	0.1%	0.0%
k__Bacteria;p__Chloroflexi;c__Anaerolineae;o__Caldilineales;f__Caldilineaceae;g__	0.2%	0.3%	0.3%	0.1%	0.0%	0.1%	0.5%
k__Bacteria;p__Chloroflexi;c__Anaerolineae;o__Caldilineales;f__Caldilineaceae;g__Caldilinea	0.1%	0.0%	0.0%	0.0%	0.0%	0.0%	0.5%
k__Bacteria;p__Chloroflexi;c__Anaerolineae;o__Caldilineales;f__Caldilineaceae;g__Litorilinea	0.0%	0.0%	0.0%	0.0%	0.0%	0.0%	0.0%
k__Bacteria;p__Chloroflexi;c__Anaerolineae;o__GCA004;f__;g__	0.2%	0.0%	0.0%	0.0%	0.0%	0.0%	1.1%
k__Bacteria;p__Chloroflexi;c__Anaerolineae;o__H39;f__;g__	0.1%	0.1%	0.1%	0.0%	0.0%	0.1%	0.0%
k__Bacteria;p__Chloroflexi;c__Anaerolineae;o__S0208;f__;g__	0.3%	0.1%	0.4%	0.2%	0.0%	0.4%	0.8%
k__Bacteria;p__Chloroflexi;c__Anaerolineae;o__SB-34;f__;g__	0.0%	0.0%	0.0%	0.0%	0.0%	0.0%	0.0%
k__Bacteria;p__Chloroflexi;c__Anaerolineae;o__SBR1031;f__A4b;g__	0.2%	0.3%	0.2%	0.3%	0.0%	0.1%	0.2%
k__Bacteria;p__Chloroflexi;c__Anaerolineae;o__SBR1031;f__SJA-101;g__	0.0%	0.1%	0.0%	0.0%	0.0%	0.0%	0.0%
k__Bacteria;p__Chloroflexi;c__Anaerolineae;o__SBR1031;f__oc28;g__	0.0%	0.0%	0.0%	0.0%	0.0%	0.0%	0.0%
k__Bacteria;p__Chloroflexi;c__Anaerolineae;o__SHA-20;f__;g__	0.5%	0.0%	2.8%	0.0%	0.0%	0.2%	0.0%
k__Bacteria;p__Chloroflexi;c__Anaerolineae;o__SJA-15;f__;g__	0.1%	0.0%	0.0%	0.0%	0.0%	0.0%	0.5%
k__Bacteria;p__Chloroflexi;c__Anaerolineae;o__WCHB1-50;f__;g__	0.2%	0.0%	0.9%	0.1%	0.0%	0.2%	0.2%
k__Bacteria;p__Chloroflexi;c__Anaerolineae;o__envOPS12;f__;g__	0.1%	0.0%	0.1%	0.1%	0.0%	0.0%	0.3%
k__Bacteria;p__Chloroflexi;c__Anaerolineae;o__pLW-97;f__;g__	0.0%	0.0%	0.0%	0.0%	0.0%	0.0%	0.0%
k__Bacteria;p__Chloroflexi;c__C0119;o__;f__;g__	0.1%	0.0%	0.2%	0.1%	0.0%	0.0%	0.1%
k__Bacteria;p__Chloroflexi;c__Chloroflexi;o__AKIW781;f__;g__	0.0%	0.0%	0.0%	0.0%	0.0%	0.0%	0.0%
k__Bacteria;p__Chloroflexi;c__Chloroflexi;o__Chloroflexales;Other;Other	0.0%	0.0%	0.0%	0.0%	0.0%	0.0%	0.0%
k__Bacteria;p__Chloroflexi;c__Chloroflexi;o__Chloroflexales;f__01D2Z48;g__	0.0%	0.0%	0.0%	0.0%	0.0%	0.0%	0.0%
k__Bacteria;p__Chloroflexi;c__Chloroflexi;o__Chloroflexales;f__Chloroflexaceae;Other	0.0%	0.0%	0.0%	0.0%	0.0%	0.0%	0.0%
k__Bacteria;p__Chloroflexi;c__Chloroflexi;o__Chloroflexales;f__Chloroflexaceae;g__	0.0%	0.0%	0.0%	0.0%	0.0%	0.0%	0.0%
k__Bacteria;p__Chloroflexi;c__Chloroflexi;o__Chloroflexales;f__Chloroflexaceae;g__Chloroflexus	0.0%	0.0%	0.0%	0.0%	0.0%	0.0%	0.0%

k__Bacteria;p__Chloroflexi;c__Chloroflexi;o__Chloroflexales;f__Chloroflexaceae;g__Chloronema	0.0%	0.0%	0.0%	0.0%	0.0%	0.0%	0.0%
k__Bacteria;p__Chloroflexi;c__Chloroflexi;o__Chloroflexales;f__FFCH7168;g__	0.0%	0.0%	0.0%	0.0%	0.0%	0.0%	0.0%
k__Bacteria;p__Chloroflexi;c__Chloroflexi;o__Chloroflexales;f__Oscillochloridaceae;g__Oscillochloris	0.0%	0.0%	0.0%	0.0%	0.0%	0.0%	0.0%
k__Bacteria;p__Chloroflexi;c__Chloroflexi;o__Chloroflexales;f__[Chlorothrixaceae];g__	0.0%	0.0%	0.0%	0.0%	0.0%	0.0%	0.0%
k__Bacteria;p__Chloroflexi;c__Chloroflexi;o__Herpetosiphonales;f__;g__	0.0%	0.0%	0.0%	0.0%	0.0%	0.0%	0.0%
k__Bacteria;p__Chloroflexi;c__Chloroflexi;o__[Roseiflexales];Other;Other	0.0%	0.0%	0.0%	0.0%	0.0%	0.0%	0.0%
k__Bacteria;p__Chloroflexi;c__Chloroflexi;o__[Roseiflexales];f__;g__	0.1%	0.0%	0.0%	0.1%	0.0%	0.0%	0.2%
k__Bacteria;p__Chloroflexi;c__Chloroflexi;o__[Roseiflexales];f__[Kouleothrixaceae];g__	0.1%	0.0%	0.1%	0.2%	0.0%	0.1%	0.3%
k__Bacteria;p__Chloroflexi;c__Chloroflexi;o__[Roseiflexales];f__[Roseiflexaceae];g__	0.0%	0.0%	0.0%	0.0%	0.0%	0.0%	0.0%
k__Bacteria;p__Chloroflexi;c__Dehalococcoidetes;Other;Other;Other	0.0%	0.0%	0.0%	0.0%	0.0%	0.0%	0.0%
k__Bacteria;p__Chloroflexi;c__Dehalococcoidetes;o__Dehalococcoidales;f__;g__	0.0%	0.0%	0.0%	0.0%	0.0%	0.0%	0.0%
k__Bacteria;p__Chloroflexi;c__Dehalococcoidetes;o__Dehalococcoidales;f__Dehalococcoidaceae;g__	0.0%	0.0%	0.0%	0.0%	0.0%	0.0%	0.0%
k__Bacteria;p__Chloroflexi;c__Dehalococcoidetes;o__Dehalococcoidales;f__Dehalococcoidaceae;g__Dehalogenimonas	0.0%	0.0%	0.0%	0.0%	0.0%	0.0%	0.0%
k__Bacteria;p__Chloroflexi;c__Ellin6529;o__;f__;g__	0.6%	0.9%	0.3%	0.6%	0.0%	0.3%	1.3%
k__Bacteria;p__Chloroflexi;c__Gitt-GS-136;o__;f__;g__	0.4%	1.5%	0.1%	0.1%	0.0%	0.4%	0.2%
k__Bacteria;p__Chloroflexi;c__Ktedonobacteria;Other;Other;Other	0.0%	0.0%	0.0%	0.0%	0.0%	0.0%	0.0%
k__Bacteria;p__Chloroflexi;c__Ktedonobacteria;o__;f__;g__	0.0%	0.0%	0.0%	0.0%	0.1%	0.0%	0.0%
k__Bacteria;p__Chloroflexi;c__Ktedonobacteria;o__B10-SB3A;f__;g__	0.0%	0.0%	0.0%	0.0%	0.0%	0.0%	0.0%
k__Bacteria;p__Chloroflexi;c__Ktedonobacteria;o__B12-WMSP1;f__;g__	0.0%	0.0%	0.0%	0.0%	0.2%	0.0%	0.0%
k__Bacteria;p__Chloroflexi;c__Ktedonobacteria;o__Elev-1554;f__;g__	0.0%	0.0%	0.0%	0.0%	0.0%	0.0%	0.0%
k__Bacteria;p__Chloroflexi;c__Ktedonobacteria;o__JG30-KF-AS9;f__;g__	0.0%	0.0%	0.0%	0.0%	0.0%	0.0%	0.0%
k__Bacteria;p__Chloroflexi;c__Ktedonobacteria;o__Ktedonobacterales;f__Ktedonobacteraceae;g__	0.0%	0.0%	0.0%	0.0%	0.0%	0.0%	0.0%
k__Bacteria;p__Chloroflexi;c__Ktedonobacteria;o__Thermogemmatisporales;f__Thermogemmatisporaceae;g__	2.5%	0.0%	0.0%	0.1%	15.1%	0.0%	0.0%
k__Bacteria;p__Chloroflexi;c__MVP-21;o__;f__;g__	0.0%	0.0%	0.0%	0.0%	0.0%	0.0%	0.0%
k__Bacteria;p__Chloroflexi;c__P2-11E;o__;f__;g__	0.0%	0.0%	0.0%	0.0%	0.0%	0.1%	0.0%
k__Bacteria;p__Chloroflexi;c__S085;o__;f__;g__	0.8%	1.1%	0.6%	0.5%	0.1%	1.4%	0.9%
k__Bacteria;p__Chloroflexi;c__SAR202;o__;f__;g__	0.1%	0.2%	0.0%	0.0%	0.0%	0.1%	0.0%
k__Bacteria;p__Chloroflexi;c__SHA-26;o__;f__;g__	0.0%	0.0%	0.0%	0.0%	0.0%	0.0%	0.0%
k__Bacteria;p__Chloroflexi;c__TK10;Other;Other;Other	0.0%	0.0%	0.0%	0.0%	0.0%	0.0%	0.0%
k__Bacteria;p__Chloroflexi;c__TK10;o__;f__;g__	0.0%	0.1%	0.1%	0.0%	0.0%	0.1%	0.0%
k__Bacteria;p__Chloroflexi;c__TK10;o__AKYG885;Other;Other	0.0%	0.0%	0.0%	0.0%	0.0%	0.0%	0.0%
k__Bacteria;p__Chloroflexi;c__TK10;o__AKYG885;f__;g__	0.0%	0.0%	0.0%	0.0%	0.0%	0.0%	0.0%
k__Bacteria;p__Chloroflexi;c__TK10;o__AKYG885;f__5B-12;g__	0.1%	0.0%	0.1%	0.0%	0.0%	0.2%	0.1%
k__Bacteria;p__Chloroflexi;c__TK10;o__AKYG885;f__Dolo_23;g__	0.0%	0.1%	0.0%	0.1%	0.0%	0.0%	0.1%
k__Bacteria;p__Chloroflexi;c__TK10;o__B07_WMSP1;f__;g__	0.0%	0.0%	0.0%	0.1%	0.0%	0.0%	0.0%
k__Bacteria;p__Chloroflexi;c__TK10;o__B07_WMSP1;f__FFCH4570;g__	0.0%	0.0%	0.0%	0.0%	0.0%	0.0%	0.0%
k__Bacteria;p__Chloroflexi;c__TK17;o__;f__;g__	0.1%	0.2%	0.1%	0.0%	0.2%	0.1%	0.0%
k__Bacteria;p__Chloroflexi;c__TK17;o__mle1-48;f__;g__	0.2%	0.4%	0.4%	0.2%	0.0%	0.2%	0.0%
k__Bacteria;p__Chloroflexi;c__Thermomicrobia;o__;f__;g__	0.0%	0.0%	0.0%	0.0%	0.0%	0.0%	0.0%
k__Bacteria;p__Chloroflexi;c__Thermomicrobia;o__AKYG1722;f__;g__	0.1%	0.5%	0.1%	0.0%	0.0%	0.1%	0.0%
k__Bacteria;p__Chloroflexi;c__Thermomicrobia;o__Ellin6537;f__;g__	0.0%	0.0%	0.0%	0.0%	0.0%	0.0%	0.0%
k__Bacteria;p__Chloroflexi;c__Thermomicrobia;o__JG30-KF-CM45;f__;g__	0.3%	0.7%	0.2%	0.2%	0.0%	0.2%	0.3%
k__Bacteria;p__Chloroflexi;c__Thermomicrobia;o__Sphaerobacterales;f__;g__	0.0%	0.0%	0.0%	0.0%	0.0%	0.0%	0.0%
k__Bacteria;p__Chloroflexi;c__[Thermobacula];o__[Thermobaculales];f__[Thermobaculaceae];g__Thermobaculum	0.0%	0.0%	0.0%	0.0%	0.0%	0.0%	0.0%
k__Bacteria;p__Cyanobacteria;Other;Other;Other;Other	0.0%	0.0%	0.0%	0.0%	0.0%	0.0%	0.0%
k__Bacteria;p__Cyanobacteria;c__;o__;f__;g__	0.0%	0.0%	0.0%	0.0%	0.0%	0.0%	0.0%
k__Bacteria;p__Cyanobacteria;c__4C0d-2;o__MLE1-12;f__;g__	0.0%	0.0%	0.0%	0.0%	0.1%	0.0%	0.1%
k__Bacteria;p__Cyanobacteria;c__4C0d-2;o__SM1D11;f__;g__	0.0%	0.0%	0.0%	0.0%	0.0%	0.0%	0.0%
k__Bacteria;p__Cyanobacteria;c__4C0d-2;o__SM2F09;f__;g__	0.0%	0.0%	0.0%	0.0%	0.0%	0.0%	0.0%
k__Bacteria;p__Cyanobacteria;c__4C0d-2;o__YS2;f__;g__	0.0%	0.0%	0.0%	0.0%	0.0%	0.0%	0.0%
k__Bacteria;p__Cyanobacteria;c__Chloroplast;o__Chlorophyta;f__;g__	0.0%	0.0%	0.0%	0.0%	0.0%	0.0%	0.0%
k__Bacteria;p__Cyanobacteria;c__Chloroplast;o__Chlorophyta;f__Chlamydomonadaceae;Other	0.0%	0.0%	0.0%	0.0%	0.0%	0.0%	0.0%
k__Bacteria;p__Cyanobacteria;c__Chloroplast;o__Chlorophyta;f__Chlamydomonadaceae;g__	0.0%	0.0%	0.0%	0.0%	0.0%	0.0%	0.0%
k__Bacteria;p__Cyanobacteria;c__Chloroplast;o__Chlorophyta;f__Trebouxiophyceae;Other	0.0%	0.0%	0.0%	0.0%	0.0%	0.0%	0.0%
k__Bacteria;p__Cyanobacteria;c__Chloroplast;o__Chlorophyta;f__Trebouxiophyceae;g__	0.0%	0.0%	0.0%	0.0%	0.0%	0.0%	0.0%
k__Bacteria;p__Cyanobacteria;c__Chloroplast;o__Euglenozoa;f__;g__	0.0%	0.0%	0.0%	0.0%	0.0%	0.0%	0.0%
k__Bacteria;p__Cyanobacteria;c__Chloroplast;o__Stramenopiles;f__;g__	0.0%	0.0%	0.0%	0.1%	0.0%	0.0%	0.2%
k__Bacteria;p__Cyanobacteria;c__Chloroplast;o__Streptophyta;f__;g__	0.0%	0.0%	0.0%	0.0%	0.0%	0.0%	0.0%
k__Bacteria;p__Cyanobacteria;c__ML635J-21;o__;f__;g__	0.1%	0.0%	0.1%	0.1%	0.0%	0.1%	0.1%
k__Bacteria;p__Cyanobacteria;c__Nostocophycideae;o__Nostocales;f__;g__	0.0%	0.0%	0.0%	0.0%	0.0%	0.0%	0.0%
k__Bacteria;p__Cyanobacteria;c__Nostocophycideae;o__Nostocales;f__Nostocaceae;Other	0.0%	0.0%	0.0%	0.0%	0.0%	0.0%	0.0%
k__Bacteria;p__Cyanobacteria;c__Nostocophycideae;o__Nostocales;f__Nostocaceae;g__	0.0%	0.0%	0.0%	0.0%	0.0%	0.0%	0.0%
k__Bacteria;p__Cyanobacteria;c__Nostocophycideae;o__Nostocales;f__Nostocaceae;g__Anabaena	0.0%	0.0%	0.0%	0.0%	0.0%	0.0%	0.0%
k__Bacteria;p__Cyanobacteria;c__Nostocophycideae;o__Nostocales;f__Nostocaceae;g__Nodularia	0.0%	0.0%	0.0%	0.0%	0.0%	0.0%	0.0%
k__Bacteria;p__Cyanobacteria;c__Nostocophycideae;o__Nostocales;f__Nostocaceae;g__Nostoc	0.0%	0.0%	0.0%	0.0%	0.0%	0.0%	0.0%
k__Bacteria;p__Cyanobacteria;c__Nostocophycideae;o__Nostocales;f__Scytonemataceae;g__Scytonema	0.0%	0.0%	0.0%	0.0%	0.0%	0.0%	0.0%
k__Bacteria;p__Cyanobacteria;c__Oscillatoriophycideae;Other;Other;Other	0.0%	0.0%	0.0%	0.0%	0.0%	0.0%	0.0%

k__Bacteria;p__Cyanobacteria;c__Oscillatoriophycideae;o__Chroococcales;Other;Other	0.0%	0.0%	0.0%	0.0%	0.0%	0.0%	0.0%
k__Bacteria;p__Cyanobacteria;c__Oscillatoriophycideae;o__Chroococcales;f__;g__	0.0%	0.0%	0.0%	0.0%	0.0%	0.0%	0.0%
k__Bacteria;p__Cyanobacteria;c__Oscillatoriophycideae;o__Chroococcales;f__Chroococcaceae;Other	0.0%	0.0%	0.0%	0.0%	0.0%	0.0%	0.0%
k__Bacteria;p__Cyanobacteria;c__Oscillatoriophycideae;o__Chroococcales;f__Xenococcaceae;Other	0.0%	0.0%	0.0%	0.0%	0.0%	0.0%	0.0%
k__Bacteria;p__Cyanobacteria;c__Oscillatoriophycideae;o__Chroococcales;f__Xenococcaceae;g__	0.0%	0.0%	0.0%	0.0%	0.0%	0.0%	0.0%
k__Bacteria;p__Cyanobacteria;c__Oscillatoriophycideae;o__Oscillatoriales;f__Phormidiaceae;g__Microcoleus	0.0%	0.0%	0.0%	0.0%	0.0%	0.0%	0.0%
k__Bacteria;p__Cyanobacteria;c__Oscillatoriophycideae;o__Oscillatoriales;f__Phormidiaceae;g__Phormidium	0.0%	0.0%	0.0%	0.0%	0.0%	0.0%	0.0%
k__Bacteria;p__Cyanobacteria;c__Oscillatoriophycideae;o__Oscillatoriales;f__Phormidiaceae;g__Planktothrix	0.0%	0.0%	0.0%	0.0%	0.0%	0.0%	0.0%
k__Bacteria;p__Cyanobacteria;c__Synechococcophycideae;o__Pseudanabaenales;f__Pseudanabaenaceae;Other	0.0%	0.0%	0.0%	0.0%	0.0%	0.0%	0.0%
k__Bacteria;p__Cyanobacteria;c__Synechococcophycideae;o__Pseudanabaenales;f__Pseudanabaenaceae;g__	0.0%	0.0%	0.0%	0.0%	0.0%	0.0%	0.1%
k__Bacteria;p__Cyanobacteria;c__Synechococcophycideae;o__Pseudanabaenales;f__Pseudanabaenaceae;g__Arthronema	0.0%	0.0%	0.0%	0.0%	0.0%	0.0%	0.0%
k__Bacteria;p__Cyanobacteria;c__Synechococcophycideae;o__Pseudanabaenales;f__Pseudanabaenaceae;g__Halomicronema	0.0%	0.0%	0.0%	0.0%	0.0%	0.0%	0.0%
k__Bacteria;p__Cyanobacteria;c__Synechococcophycideae;o__Pseudanabaenales;f__Pseudanabaenaceae;g__Leptolyngbya	0.0%	0.0%	0.0%	0.0%	0.0%	0.0%	0.1%
k__Bacteria;p__Cyanobacteria;c__Synechococcophycideae;o__Pseudanabaenales;f__Pseudanabaenaceae;g__Pseudanabaena	0.0%	0.0%	0.0%	0.0%	0.0%	0.0%	0.0%
k__Bacteria;p__Cyanobacteria;c__Synechococcophycideae;o__Synechococcales;f__Acaryochloridaceae;g__Acaryochloris	0.0%	0.0%	0.0%	0.0%	0.0%	0.0%	0.0%
k__Bacteria;p__Elusimicrobia;c__;o__;f__;g__	0.0%	0.0%	0.0%	0.0%	0.0%	0.0%	0.0%
k__Bacteria;p__Elusimicrobia;c__Elusimicrobia;o__;f__;g__	0.0%	0.0%	0.0%	0.0%	0.0%	0.0%	0.0%
k__Bacteria;p__Elusimicrobia;c__Elusimicrobia;o__Elusimicrobiales;f__;g__	0.1%	0.0%	0.1%	0.0%	0.2%	0.0%	0.0%
k__Bacteria;p__Elusimicrobia;c__Elusimicrobia;o__FAC88;f__;g__	0.2%	0.0%	0.2%	0.2%	0.4%	0.0%	0.2%
k__Bacteria;p__Elusimicrobia;c__Elusimicrobia;o__IIb;f__;g__	0.1%	0.2%	0.2%	0.1%	0.0%	0.0%	0.1%
k__Bacteria;p__Elusimicrobia;c__Elusimicrobia;o__MVP-88;f__;g__	0.0%	0.0%	0.1%	0.0%	0.0%	0.0%	0.0%
k__Bacteria;p__Elusimicrobia;c__Endomicrobia;o__;f__;g__	0.0%	0.0%	0.0%	0.0%	0.0%	0.0%	0.0%
k__Bacteria;p__FBP;c__;o__;f__;g__	0.0%	0.0%	0.0%	0.0%	0.0%	0.0%	0.0%
k__Bacteria;p__FCPU426;c__;o__;f__;g__	0.0%	0.0%	0.0%	0.0%	0.2%	0.0%	0.0%
k__Bacteria;p__Fibrobacteres;c__;o__;f__;g__	0.0%	0.0%	0.0%	0.0%	0.0%	0.0%	0.0%
k__Bacteria;p__Fibrobacteres;c__B5-096;o__;f__;g__	0.0%	0.0%	0.0%	0.0%	0.0%	0.0%	0.0%
k__Bacteria;p__Fibrobacteres;c__Fibrobacteria;o__258ds10;f__;g__	0.0%	0.0%	0.0%	0.0%	0.0%	0.0%	0.0%
k__Bacteria;p__Fibrobacteres;c__Fibrobacteria;o__Ucp1540;f__;g__	0.0%	0.0%	0.2%	0.0%	0.0%	0.0%	0.0%
k__Bacteria;p__Fibrobacteres;c__SBZC_2415;o__;f__;g__	0.0%	0.0%	0.0%	0.0%	0.0%	0.0%	0.0%
k__Bacteria;p__Fibrobacteres;c__TG3;o__TG3-1;f__;g__	0.0%	0.0%	0.0%	0.0%	0.0%	0.0%	0.0%
k__Bacteria;p__Fibrobacteres;c__TG3;o__TG3-1;f__TSCOR003-O20;g__	0.0%	0.0%	0.0%	0.0%	0.0%	0.0%	0.0%
k__Bacteria;p__Firmicutes;Other;Other;Other;Other	0.0%	0.0%	0.0%	0.0%	0.0%	0.0%	0.0%
k__Bacteria;p__Firmicutes;c__Bacilli;Other;Other;Other	0.0%	0.0%	0.0%	0.0%	0.0%	0.0%	0.0%
k__Bacteria;p__Firmicutes;c__Bacilli;o__Bacillales;Other;Other	0.3%	1.3%	0.0%	0.3%	0.1%	0.2%	0.0%
k__Bacteria;p__Firmicutes;c__Bacilli;o__Bacillales;f__;g__	0.1%	0.1%	0.0%	0.0%	0.0%	0.3%	0.0%
k__Bacteria;p__Firmicutes;c__Bacilli;o__Bacillales;f__Alicyclobacillaceae;g__	0.0%	0.0%	0.0%	0.0%	0.0%	0.0%	0.0%
k__Bacteria;p__Firmicutes;c__Bacilli;o__Bacillales;f__Alicyclobacillaceae;g__Alicyclobacillus	0.1%	0.0%	0.0%	0.1%	0.0%	0.0%	0.6%
k__Bacteria;p__Firmicutes;c__Bacilli;o__Bacillales;f__Bacillaceae;Other	0.0%	0.0%	0.0%	0.0%	0.0%	0.0%	0.0%
k__Bacteria;p__Firmicutes;c__Bacilli;o__Bacillales;f__Bacillaceae;g__	0.1%	0.2%	0.0%	0.0%	0.0%	0.2%	0.1%
k__Bacteria;p__Firmicutes;c__Bacilli;o__Bacillales;f__Bacillaceae;g__Anaerobacillus	0.0%	0.0%	0.0%	0.0%	0.0%	0.0%	0.0%
k__Bacteria;p__Firmicutes;c__Bacilli;o__Bacillales;f__Bacillaceae;g__Anoxybacillus	0.0%	0.0%	0.0%	0.0%	0.0%	0.0%	0.0%
k__Bacteria;p__Firmicutes;c__Bacilli;o__Bacillales;f__Bacillaceae;g__Bacillus	5.6%	4.9%	0.7%	1.4%	3.7%	21.4%	1.7%
k__Bacteria;p__Firmicutes;c__Bacilli;o__Bacillales;f__Bacillaceae;g__Geobacillus	0.0%	0.0%	0.0%	0.0%	0.0%	0.0%	0.0%
k__Bacteria;p__Firmicutes;c__Bacilli;o__Bacillales;f__Bacillaceae;g__Natronobacillus	0.0%	0.0%	0.0%	0.0%	0.0%	0.0%	0.0%
k__Bacteria;p__Firmicutes;c__Bacilli;o__Bacillales;f__Bacillaceae;g__Oceanobacillus	0.0%	0.0%	0.0%	0.0%	0.0%	0.0%	0.0%
k__Bacteria;p__Firmicutes;c__Bacilli;o__Bacillales;f__Bacillaceae;g__Pontibacillus	0.0%	0.0%	0.0%	0.0%	0.0%	0.0%	0.0%
k__Bacteria;p__Firmicutes;c__Bacilli;o__Bacillales;f__Bacillaceae;g__Thalassobacillus	0.0%	0.0%	0.0%	0.0%	0.0%	0.0%	0.0%
k__Bacteria;p__Firmicutes;c__Bacilli;o__Bacillales;f__Bacillaceae;g__Virgibacillus	0.0%	0.0%	0.0%	0.0%	0.0%	0.0%	0.0%
k__Bacteria;p__Firmicutes;c__Bacilli;o__Bacillales;f__Listeriaceae;Other	0.0%	0.0%	0.0%	0.0%	0.0%	0.0%	0.0%
k__Bacteria;p__Firmicutes;c__Bacilli;o__Bacillales;f__Paenibacillaceae;Other	0.0%	0.0%	0.0%	0.0%	0.0%	0.0%	0.0%
k__Bacteria;p__Firmicutes;c__Bacilli;o__Bacillales;f__Paenibacillaceae;g__	0.0%	0.0%	0.0%	0.0%	0.0%	0.0%	0.0%
k__Bacteria;p__Firmicutes;c__Bacilli;o__Bacillales;f__Paenibacillaceae;g__Ammoniphilus	0.0%	0.0%	0.0%	0.0%	0.0%	0.2%	0.0%
k__Bacteria;p__Firmicutes;c__Bacilli;o__Bacillales;f__Paenibacillaceae;g__Aneurinibacillus	0.0%	0.0%	0.0%	0.0%	0.0%	0.0%	0.0%
k__Bacteria;p__Firmicutes;c__Bacilli;o__Bacillales;f__Paenibacillaceae;g__Brevibacillus	0.0%	0.0%	0.0%	0.0%	0.0%	0.0%	0.0%
k__Bacteria;p__Firmicutes;c__Bacilli;o__Bacillales;f__Paenibacillaceae;g__Cohnella	0.1%	0.1%	0.0%	0.1%	0.2%	0.1%	0.0%
k__Bacteria;p__Firmicutes;c__Bacilli;o__Bacillales;f__Paenibacillaceae;g__Paenibacillus	0.4%	0.4%	0.1%	0.5%	0.3%	0.9%	0.2%
k__Bacteria;p__Firmicutes;c__Bacilli;o__Bacillales;f__Pasteuriaceae;g__Pasteuria	0.0%	0.0%	0.0%	0.0%	0.0%	0.0%	0.0%
k__Bacteria;p__Firmicutes;c__Bacilli;o__Bacillales;f__Planococcaceae;Other	0.0%	0.0%	0.0%	0.0%	0.0%	0.0%	0.0%
k__Bacteria;p__Firmicutes;c__Bacilli;o__Bacillales;f__Planococcaceae;g__	0.2%	0.5%	0.0%	0.1%	0.0%	0.3%	0.1%
k__Bacteria;p__Firmicutes;c__Bacilli;o__Bacillales;f__Planococcaceae;g__Lysinibacillus	0.0%	0.1%	0.0%	0.0%	0.0%	0.1%	0.0%
k__Bacteria;p__Firmicutes;c__Bacilli;o__Bacillales;f__Planococcaceae;g__Paenisporosarcina	0.1%	0.2%	0.0%	0.1%	0.0%	0.4%	0.0%
k__Bacteria;p__Firmicutes;c__Bacilli;o__Bacillales;f__Planococcaceae;g__Planococcus	0.0%	0.0%	0.0%	0.0%	0.0%	0.0%	0.0%
k__Bacteria;p__Firmicutes;c__Bacilli;o__Bacillales;f__Planococcaceae;g__Planomicrobium	0.0%	0.0%	0.0%	0.0%	0.0%	0.0%	0.0%
k__Bacteria;p__Firmicutes;c__Bacilli;o__Bacillales;f__Planococcaceae;g__Rummeliibacillus	0.0%	0.0%	0.0%	0.0%	0.0%	0.0%	0.0%
k__Bacteria;p__Firmicutes;c__Bacilli;o__Bacillales;f__Planococcaceae;g__Solibacillus	0.1%	0.1%	0.0%	0.0%	0.0%	0.3%	0.0%
k__Bacteria;p__Firmicutes;c__Bacilli;o__Bacillales;f__Planococcaceae;g__Sporosarcina	0.2%	0.4%	0.1%	0.3%	0.0%	0.2%	0.0%
k__Bacteria;p__Firmicutes;c__Bacilli;o__Bacillales;f__Planococcaceae;g__Ureibacillus	0.0%	0.0%	0.0%	0.0%	0.0%	0.0%	0.0%

	k__Bacteria;p__Firmicutes;c__Bacilli;o__Bacillales;f__Sporolactobacillaceae;Other	0.0%	0.0%	0.0%	0.0%	0.0%	0.0%	0.0%
	k__Bacteria;p__Firmicutes;c__Bacilli;o__Bacillales;f__Sporolactobacillaceae;g__Sporolactobacillus	0.0%	0.0%	0.0%	0.0%	0.0%	0.0%	0.0%
	k__Bacteria;p__Firmicutes;c__Bacilli;o__Bacillales;f__Staphylococcaceae;g__Staphylococcus	0.0%	0.0%	0.0%	0.0%	0.0%	0.0%	0.0%
	k__Bacteria;p__Firmicutes;c__Bacilli;o__Bacillales;f__Thermoactinomycetaceae;Other	0.0%	0.0%	0.0%	0.0%	0.0%	0.0%	0.0%
	k__Bacteria;p__Firmicutes;c__Bacilli;o__Bacillales;f__Thermoactinomycetaceae;g__	0.0%	0.0%	0.0%	0.0%	0.0%	0.0%	0.0%
	k__Bacteria;p__Firmicutes;c__Bacilli;o__Bacillales;f__Thermoactinomycetaceae;g__Planifilum	0.0%	0.0%	0.0%	0.0%	0.0%	0.0%	0.0%
	k__Bacteria;p__Firmicutes;c__Bacilli;o__Bacillales;f__Thermoactinomycetaceae;g__Shimazuella	0.0%	0.0%	0.0%	0.0%	0.0%	0.0%	0.0%
	k__Bacteria;p__Firmicutes;c__Bacilli;o__Bacillales;f__[Exiguobacteraceae];g__	0.0%	0.0%	0.0%	0.0%	0.0%	0.0%	0.0%
	k__Bacteria;p__Firmicutes;c__Bacilli;o__Bacillales;f__[Exiguobacteraceae];g__Exiguobacterium	0.0%	0.0%	0.0%	0.0%	0.0%	0.0%	0.0%
	k__Bacteria;p__Firmicutes;c__Bacilli;o__Bacillales;f__[Thermicanaceae];g__Thermicanus	0.0%	0.0%	0.0%	0.0%	0.0%	0.0%	0.0%
	k__Bacteria;p__Firmicutes;c__Bacilli;o__Haloplasmatales;f__Haloplasmataceae;g__	0.0%	0.0%	0.0%	0.0%	0.0%	0.0%	0.0%
	k__Bacteria;p__Firmicutes;c__Bacilli;o__Lactobacillales;f__;g__	0.0%	0.0%	0.0%	0.0%	0.0%	0.0%	0.0%
	k__Bacteria;p__Firmicutes;c__Bacilli;o__Lactobacillales;f__Aerococcaceae;g__Facklamia	0.0%	0.0%	0.0%	0.0%	0.0%	0.0%	0.0%
	k__Bacteria;p__Firmicutes;c__Bacilli;o__Lactobacillales;f__Carnobacteriaceae;g__Carnobacterium	0.0%	0.0%	0.0%	0.0%	0.0%	0.0%	0.0%
	k__Bacteria;p__Firmicutes;c__Bacilli;o__Lactobacillales;f__Carnobacteriaceae;g__Desemzia	0.0%	0.0%	0.0%	0.0%	0.0%	0.0%	0.0%
	k__Bacteria;p__Firmicutes;c__Bacilli;o__Lactobacillales;f__Enterococcaceae;g__Enterococcus	0.0%	0.0%	0.0%	0.0%	0.0%	0.0%	0.0%
	k__Bacteria;p__Firmicutes;c__Bacilli;o__Lactobacillales;f__Lactobacillaceae;g__Lactobacillus	0.0%	0.0%	0.0%	0.0%	0.0%	0.0%	0.0%
	k__Bacteria;p__Firmicutes;c__Bacilli;o__Lactobacillales;f__Leuconostocaceae;g__Leuconostoc	0.0%	0.0%	0.0%	0.0%	0.0%	0.0%	0.0%
	k__Bacteria;p__Firmicutes;c__Bacilli;o__Turicibacterales;f__Turicibacteraceae;g__Turicibacter	0.0%	0.0%	0.0%	0.0%	0.0%	0.1%	0.0%
	k__Bacteria;p__Firmicutes;c__Clostridia;o__;f__;g__	0.0%	0.0%	0.0%	0.0%	0.0%	0.0%	0.0%
	k__Bacteria;p__Firmicutes;c__Clostridia;o__Clostridiales;Other;Other	0.0%	0.0%	0.0%	0.0%	0.0%	0.0%	0.0%
	k__Bacteria;p__Firmicutes;c__Clostridia;o__Clostridiales;f__;g__	0.0%	0.0%	0.0%	0.0%	0.0%	0.0%	0.0%
	k__Bacteria;p__Firmicutes;c__Clostridia;o__Clostridiales;f__Caldicoprobacteraceae;g__Caldicoprobacter	0.0%	0.0%	0.0%	0.0%	0.0%	0.0%	0.0%
	k__Bacteria;p__Firmicutes;c__Clostridia;o__Clostridiales;f__Christensenellaceae;g__	0.0%	0.0%	0.0%	0.0%	0.0%	0.0%	0.0%
	k__Bacteria;p__Firmicutes;c__Clostridia;o__Clostridiales;f__Clostridiaceae;Other	0.0%	0.0%	0.0%	0.0%	0.0%	0.0%	0.0%
	k__Bacteria;p__Firmicutes;c__Clostridia;o__Clostridiales;f__Clostridiaceae;g__	0.0%	0.1%	0.0%	0.0%	0.0%	0.0%	0.0%
	k__Bacteria;p__Firmicutes;c__Clostridia;o__Clostridiales;f__Clostridiaceae;g__Alkaliphilus	0.0%	0.0%	0.0%	0.0%	0.0%	0.0%	0.0%
	k__Bacteria;p__Firmicutes;c__Clostridia;o__Clostridiales;f__Clostridiaceae;g__Caloramator	0.0%	0.0%	0.0%	0.0%	0.0%	0.0%	0.0%
	k__Bacteria;p__Firmicutes;c__Clostridia;o__Clostridiales;f__Clostridiaceae;g__Candidatus Arthromitus	0.0%	0.0%	0.0%	0.0%	0.0%	0.0%	0.0%
	k__Bacteria;p__Firmicutes;c__Clostridia;o__Clostridiales;f__Clostridiaceae;g__Clostridium	0.1%	0.1%	0.1%	0.1%	0.0%	0.1%	0.3%
	k__Bacteria;p__Firmicutes;c__Clostridia;o__Clostridiales;f__Clostridiaceae;g__Geosporobacter_Thermotalea	0.0%	0.0%	0.0%	0.0%	0.0%	0.0%	0.0%
	k__Bacteria;p__Firmicutes;c__Clostridia;o__Clostridiales;f__Clostridiaceae;g__Oxobacter	0.0%	0.0%	0.0%	0.0%	0.0%	0.0%	0.0%
	k__Bacteria;p__Firmicutes;c__Clostridia;o__Clostridiales;f__Clostridiaceae;g__SMB53	0.0%	0.0%	0.0%	0.0%	0.0%	0.0%	0.0%
	k__Bacteria;p__Firmicutes;c__Clostridia;o__Clostridiales;f__Clostridiaceae;g__Sarcina	0.0%	0.0%	0.0%	0.0%	0.0%	0.0%	0.0%
	k__Bacteria;p__Firmicutes;c__Clostridia;o__Clostridiales;f__Clostridiaceae;g__Thermoanaerobacterium	0.0%	0.0%	0.0%	0.0%	0.0%	0.0%	0.0%
	k__Bacteria;p__Firmicutes;c__Clostridia;o__Clostridiales;f__Dehalobacteriaceae;g__	0.0%	0.0%	0.0%	0.0%	0.0%	0.0%	0.0%
	k__Bacteria;p__Firmicutes;c__Clostridia;o__Clostridiales;f__Dehalobacteriaceae;g__Dehalobacterium	0.0%	0.0%	0.0%	0.0%	0.0%	0.1%	0.0%
	k__Bacteria;p__Firmicutes;c__Clostridia;o__Clostridiales;f__EtOH8;g__	0.0%	0.0%	0.0%	0.0%	0.0%	0.0%	0.0%
	k__Bacteria;p__Firmicutes;c__Clostridia;o__Clostridiales;f__Eubacteriaceae;g__	0.0%	0.0%	0.0%	0.0%	0.0%	0.0%	0.0%
	k__Bacteria;p__Firmicutes;c__Clostridia;o__Clostridiales;f__Gracilibacteraceae;g__	0.0%	0.0%	0.0%	0.0%	0.0%	0.0%	0.1%
	k__Bacteria;p__Firmicutes;c__Clostridia;o__Clostridiales;f__Gracilibacteraceae;g__Gracilibacter	0.0%	0.0%	0.0%	0.0%	0.0%	0.0%	0.0%
	k__Bacteria;p__Firmicutes;c__Clostridia;o__Clostridiales;f__Gracilibacteraceae;g__Lutispora	0.0%	0.0%	0.0%	0.0%	0.0%	0.0%	0.0%
	k__Bacteria;p__Firmicutes;c__Clostridia;o__Clostridiales;f__Lachnospiraceae;Other	0.0%	0.0%	0.0%	0.0%	0.0%	0.0%	0.0%
	k__Bacteria;p__Firmicutes;c__Clostridia;o__Clostridiales;f__Lachnospiraceae;g__	0.0%	0.0%	0.0%	0.0%	0.0%	0.0%	0.0%
	k__Bacteria;p__Firmicutes;c__Clostridia;o__Clostridiales;f__Lachnospiraceae;g__Anaerostipes	0.0%	0.0%	0.0%	0.0%	0.0%	0.0%	0.0%
	k__Bacteria;p__Firmicutes;c__Clostridia;o__Clostridiales;f__Lachnospiraceae;g__Blautia	0.0%	0.0%	0.0%	0.0%	0.0%	0.0%	0.0%
	k__Bacteria;p__Firmicutes;c__Clostridia;o__Clostridiales;f__Lachnospiraceae;g__Coprococcus	0.1%	0.0%	0.0%	0.1%	0.0%	0.5%	0.0%
	k__Bacteria;p__Firmicutes;c__Clostridia;o__Clostridiales;f__Lachnospiraceae;g__Dorea	0.0%	0.0%	0.0%	0.0%	0.0%	0.0%	0.0%
	k__Bacteria;p__Firmicutes;c__Clostridia;o__Clostridiales;f__Lachnospiraceae;g__Epulopiscium	0.0%	0.0%	0.0%	0.0%	0.0%	0.1%	0.0%
	k__Bacteria;p__Firmicutes;c__Clostridia;o__Clostridiales;f__Lachnospiraceae;g__Lachnospira	0.0%	0.0%	0.0%	0.0%	0.0%	0.0%	0.0%
	k__Bacteria;p__Firmicutes;c__Clostridia;o__Clostridiales;f__Lachnospiraceae;g__Roseburia	0.0%	0.0%	0.0%	0.0%	0.0%	0.0%	0.0%
	k__Bacteria;p__Firmicutes;c__Clostridia;o__Clostridiales;f__Peptococcaceae;Other	0.0%	0.0%	0.0%	0.0%	0.0%	0.0%	0.0%
	k__Bacteria;p__Firmicutes;c__Clostridia;o__Clostridiales;f__Peptococcaceae;g__	0.0%	0.0%	0.0%	0.0%	0.0%	0.1%	0.0%
	k__Bacteria;p__Firmicutes;c__Clostridia;o__Clostridiales;f__Peptococcaceae;g__Dehalobacter_Syntrophobotulus	0.0%	0.0%	0.0%	0.0%	0.0%	0.0%	0.0%
	k__Bacteria;p__Firmicutes;c__Clostridia;o__Clostridiales;f__Peptococcaceae;g__Desulfonispora	0.0%	0.0%	0.0%	0.0%	0.0%	0.0%	0.0%
	k__Bacteria;p__Firmicutes;c__Clostridia;o__Clostridiales;f__Peptococcaceae;g__Desulfosporosinus	0.0%	0.0%	0.0%	0.0%	0.0%	0.1%	0.0%
	k__Bacteria;p__Firmicutes;c__Clostridia;o__Clostridiales;f__Peptococcaceae;g__Desulfotomaculum	0.0%	0.0%	0.0%	0.0%	0.0%	0.0%	0.0%
	k__Bacteria;p__Firmicutes;c__Clostridia;o__Clostridiales;f__Peptococcaceae;g__Desulfurispora	0.0%	0.0%	0.0%	0.0%	0.0%	0.0%	0.0%
	k__Bacteria;p__Firmicutes;c__Clostridia;o__Clostridiales;f__Peptococcaceae;g__Pelotomaculum	0.0%	0.0%	0.0%	0.0%	0.0%	0.0%	0.0%
	k__Bacteria;p__Firmicutes;c__Clostridia;o__Clostridiales;f__Peptococcaceae;g__Sporotomaculum	0.0%	0.0%	0.0%	0.0%	0.0%	0.0%	0.0%
	k__Bacteria;p__Firmicutes;c__Clostridia;o__Clostridiales;f__Peptococcaceae;g__WCHB1-84	0.0%	0.0%	0.0%	0.0%	0.0%	0.1%	0.0%
	k__Bacteria;p__Firmicutes;c__Clostridia;o__Clostridiales;f__Peptostreptococcaceae;Other	0.0%	0.1%	0.0%	0.1%	0.0%	0.0%	0.1%
	k__Bacteria;p__Firmicutes;c__Clostridia;o__Clostridiales;f__Peptostreptococcaceae;g__	0.1%	0.1%	0.0%	0.1%	0.0%	0.1%	0.0%
	k__Bacteria;p__Firmicutes;c__Clostridia;o__Clostridiales;f__Peptostreptococcaceae;g__Clostridium	0.0%	0.0%	0.0%	0.0%	0.0%	0.0%	0.0%
	k__Bacteria;p__Firmicutes;c__Clostridia;o__Clostridiales;f__Peptostreptococcaceae;g__Tepidibacter	0.0%	0.0%	0.0%	0.0%	0.0%	0.0%	0.1%
	k__Bacteria;p__Firmicutes;c__Clostridia;o__Clostridiales;f__Ruminococcaceae;Other	0.0%	0.0%	0.0%	0.0%	0.0%	0.0%	0.0%
	k__Bacteria;p__Firmicutes;c__Clostridia;o__Clostridiales;f__Ruminococcaceae;g__	0.1%	0.1%	0.0%	0.1%	0.0%	0.1%	0.3%

k__Bacteria;p__Firmicutes;c__Clostridia;o__Clostridiales;f__Ruminococcaceae;g__Anaerotruncus	0.0%	0.0%	0.0%	0.0%	0.0%	0.0%	0.0%
k__Bacteria;p__Firmicutes;c__Clostridia;o__Clostridiales;f__Ruminococcaceae;g__Ethanoligenens	0.0%	0.0%	0.0%	0.0%	0.0%	0.0%	0.0%
k__Bacteria;p__Firmicutes;c__Clostridia;o__Clostridiales;f__Ruminococcaceae;g__Oscillospira	0.0%	0.0%	0.0%	0.0%	0.0%	0.0%	0.0%
k__Bacteria;p__Firmicutes;c__Clostridia;o__Clostridiales;f__Ruminococcaceae;g__Ruminococcus	0.0%	0.0%	0.0%	0.0%	0.0%	0.0%	0.0%
k__Bacteria;p__Firmicutes;c__Clostridia;o__Clostridiales;f__Symbiobacteriaceae;g__Symbiobacterium	0.0%	0.0%	0.0%	0.0%	0.0%	0.0%	0.0%
k__Bacteria;p__Firmicutes;c__Clostridia;o__Clostridiales;f__Syntrophomonadaceae;g__Syntrophomonas	0.0%	0.0%	0.0%	0.0%	0.0%	0.0%	0.0%
k__Bacteria;p__Firmicutes;c__Clostridia;o__Clostridiales;f__Veillonellaceae;g__	0.0%	0.0%	0.0%	0.0%	0.0%	0.0%	0.0%
k__Bacteria;p__Firmicutes;c__Clostridia;o__Clostridiales;f__Veillonellaceae;g__BSV43	0.0%	0.0%	0.0%	0.0%	0.0%	0.0%	0.0%
k__Bacteria;p__Firmicutes;c__Clostridia;o__Clostridiales;f__Veillonellaceae;g__G07	0.0%	0.0%	0.0%	0.0%	0.0%	0.0%	0.0%
k__Bacteria;p__Firmicutes;c__Clostridia;o__Clostridiales;f__Veillonellaceae;g__Pelosinus	0.0%	0.0%	0.0%	0.0%	0.0%	0.0%	0.0%
k__Bacteria;p__Firmicutes;c__Clostridia;o__Clostridiales;f__Veillonellaceae;g__Sporomusa	0.0%	0.0%	0.0%	0.0%	0.0%	0.0%	0.0%
k__Bacteria;p__Firmicutes;c__Clostridia;o__Clostridiales;f__[Acidaminobacteraceae];g__Acidaminobacter	0.0%	0.0%	0.0%	0.0%	0.0%	0.0%	0.0%
k__Bacteria;p__Firmicutes;c__Clostridia;o__Clostridiales;f__[Acidaminobacteraceae];g__Fusibacter	0.0%	0.0%	0.0%	0.0%	0.0%	0.0%	0.0%
k__Bacteria;p__Firmicutes;c__Clostridia;o__Clostridiales;f__[Mogibacteriaceae];Other	0.0%	0.0%	0.0%	0.0%	0.0%	0.0%	0.0%
k__Bacteria;p__Firmicutes;c__Clostridia;o__Clostridiales;f__[Mogibacteriaceae];g__	0.0%	0.0%	0.0%	0.0%	0.0%	0.0%	0.0%
k__Bacteria;p__Firmicutes;c__Clostridia;o__Clostridiales;f__[Mogibacteriaceae];g__Anaerovorax	0.0%	0.0%	0.0%	0.0%	0.0%	0.0%	0.0%
k__Bacteria;p__Firmicutes;c__Clostridia;o__Clostridiales;f__[Tissierellaceae];g__	0.0%	0.0%	0.0%	0.0%	0.0%	0.0%	0.0%
k__Bacteria;p__Firmicutes;c__Clostridia;o__Clostridiales;f__[Tissierellaceae];g__Sedimentibacter	0.0%	0.0%	0.0%	0.0%	0.0%	0.0%	0.0%
k__Bacteria;p__Firmicutes;c__Clostridia;o__Clostridiales;f__[Tissierellaceae];g__Sporanaerobacter	0.0%	0.0%	0.0%	0.0%	0.0%	0.0%	0.0%
k__Bacteria;p__Firmicutes;c__Clostridia;o__Clostridiales;f__[Tissierellaceae];g__Tepidimicrobium	0.0%	0.0%	0.0%	0.0%	0.0%	0.0%	0.0%
k__Bacteria;p__Firmicutes;c__Clostridia;o__Clostridiales;f__[Tissierellaceae];g__Tissierella_Soehngenia	0.0%	0.0%	0.0%	0.0%	0.0%	0.0%	0.0%
k__Bacteria;p__Firmicutes;c__Clostridia;o__Halanaerobiales;f__;g__	0.0%	0.0%	0.0%	0.0%	0.0%	0.0%	0.0%
k__Bacteria;p__Firmicutes;c__Clostridia;o__OPB54;f__;g__	0.0%	0.0%	0.0%	0.0%	0.0%	0.0%	0.1%
k__Bacteria;p__Firmicutes;c__Clostridia;o__SHA-98;f__D2;g__	0.0%	0.0%	0.0%	0.0%	0.0%	0.0%	0.0%
k__Bacteria;p__Firmicutes;c__Clostridia;o__Thermoanaerobacterales;f__;g__	0.0%	0.0%	0.0%	0.0%	0.0%	0.0%	0.0%
k__Bacteria;p__Firmicutes;c__Clostridia;o__Thermoanaerobacterales;f__Thermoanaerobacteraceae;g__Thermacetogenium	0.0%	0.0%	0.0%	0.0%	0.0%	0.0%	0.0%
k__Bacteria;p__Firmicutes;c__Erysipelotrichi;o__Erysipelotrichales;f__Erysipelotrichaceae;g__	0.0%	0.0%	0.0%	0.0%	0.0%	0.0%	0.0%
k__Bacteria;p__Firmicutes;c__Erysipelotrichi;o__Erysipelotrichales;f__Erysipelotrichaceae;g__PSB-M-3	0.0%	0.0%	0.0%	0.0%	0.0%	0.0%	0.0%
k__Bacteria;p__GAL15;c__;o__;f__;g__	0.4%	0.0%	0.2%	0.0%	1.5%	0.7%	0.0%
k__Bacteria;p__GN02;c__;o__;f__;g__	0.0%	0.0%	0.0%	0.0%	0.0%	0.0%	0.0%
k__Bacteria;p__GN02;c__3BR-5F;o__;f__;g__	0.0%	0.0%	0.0%	0.0%	0.0%	0.0%	0.0%
k__Bacteria;p__GN02;c__BB34;o__;f__;g__	0.0%	0.0%	0.0%	0.0%	0.0%	0.0%	0.0%
k__Bacteria;p__GN02;c__BD1-5;o__;f__;g__	0.0%	0.0%	0.0%	0.0%	0.0%	0.0%	0.0%
k__Bacteria;p__GN02;c__GKS2-174;o__;f__;g__	0.0%	0.0%	0.1%	0.0%	0.0%	0.0%	0.0%
k__Bacteria;p__GN02;c__GN07;o__;f__;g__	0.0%	0.0%	0.0%	0.0%	0.1%	0.0%	0.0%
k__Bacteria;p__GN04;c__;o__;f__;g__	0.0%	0.0%	0.2%	0.0%	0.0%	0.0%	0.0%
k__Bacteria;p__GN04;c__GN15;o__;f__;g__	0.0%	0.0%	0.1%	0.0%	0.0%	0.0%	0.0%
k__Bacteria;p__GN04;c__MSB-5A5;o__;f__;g__	0.0%	0.0%	0.0%	0.0%	0.0%	0.0%	0.0%
k__Bacteria;p__Gemmatimonadetes;c__;o__;f__;g__	0.3%	0.0%	0.7%	0.0%	0.0%	0.7%	0.2%
k__Bacteria;p__Gemmatimonadetes;c__Gemm-1;o__;f__;g__	0.7%	0.7%	0.8%	0.6%	0.2%	1.6%	0.5%
k__Bacteria;p__Gemmatimonadetes;c__Gemm-2;o__;f__;g__	0.1%	0.2%	0.1%	0.0%	0.0%	0.0%	0.1%
k__Bacteria;p__Gemmatimonadetes;c__Gemm-3;o__;f__;g__	0.1%	0.5%	0.1%	0.0%	0.0%	0.0%	0.0%
k__Bacteria;p__Gemmatimonadetes;c__Gemm-4;o__;f__;g__	0.0%	0.0%	0.0%	0.0%	0.0%	0.0%	0.0%
k__Bacteria;p__Gemmatimonadetes;c__Gemm-5;o__;f__;g__	0.2%	0.4%	0.3%	0.0%	0.0%	0.1%	0.3%
k__Bacteria;p__Gemmatimonadetes;c__Gemmatimonadetes;Other;Other;Other	0.0%	0.0%	0.0%	0.0%	0.0%	0.0%	0.0%
k__Bacteria;p__Gemmatimonadetes;c__Gemmatimonadetes;o__;f__;g__	0.3%	0.1%	1.0%	0.1%	0.1%	0.6%	0.2%
k__Bacteria;p__Gemmatimonadetes;c__Gemmatimonadetes;o__C114;f__;g__	0.2%	0.0%	0.5%	0.0%	0.0%	0.6%	0.0%
k__Bacteria;p__Gemmatimonadetes;c__Gemmatimonadetes;o__Ellin5290;f__;g__	0.0%	0.1%	0.0%	0.1%	0.0%	0.0%	0.0%
k__Bacteria;p__Gemmatimonadetes;c__Gemmatimonadetes;o__Gemmatimonadales;Other;Other	0.0%	0.0%	0.0%	0.0%	0.0%	0.0%	0.0%
k__Bacteria;p__Gemmatimonadetes;c__Gemmatimonadetes;o__Gemmatimonadales;f__;g__	0.0%	0.0%	0.0%	0.0%	0.0%	0.0%	0.0%
k__Bacteria;p__Gemmatimonadetes;c__Gemmatimonadetes;o__Gemmatimonadales;f__Ellin5301;g__	0.0%	0.0%	0.0%	0.1%	0.0%	0.0%	0.0%
k__Bacteria;p__Gemmatimonadetes;c__Gemmatimonadetes;o__Gemmatimonadales;f__Gemmatimonadaceae;g__Gemmatimonas	0.1%	0.0%	0.3%	0.1%	0.0%	0.1%	0.0%
k__Bacteria;p__Gemmatimonadetes;c__Gemmatimonadetes;o__KD8-87;f__;g__	0.0%	0.1%	0.1%	0.0%	0.0%	0.0%	0.0%
k__Bacteria;p__Gemmatimonadetes;c__Gemmatimonadetes;o__N1423WL;f__;g__	0.3%	0.0%	1.8%	0.0%	0.0%	0.3%	0.0%
k__Bacteria;p__Kazan-3B-28;c__;o__;f__;g__	0.0%	0.0%	0.0%	0.0%	0.0%	0.0%	0.0%
k__Bacteria;p__Lentisphaerae;c__[Lentisphaeria];o__Lentisphaerales;f__Arctic95B-10;g__	0.0%	0.0%	0.0%	0.0%	0.0%	0.0%	0.0%
k__Bacteria;p__Lentisphaerae;c__[Lentisphaeria];o__Victivallales;f__Victivallaceae;g__	0.0%	0.0%	0.0%	0.0%	0.0%	0.0%	0.0%
k__Bacteria;p__MVP-21;c__;o__;f__;g__	0.0%	0.0%	0.0%	0.0%	0.0%	0.0%	0.0%
k__Bacteria;p__NC10;c__12-24;Other;Other;Other	0.0%	0.0%	0.0%	0.0%	0.0%	0.0%	0.0%
k__Bacteria;p__NC10;c__12-24;o__JH-WHS47;f__;g__	0.0%	0.0%	0.0%	0.0%	0.0%	0.0%	0.1%
k__Bacteria;p__NC10;c__12-24;o__Methylomirabiliales;f__Methylomirabiliaceae;g__Candidatus Methylomirabilis	0.0%	0.0%	0.0%	0.0%	0.0%	0.0%	0.0%
k__Bacteria;p__NC10;c__wb1-A12;o__;f__;g__	0.0%	0.0%	0.0%	0.0%	0.0%	0.0%	0.0%
k__Bacteria;p__NKB19;Other;Other;Other;Other	0.0%	0.0%	0.0%	0.0%	0.0%	0.0%	0.0%
k__Bacteria;p__NKB19;c__;o__;f__;g__	0.0%	0.0%	0.1%	0.0%	0.0%	0.0%	0.0%
k__Bacteria;p__NKB19;c__TSBW08;o__;f__;g__	0.0%	0.0%	0.0%	0.0%	0.0%	0.0%	0.0%
k__Bacteria;p__Nitrospirae;c__Nitrospira;o__Nitrospirales;f__;g__	0.0%	0.0%	0.0%	0.0%	0.0%	0.0%	0.1%
k__Bacteria;p__Nitrospirae;c__Nitrospira;o__Nitrospirales;f__0319-6A21;g__	0.6%	0.1%	0.5%	0.7%	0.3%	1.1%	1.1%

k__Bacteria;p__Nitrospirae;c__Nitrospira;o__Nitrospirales;f__FW;g__4-29	0.0%	0.0%	0.0%	0.0%	0.0%	0.0%	0.2%
k__Bacteria;p__Nitrospirae;c__Nitrospira;o__Nitrospirales;f__Nitrospiraceae;Other	0.0%	0.0%	0.0%	0.0%	0.0%	0.0%	0.0%
k__Bacteria;p__Nitrospirae;c__Nitrospira;o__Nitrospirales;f__Nitrospiraceae;g__	1.6%	0.0%	3.1%	0.1%	4.6%	2.0%	0.0%
k__Bacteria;p__Nitrospirae;c__Nitrospira;o__Nitrospirales;f__Nitrospiraceae;g__JG37-AG-70	0.0%	0.0%	0.0%	0.0%	0.0%	0.0%	0.0%
k__Bacteria;p__Nitrospirae;c__Nitrospira;o__Nitrospirales;f__Nitrospiraceae;g__Nitrospira	3.6%	0.8%	6.0%	1.5%	9.5%	2.0%	2.0%
k__Bacteria;p__Nitrospirae;c__Nitrospira;o__Nitrospirales;f__[Leptospirillaceae];g__	0.0%	0.0%	0.0%	0.0%	0.0%	0.0%	0.0%
k__Bacteria;p__Nitrospirae;c__Nitrospira;o__Nitrospirales;f__[Thermodesulfovibrionaceae];g__	0.0%	0.0%	0.0%	0.0%	0.0%	0.0%	0.1%
k__Bacteria;p__Nitrospirae;c__Nitrospira;o__Nitrospirales;f__[Thermodesulfovibrionaceae];g__GOUTA19	0.0%	0.0%	0.0%	0.0%	0.0%	0.0%	0.1%
k__Bacteria;p__Nitrospirae;c__Nitrospira;o__Nitrospirales;f__[Thermodesulfovibrionaceae];g__HB118	0.0%	0.0%	0.0%	0.0%	0.0%	0.0%	0.0%
k__Bacteria;p__OD1;c__;o__;f__;g__	0.0%	0.1%	0.1%	0.0%	0.0%	0.0%	0.0%
k__Bacteria;p__OD1;c__ABY1;o__;f__;g__	0.1%	0.0%	0.0%	0.0%	0.2%	0.1%	0.0%
k__Bacteria;p__OD1;c__Mb-NB09;o__;f__;g__	0.0%	0.0%	0.0%	0.0%	0.0%	0.0%	0.0%
k__Bacteria;p__OD1;c__SM2F11;o__;f__;g__	0.2%	0.1%	0.2%	0.1%	0.2%	0.1%	0.2%
k__Bacteria;p__OD1;c__ZB2;o__;f__;g__	0.1%	0.1%	0.1%	0.0%	0.0%	0.0%	0.0%
k__Bacteria;p__OP1;c__[Acetothermia];o__[Acetothermales];f__;g__	0.0%	0.0%	0.0%	0.0%	0.0%	0.0%	0.0%
k__Bacteria;p__OP11;c__;o__;f__;g__	0.0%	0.0%	0.0%	0.0%	0.0%	0.0%	0.0%
k__Bacteria;p__OP11;c__OP11-3;o__;f__;g__	0.0%	0.0%	0.0%	0.0%	0.0%	0.0%	0.0%
k__Bacteria;p__OP11;c__OP11-4;o__;f__;g__	0.0%	0.0%	0.0%	0.0%	0.1%	0.0%	0.0%
k__Bacteria;p__OP11;c__WCHB1-64;o__;f__;g__	0.0%	0.0%	0.0%	0.0%	0.0%	0.0%	0.0%
k__Bacteria;p__OP11;c__WCHB1-64;o__K2-4-19;f__;g__	0.0%	0.0%	0.0%	0.0%	0.0%	0.0%	0.0%
k__Bacteria;p__OP11;c__WCHB1-64;o__d153;f__;g__	0.0%	0.0%	0.0%	0.0%	0.0%	0.0%	0.0%
k__Bacteria;p__OP3;c__;o__;f__;g__	0.0%	0.0%	0.0%	0.0%	0.0%	0.0%	0.0%
k__Bacteria;p__OP3;c__PBS-25;o__;f__;g__	0.0%	0.0%	0.0%	0.0%	0.0%	0.0%	0.0%
k__Bacteria;p__OP3;c__koll11;o__;f__;g__	0.0%	0.0%	0.1%	0.0%	0.0%	0.1%	0.0%
k__Bacteria;p__OP3;c__koll11;o__GIF10;f__;g__	0.0%	0.0%	0.0%	0.0%	0.0%	0.0%	0.0%
k__Bacteria;p__OP3;c__koll11;o__GIF10;f__kpj58rc;g__	0.0%	0.0%	0.0%	0.0%	0.0%	0.0%	0.0%
k__Bacteria;p__Planctomycetes;c__;o__;f__;g__	0.0%	0.0%	0.0%	0.0%	0.0%	0.0%	0.0%
k__Bacteria;p__Planctomycetes;c__028H05-P-BN-P5;o__;f__;g__	0.0%	0.0%	0.0%	0.0%	0.0%	0.0%	0.0%
k__Bacteria;p__Planctomycetes;c__BD7-11;o__;f__;g__	0.0%	0.0%	0.1%	0.0%	0.0%	0.1%	0.0%
k__Bacteria;p__Planctomycetes;c__C6;o__MVS-107;f__;g__	0.0%	0.0%	0.0%	0.0%	0.0%	0.0%	0.0%
k__Bacteria;p__Planctomycetes;c__C6;o__Ucm1571;f__;g__	0.0%	0.0%	0.0%	0.0%	0.0%	0.0%	0.0%
k__Bacteria;p__Planctomycetes;c__C6;o__d113;f__;g__	0.0%	0.0%	0.0%	0.0%	0.0%	0.0%	0.0%
k__Bacteria;p__Planctomycetes;c__ODP123;o__T8-B82;f__;g__	0.0%	0.0%	0.0%	0.0%	0.0%	0.0%	0.0%
k__Bacteria;p__Planctomycetes;c__OM190;o__;f__;g__	0.0%	0.0%	0.0%	0.0%	0.0%	0.0%	0.0%
k__Bacteria;p__Planctomycetes;c__OM190;o__CL500-15;f__;g__	0.0%	0.1%	0.0%	0.0%	0.0%	0.0%	0.0%
k__Bacteria;p__Planctomycetes;c__OM190;o__agg27;f__;g__	0.1%	0.1%	0.1%	0.1%	0.0%	0.1%	0.1%
k__Bacteria;p__Planctomycetes;c__Phycisphaerae;o__AKAU3564;f__;g__	0.0%	0.0%	0.0%	0.0%	0.0%	0.0%	0.0%
k__Bacteria;p__Planctomycetes;c__Phycisphaerae;o__CCM11a;f__;g__	0.0%	0.0%	0.2%	0.0%	0.0%	0.0%	0.0%
k__Bacteria;p__Planctomycetes;c__Phycisphaerae;o__Phycisphaerales;f__;g__	0.3%	0.4%	0.3%	0.4%	0.0%	0.3%	0.2%
k__Bacteria;p__Planctomycetes;c__Phycisphaerae;o__Phycisphaerales;f__Phycisphaeraceae;g__	0.0%	0.0%	0.0%	0.0%	0.0%	0.0%	0.0%
k__Bacteria;p__Planctomycetes;c__Phycisphaerae;o__Pla1;f__;g__	0.0%	0.0%	0.0%	0.0%	0.0%	0.0%	0.0%
k__Bacteria;p__Planctomycetes;c__Phycisphaerae;o__S-70;f__;g__	0.0%	0.0%	0.0%	0.0%	0.0%	0.0%	0.0%
k__Bacteria;p__Planctomycetes;c__Phycisphaerae;o__WD2101;f__;g__	0.0%	0.0%	0.0%	0.0%	0.0%	0.0%	0.0%
k__Bacteria;p__Planctomycetes;c__Phycisphaerae;o__mle1-8;f__;g__	0.0%	0.0%	0.0%	0.0%	0.0%	0.0%	0.0%
k__Bacteria;p__Planctomycetes;c__Pla3;o__;f__;g__	0.0%	0.0%	0.0%	0.0%	0.0%	0.1%	0.0%
k__Bacteria;p__Planctomycetes;c__Pla4;o__;f__;g__	0.0%	0.0%	0.0%	0.0%	0.0%	0.0%	0.0%
k__Bacteria;p__Planctomycetes;c__Planctomycetia;o__;f__;g__	0.0%	0.0%	0.0%	0.0%	0.0%	0.0%	0.0%
k__Bacteria;p__Planctomycetes;c__Planctomycetia;o__B97;f__;g__	0.0%	0.0%	0.0%	0.1%	0.0%	0.0%	0.0%
k__Bacteria;p__Planctomycetes;c__Planctomycetia;o__Gemmatales;f__Gemmataceae;Other	0.0%	0.0%	0.0%	0.0%	0.0%	0.0%	0.0%
k__Bacteria;p__Planctomycetes;c__Planctomycetia;o__Gemmatales;f__Gemmataceae;g__	1.0%	0.2%	0.6%	0.9%	2.8%	0.7%	0.8%
k__Bacteria;p__Planctomycetes;c__Planctomycetia;o__Gemmatales;f__Gemmataceae;g__Gemmata	0.1%	0.1%	0.1%	0.2%	0.0%	0.0%	0.1%
k__Bacteria;p__Planctomycetes;c__Planctomycetia;o__Gemmatales;f__Isosphaeraceae;g__	0.0%	0.0%	0.0%	0.1%	0.0%	0.0%	0.0%
k__Bacteria;p__Planctomycetes;c__Planctomycetia;o__Gemmatales;f__Isosphaeraceae;g__Isosphaera	0.0%	0.0%	0.0%	0.0%	0.0%	0.0%	0.0%
k__Bacteria;p__Planctomycetes;c__Planctomycetia;o__Pirellulales;f__Pirellulaceae;Other	0.0%	0.0%	0.0%	0.0%	0.0%	0.0%	0.0%
k__Bacteria;p__Planctomycetes;c__Planctomycetia;o__Pirellulales;f__Pirellulaceae;g__	0.1%	0.2%	0.1%	0.2%	0.0%	0.1%	0.2%
k__Bacteria;p__Planctomycetes;c__Planctomycetia;o__Pirellulales;f__Pirellulaceae;g__A17	0.0%	0.0%	0.0%	0.0%	0.0%	0.0%	0.0%
k__Bacteria;p__Planctomycetes;c__Planctomycetia;o__Pirellulales;f__Pirellulaceae;g__Pirellula	0.0%	0.0%	0.0%	0.1%	0.0%	0.0%	0.1%
k__Bacteria;p__Planctomycetes;c__Planctomycetia;o__Planctomycetales;f__Planctomycetaceae;g__Planctomyces	0.0%	0.1%	0.0%	0.1%	0.0%	0.0%	0.0%
k__Bacteria;p__Planctomycetes;c__[Brocadiae];o__Brocadiales;f__Brocadiaceae;g__Candidatus Brocadia	0.0%	0.0%	0.0%	0.0%	0.0%	0.0%	0.0%
k__Bacteria;p__Planctomycetes;c__vadinHA49;o__DH61;f__;g__	0.0%	0.0%	0.0%	0.0%	0.0%	0.0%	0.0%
k__Bacteria;p__Planctomycetes;c__vadinHA49;o__PHOS-HE93;f__;g__	0.0%	0.0%	0.0%	0.0%	0.0%	0.0%	0.0%
k__Bacteria;p__Planctomycetes;c__vadinHA49;o__p04_C01;f__;g__	0.0%	0.0%	0.0%	0.0%	0.0%	0.0%	0.0%
k__Bacteria;p__Proteobacteria;Other;Other;Other;Other	0.0%	0.0%	0.0%	0.0%	0.0%	0.0%	0.0%
k__Bacteria;p__Proteobacteria;c__;o__;f__;g__	0.0%	0.0%	0.0%	0.0%	0.0%	0.0%	0.0%
k__Bacteria;p__Proteobacteria;c__Alphaproteobacteria;Other;Other;Other	0.0%	0.0%	0.0%	0.0%	0.0%	0.0%	0.0%
k__Bacteria;p__Proteobacteria;c__Alphaproteobacteria;o__;f__;g__	0.4%	1.1%	0.5%	0.4%	0.0%	0.1%	0.0%
k__Bacteria;p__Proteobacteria;c__Alphaproteobacteria;o__BD7-3;f__;g__	0.1%	0.2%	0.1%	0.1%	0.0%	0.0%	0.0%

	k__Bacteria;p__Proteobacteria;c__Alphaproteobacteria;o__Caulobacterales;Other;Other	0.0%	0.0%	0.0%	0.0%	0.0%	0.0%	0.0%
	k__Bacteria;p__Proteobacteria;c__Alphaproteobacteria;o__Caulobacterales;f__;g__	0.0%	0.0%	0.0%	0.0%	0.0%	0.0%	0.0%
	k__Bacteria;p__Proteobacteria;c__Alphaproteobacteria;o__Caulobacterales;f__Caulobacteraceae;Other	0.0%	0.1%	0.0%	0.0%	0.0%	0.0%	0.0%
	k__Bacteria;p__Proteobacteria;c__Alphaproteobacteria;o__Caulobacterales;f__Caulobacteraceae;g__	0.1%	0.2%	0.1%	0.2%	0.2%	0.0%	0.0%
	k__Bacteria;p__Proteobacteria;c__Alphaproteobacteria;o__Caulobacterales;f__Caulobacteraceae;g__Arthrospira	0.0%	0.0%	0.0%	0.0%	0.0%	0.0%	0.0%
	k__Bacteria;p__Proteobacteria;c__Alphaproteobacteria;o__Caulobacterales;f__Caulobacteraceae;g__Asticcacaulis	0.0%	0.0%	0.0%	0.0%	0.0%	0.0%	0.0%
	k__Bacteria;p__Proteobacteria;c__Alphaproteobacteria;o__Caulobacterales;f__Caulobacteraceae;g__Brevundimonas	0.0%	0.0%	0.0%	0.0%	0.0%	0.0%	0.0%
	k__Bacteria;p__Proteobacteria;c__Alphaproteobacteria;o__Caulobacterales;f__Caulobacteraceae;g__Caulobacter	0.0%	0.0%	0.0%	0.0%	0.0%	0.0%	0.0%
	k__Bacteria;p__Proteobacteria;c__Alphaproteobacteria;o__Caulobacterales;f__Caulobacteraceae;g__Mycoplana	0.0%	0.1%	0.0%	0.0%	0.0%	0.0%	0.0%
	k__Bacteria;p__Proteobacteria;c__Alphaproteobacteria;o__Caulobacterales;f__Caulobacteraceae;g__Phenylobacterium	0.0%	0.1%	0.0%	0.1%	0.0%	0.0%	0.0%
	k__Bacteria;p__Proteobacteria;c__Alphaproteobacteria;o__Ellin329;f__;g__	0.4%	0.5%	0.2%	1.1%	0.5%	0.0%	0.3%
	k__Bacteria;p__Proteobacteria;c__Alphaproteobacteria;o__Kiloniellales;f__;g__	0.0%	0.0%	0.0%	0.0%	0.0%	0.0%	0.0%
	k__Bacteria;p__Proteobacteria;c__Alphaproteobacteria;o__Rhizobiales;Other;Other	0.1%	0.3%	0.1%	0.2%	0.0%	0.1%	0.1%
	k__Bacteria;p__Proteobacteria;c__Alphaproteobacteria;o__Rhizobiales;f__;g__	1.0%	1.0%	1.2%	1.4%	0.0%	1.6%	0.9%
	k__Bacteria;p__Proteobacteria;c__Alphaproteobacteria;o__Rhizobiales;f__Aurantimonadaceae;Other	0.0%	0.0%	0.0%	0.0%	0.0%	0.0%	0.0%
	k__Bacteria;p__Proteobacteria;c__Alphaproteobacteria;o__Rhizobiales;f__Aurantimonadaceae;g__	0.0%	0.0%	0.0%	0.0%	0.0%	0.0%	0.0%
	k__Bacteria;p__Proteobacteria;c__Alphaproteobacteria;o__Rhizobiales;f__Bartonellaceae;g__Bartonella	0.0%	0.0%	0.0%	0.0%	0.0%	0.0%	0.0%
	k__Bacteria;p__Proteobacteria;c__Alphaproteobacteria;o__Rhizobiales;f__Beijerinckiaceae;Other	0.0%	0.0%	0.0%	0.0%	0.0%	0.0%	0.0%
	k__Bacteria;p__Proteobacteria;c__Alphaproteobacteria;o__Rhizobiales;f__Beijerinckiaceae;g__	0.1%	0.0%	0.0%	0.1%	0.0%	0.6%	0.1%
	k__Bacteria;p__Proteobacteria;c__Alphaproteobacteria;o__Rhizobiales;f__Beijerinckiaceae;g__Beijerinckia	0.0%	0.0%	0.0%	0.0%	0.0%	0.0%	0.0%
	k__Bacteria;p__Proteobacteria;c__Alphaproteobacteria;o__Rhizobiales;f__Beijerinckiaceae;g__Chelatococcus	0.0%	0.0%	0.0%	0.0%	0.0%	0.0%	0.0%
	k__Bacteria;p__Proteobacteria;c__Alphaproteobacteria;o__Rhizobiales;f__Bradyrhizobiaceae;Other	0.1%	0.0%	0.0%	0.3%	0.0%	0.0%	0.1%
	k__Bacteria;p__Proteobacteria;c__Alphaproteobacteria;o__Rhizobiales;f__Bradyrhizobiaceae;g__	0.4%	0.3%	0.1%	1.1%	0.2%	0.0%	0.3%
	k__Bacteria;p__Proteobacteria;c__Alphaproteobacteria;o__Rhizobiales;f__Bradyrhizobiaceae;g__Balneimonas	0.1%	0.0%	0.0%	0.2%	0.0%	0.0%	0.2%
	k__Bacteria;p__Proteobacteria;c__Alphaproteobacteria;o__Rhizobiales;f__Bradyrhizobiaceae;g__Bosea	0.0%	0.0%	0.0%	0.0%	0.0%	0.0%	0.0%
	k__Bacteria;p__Proteobacteria;c__Alphaproteobacteria;o__Rhizobiales;f__Bradyrhizobiaceae;g__Bradyrhizobium	0.0%	0.0%	0.0%	0.0%	0.0%	0.0%	0.0%
	k__Bacteria;p__Proteobacteria;c__Alphaproteobacteria;o__Rhizobiales;f__Brucellaceae;Other	0.0%	0.0%	0.0%	0.0%	0.0%	0.0%	0.0%
	k__Bacteria;p__Proteobacteria;c__Alphaproteobacteria;o__Rhizobiales;f__Brucellaceae;g__Ochrobactrum	0.0%	0.0%	0.0%	0.0%	0.0%	0.0%	0.0%
	k__Bacteria;p__Proteobacteria;c__Alphaproteobacteria;o__Rhizobiales;f__Hyphomicrobiaceae;Other	0.0%	0.0%	0.0%	0.0%	0.0%	0.0%	0.1%
	k__Bacteria;p__Proteobacteria;c__Alphaproteobacteria;o__Rhizobiales;f__Hyphomicrobiaceae;g__	1.0%	1.3%	0.2%	1.5%	0.1%	0.6%	2.4%
	k__Bacteria;p__Proteobacteria;c__Alphaproteobacteria;o__Rhizobiales;f__Hyphomicrobiaceae;g__Devosia	0.2%	0.6%	0.1%	0.3%	0.0%	0.1%	0.0%
	k__Bacteria;p__Proteobacteria;c__Alphaproteobacteria;o__Rhizobiales;f__Hyphomicrobiaceae;g__Hyphomicrobium	0.2%	0.3%	0.1%	0.6%	0.0%	0.1%	0.2%
	k__Bacteria;p__Proteobacteria;c__Alphaproteobacteria;o__Rhizobiales;f__Hyphomicrobiaceae;g__Parvibaculum	0.0%	0.0%	0.0%	0.0%	0.0%	0.0%	0.0%
	k__Bacteria;p__Proteobacteria;c__Alphaproteobacteria;o__Rhizobiales;f__Hyphomicrobiaceae;g__Pedomicrobium	0.3%	0.8%	0.1%	0.8%	0.0%	0.2%	0.1%
	k__Bacteria;p__Proteobacteria;c__Alphaproteobacteria;o__Rhizobiales;f__Hyphomicrobiaceae;g__Rhodoplanes	2.5%	2.4%	0.4%	5.0%	1.1%	0.7%	5.7%
	k__Bacteria;p__Proteobacteria;c__Alphaproteobacteria;o__Rhizobiales;f__Methylobacteriaceae;g__	0.0%	0.0%	0.0%	0.0%	0.0%	0.0%	0.0%
	k__Bacteria;p__Proteobacteria;c__Alphaproteobacteria;o__Rhizobiales;f__Methylobacteriaceae;g__Methylobacterium	0.0%	0.0%	0.0%	0.0%	0.0%	0.0%	0.0%
	k__Bacteria;p__Proteobacteria;c__Alphaproteobacteria;o__Rhizobiales;f__Methylocystaceae;Other	0.0%	0.0%	0.0%	0.0%	0.0%	0.0%	0.0%
	k__Bacteria;p__Proteobacteria;c__Alphaproteobacteria;o__Rhizobiales;f__Methylocystaceae;g__	0.1%	0.1%	0.0%	0.1%	0.3%	0.0%	0.0%
	k__Bacteria;p__Proteobacteria;c__Alphaproteobacteria;o__Rhizobiales;f__Methylocystaceae;g__Methylopila	0.0%	0.0%	0.0%	0.0%	0.0%	0.0%	0.0%
	k__Bacteria;p__Proteobacteria;c__Alphaproteobacteria;o__Rhizobiales;f__Methylocystaceae;g__Methylosinus	0.0%	0.0%	0.0%	0.0%	0.0%	0.0%	0.0%
	k__Bacteria;p__Proteobacteria;c__Alphaproteobacteria;o__Rhizobiales;f__Methylocystaceae;g__Pleomorphomonas	0.0%	0.0%	0.0%	0.0%	0.0%	0.0%	0.0%
	k__Bacteria;p__Proteobacteria;c__Alphaproteobacteria;o__Rhizobiales;f__Phyllobacteriaceae;Other	0.0%	0.1%	0.0%	0.1%	0.0%	0.0%	0.0%
	k__Bacteria;p__Proteobacteria;c__Alphaproteobacteria;o__Rhizobiales;f__Phyllobacteriaceae;g__	0.1%	0.2%	0.0%	0.0%	0.0%	0.0%	0.0%
	k__Bacteria;p__Proteobacteria;c__Alphaproteobacteria;o__Rhizobiales;f__Phyllobacteriaceae;g__Aminobacter	0.0%	0.0%	0.0%	0.0%	0.0%	0.0%	0.0%
	k__Bacteria;p__Proteobacteria;c__Alphaproteobacteria;o__Rhizobiales;f__Phyllobacteriaceae;g__Chelativorans	0.0%	0.0%	0.0%	0.0%	0.0%	0.0%	0.0%
	k__Bacteria;p__Proteobacteria;c__Alphaproteobacteria;o__Rhizobiales;f__Phyllobacteriaceae;g__Mesorhizobium	0.0%	0.0%	0.0%	0.1%	0.0%	0.0%	0.0%
	k__Bacteria;p__Proteobacteria;c__Alphaproteobacteria;o__Rhizobiales;f__Phyllobacteriaceae;g__Phyllobacterium	0.0%	0.0%	0.0%	0.0%	0.0%	0.0%	0.0%
	k__Bacteria;p__Proteobacteria;c__Alphaproteobacteria;o__Rhizobiales;f__Rhizobiaceae;Other	0.0%	0.0%	0.0%	0.1%	0.0%	0.0%	0.0%
	k__Bacteria;p__Proteobacteria;c__Alphaproteobacteria;o__Rhizobiales;f__Rhizobiaceae;g__	0.1%	0.1%	0.0%	0.1%	0.0%	0.0%	0.1%
	k__Bacteria;p__Proteobacteria;c__Alphaproteobacteria;o__Rhizobiales;f__Rhizobiaceae;g__Agrobacterium	0.0%	0.1%	0.0%	0.1%	0.0%	0.0%	0.0%
	k__Bacteria;p__Proteobacteria;c__Alphaproteobacteria;o__Rhizobiales;f__Rhizobiaceae;g__Kaistia	0.0%	0.0%	0.0%	0.0%	0.0%	0.0%	0.0%
	k__Bacteria;p__Proteobacteria;c__Alphaproteobacteria;o__Rhizobiales;f__Rhizobiaceae;g__Rhizobium	0.0%	0.0%	0.0%	0.0%	0.0%	0.0%	0.0%
	k__Bacteria;p__Proteobacteria;c__Alphaproteobacteria;o__Rhizobiales;f__Rhodobiaceae;Other	0.0%	0.0%	0.0%	0.0%	0.0%	0.0%	0.0%
	k__Bacteria;p__Proteobacteria;c__Alphaproteobacteria;o__Rhizobiales;f__Rhodobiaceae;g__	0.0%	0.0%	0.0%	0.0%	0.0%	0.0%	0.0%
	k__Bacteria;p__Proteobacteria;c__Alphaproteobacteria;o__Rhizobiales;f__Rhodobiaceae;g__Afifella	0.2%	0.4%	0.1%	0.2%	0.0%	0.1%	0.2%
	k__Bacteria;p__Proteobacteria;c__Alphaproteobacteria;o__Rhizobiales;f__Xanthobacteraceae;g__	0.0%	0.0%	0.0%	0.0%	0.0%	0.0%	0.0%
	k__Bacteria;p__Proteobacteria;c__Alphaproteobacteria;o__Rhizobiales;f__Xanthobacteraceae;g__Labrys	0.0%	0.0%	0.0%	0.1%	0.0%	0.0%	0.0%
	k__Bacteria;p__Proteobacteria;c__Alphaproteobacteria;o__Rhizobiales;f__Xanthobacteraceae;g__Xanthobacter	0.0%	0.0%	0.0%	0.0%	0.0%	0.0%	0.0%
	k__Bacteria;p__Proteobacteria;c__Alphaproteobacteria;o__Rhodobacterales;f__Hyphomonadaceae;g__	0.3%	0.8%	0.4%	0.5%	0.0%	0.1%	0.0%
	k__Bacteria;p__Proteobacteria;c__Alphaproteobacteria;o__Rhodobacterales;f__Rhodobacteraceae;Other	0.0%	0.0%	0.0%	0.0%	0.0%	0.0%	0.0%
	k__Bacteria;p__Proteobacteria;c__Alphaproteobacteria;o__Rhodobacterales;f__Rhodobacteraceae;g__	0.1%	0.5%	0.1%	0.1%	0.0%	0.0%	0.1%
	k__Bacteria;p__Proteobacteria;c__Alphaproteobacteria;o__Rhodobacterales;f__Rhodobacteraceae;g__Amaricoccus	0.0%	0.1%	0.0%	0.0%	0.0%	0.0%	0.0%
	k__Bacteria;p__Proteobacteria;c__Alphaproteobacteria;o__Rhodobacterales;f__Rhodobacteraceae;g__Anaerospora	0.0%	0.0%	0.0%	0.0%	0.0%	0.0%	0.0%
	k__Bacteria;p__Proteobacteria;c__Alphaproteobacteria;o__Rhodobacterales;f__Rhodobacteraceae;g__Paracoccus	0.0%	0.0%	0.0%	0.0%	0.0%	0.0%	0.0%
	k__Bacteria;p__Proteobacteria;c__Alphaproteobacteria;o__Rhodobacterales;f__Rhodobacteraceae;g__Rhodobacter	0.0%	0.0%	0.1%	0.0%	0.0%	0.0%	0.0%
	k__Bacteria;p__Proteobacteria;c__Alphaproteobacteria;o__Rhodobacterales;f__Rhodobacteraceae;g__Rubellimicrobium	0.0%	0.0%	0.2%	0.0%	0.0%	0.0%	0.0%

	k__Bacteria;p__Proteobacteria;c__Alphaproteobacteria;o__Rhodospirillales;Other;Other	0.0%	0.0%	0.0%	0.0%	0.0%	0.0%	0.0%
	k__Bacteria;p__Proteobacteria;c__Alphaproteobacteria;o__Rhodospirillales;f__;g__	0.3%	1.3%	0.0%	0.1%	0.0%	0.2%	0.2%
	k__Bacteria;p__Proteobacteria;c__Alphaproteobacteria;o__Rhodospirillales;f__Acetobacteraceae;Other	0.0%	0.0%	0.0%	0.0%	0.0%	0.0%	0.0%
	k__Bacteria;p__Proteobacteria;c__Alphaproteobacteria;o__Rhodospirillales;f__Acetobacteraceae;g__	0.1%	0.0%	0.0%	0.3%	0.4%	0.0%	0.0%
	k__Bacteria;p__Proteobacteria;c__Alphaproteobacteria;o__Rhodospirillales;f__Acetobacteraceae;g__Roseococcus	0.0%	0.0%	0.0%	0.0%	0.0%	0.0%	0.0%
	k__Bacteria;p__Proteobacteria;c__Alphaproteobacteria;o__Rhodospirillales;f__Rhodospirillaceae;Other	0.0%	0.0%	0.0%	0.0%	0.0%	0.0%	0.0%
	k__Bacteria;p__Proteobacteria;c__Alphaproteobacteria;o__Rhodospirillales;f__Rhodospirillaceae;g__	2.2%	3.1%	0.7%	2.5%	4.0%	0.8%	2.3%
	k__Bacteria;p__Proteobacteria;c__Alphaproteobacteria;o__Rhodospirillales;f__Rhodospirillaceae;g__Azospirillum	0.0%	0.0%	0.0%	0.0%	0.0%	0.0%	0.0%
	k__Bacteria;p__Proteobacteria;c__Alphaproteobacteria;o__Rhodospirillales;f__Rhodospirillaceae;g__Inquilinus	0.0%	0.0%	0.0%	0.0%	0.0%	0.0%	0.0%
	k__Bacteria;p__Proteobacteria;c__Alphaproteobacteria;o__Rhodospirillales;f__Rhodospirillaceae;g__Magnetospirillum	0.0%	0.0%	0.0%	0.0%	0.0%	0.0%	0.0%
	k__Bacteria;p__Proteobacteria;c__Alphaproteobacteria;o__Rhodospirillales;f__Rhodospirillaceae;g__Phaeospirillum	0.0%	0.0%	0.0%	0.0%	0.0%	0.0%	0.0%
	k__Bacteria;p__Proteobacteria;c__Alphaproteobacteria;o__Rhodospirillales;f__Rhodospirillaceae;g__Rhodovibrio	0.0%	0.0%	0.0%	0.0%	0.0%	0.0%	0.0%
	k__Bacteria;p__Proteobacteria;c__Alphaproteobacteria;o__Rhodospirillales;f__Rhodospirillaceae;g__Skermanella	0.0%	0.0%	0.0%	0.0%	0.0%	0.0%	0.0%
	k__Bacteria;p__Proteobacteria;c__Alphaproteobacteria;o__Rhodospirillales;f__Rhodospirillaceae;g__Telmatospirillum	0.0%	0.0%	0.0%	0.0%	0.0%	0.0%	0.0%
	k__Bacteria;p__Proteobacteria;c__Alphaproteobacteria;o__Rickettsiales;f__;g__	0.1%	0.0%	0.1%	0.1%	0.1%	0.0%	0.1%
	k__Bacteria;p__Proteobacteria;c__Alphaproteobacteria;o__Rickettsiales;f__Holosporaceae;g__	0.0%	0.0%	0.0%	0.0%	0.0%	0.0%	0.0%
	k__Bacteria;p__Proteobacteria;c__Alphaproteobacteria;o__Rickettsiales;f__Rickettsiaceae;g__	0.0%	0.0%	0.0%	0.0%	0.0%	0.0%	0.0%
	k__Bacteria;p__Proteobacteria;c__Alphaproteobacteria;o__Rickettsiales;f__Rickettsiaceae;g__Orientia	0.0%	0.0%	0.0%	0.0%	0.0%	0.0%	0.0%
	k__Bacteria;p__Proteobacteria;c__Alphaproteobacteria;o__Rickettsiales;f__Rickettsiaceae;g__Rickettsia	0.0%	0.0%	0.0%	0.0%	0.0%	0.0%	0.0%
	k__Bacteria;p__Proteobacteria;c__Alphaproteobacteria;o__Rickettsiales;f__mitochondria;Other	0.0%	0.0%	0.0%	0.0%	0.0%	0.0%	0.0%
	k__Bacteria;p__Proteobacteria;c__Alphaproteobacteria;o__Rickettsiales;f__mitochondria;g__	0.0%	0.0%	0.0%	0.0%	0.0%	0.0%	0.0%
	k__Bacteria;p__Proteobacteria;c__Alphaproteobacteria;o__Sphingomonadales;Other;Other	0.0%	0.0%	0.0%	0.0%	0.0%	0.0%	0.0%
	k__Bacteria;p__Proteobacteria;c__Alphaproteobacteria;o__Sphingomonadales;f__;g__	0.0%	0.0%	0.1%	0.0%	0.0%	0.0%	0.0%
	k__Bacteria;p__Proteobacteria;c__Alphaproteobacteria;o__Sphingomonadales;f__Erythrobacteraceae;Other	0.0%	0.0%	0.0%	0.0%	0.0%	0.0%	0.0%
	k__Bacteria;p__Proteobacteria;c__Alphaproteobacteria;o__Sphingomonadales;f__Erythrobacteraceae;g__	0.1%	0.2%	0.2%	0.2%	0.0%	0.0%	0.1%
	k__Bacteria;p__Proteobacteria;c__Alphaproteobacteria;o__Sphingomonadales;f__Erythrobacteraceae;g__Lutibacterium	0.0%	0.0%	0.0%	0.0%	0.0%	0.0%	0.0%
	k__Bacteria;p__Proteobacteria;c__Alphaproteobacteria;o__Sphingomonadales;f__Sphingomonadaceae;Other	0.0%	0.0%	0.1%	0.0%	0.0%	0.0%	0.0%
	k__Bacteria;p__Proteobacteria;c__Alphaproteobacteria;o__Sphingomonadales;f__Sphingomonadaceae;g__	0.4%	0.7%	0.5%	0.5%	0.0%	0.3%	0.1%
	k__Bacteria;p__Proteobacteria;c__Alphaproteobacteria;o__Sphingomonadales;f__Sphingomonadaceae;g__Blastomonas	0.0%	0.0%	0.0%	0.0%	0.0%	0.0%	0.0%
	k__Bacteria;p__Proteobacteria;c__Alphaproteobacteria;o__Sphingomonadales;f__Sphingomonadaceae;g__Kaistobacter	1.4%	0.9%	5.8%	0.8%	0.0%	0.6%	0.5%
	k__Bacteria;p__Proteobacteria;c__Alphaproteobacteria;o__Sphingomonadales;f__Sphingomonadaceae;g__Novosphingobium	0.1%	0.0%	0.2%	0.2%	0.0%	0.0%	0.0%
	k__Bacteria;p__Proteobacteria;c__Alphaproteobacteria;o__Sphingomonadales;f__Sphingomonadaceae;g__Sphingobium	0.0%	0.0%	0.0%	0.1%	0.0%	0.0%	0.0%
	k__Bacteria;p__Proteobacteria;c__Alphaproteobacteria;o__Sphingomonadales;f__Sphingomonadaceae;g__Sphingomonas	0.1%	0.1%	0.4%	0.2%	0.0%	0.0%	0.0%
	k__Bacteria;p__Proteobacteria;c__Alphaproteobacteria;o__Sphingomonadales;f__Sphingomonadaceae;g__Sphingopyxis	0.0%	0.0%	0.0%	0.0%	0.0%	0.0%	0.0%
	k__Bacteria;p__Proteobacteria;c__Betaproteobacteria;Other;Other;Other	0.0%	0.0%	0.0%	0.0%	0.2%	0.0%	0.0%
	k__Bacteria;p__Proteobacteria;c__Betaproteobacteria;o__;f__;g__	1.4%	1.2%	1.3%	1.8%	0.0%	1.8%	2.3%
	k__Bacteria;p__Proteobacteria;c__Betaproteobacteria;o__A21b;f__EB1003;g__	0.0%	0.0%	0.0%	0.0%	0.0%	0.0%	0.0%
	k__Bacteria;p__Proteobacteria;c__Betaproteobacteria;o__A21b;f__UD5;g__	0.0%	0.0%	0.1%	0.0%	0.0%	0.0%	0.0%
	k__Bacteria;p__Proteobacteria;c__Betaproteobacteria;o__Burkholderiales;Other;Other	0.0%	0.0%	0.0%	0.0%	0.0%	0.0%	0.0%
	k__Bacteria;p__Proteobacteria;c__Betaproteobacteria;o__Burkholderiales;f__;g__	0.0%	0.0%	0.0%	0.0%	0.0%	0.0%	0.0%
	k__Bacteria;p__Proteobacteria;c__Betaproteobacteria;o__Burkholderiales;f__Alcaligenaceae;Other	0.0%	0.0%	0.0%	0.0%	0.0%	0.0%	0.0%
	k__Bacteria;p__Proteobacteria;c__Betaproteobacteria;o__Burkholderiales;f__Alcaligenaceae;g__	0.2%	0.1%	0.4%	0.1%	0.0%	0.1%	0.6%
	k__Bacteria;p__Proteobacteria;c__Betaproteobacteria;o__Burkholderiales;f__Alcaligenaceae;g__Achromobacter	0.0%	0.0%	0.0%	0.0%	0.0%	0.0%	0.0%
	k__Bacteria;p__Proteobacteria;c__Betaproteobacteria;o__Burkholderiales;f__Alcaligenaceae;g__Tetrathiobacter	0.0%	0.0%	0.0%	0.0%	0.0%	0.0%	0.0%
	k__Bacteria;p__Proteobacteria;c__Betaproteobacteria;o__Burkholderiales;f__Burkholderiaceae;Other	0.0%	0.0%	0.0%	0.0%	0.0%	0.0%	0.0%
	k__Bacteria;p__Proteobacteria;c__Betaproteobacteria;o__Burkholderiales;f__Burkholderiaceae;g__	0.0%	0.0%	0.0%	0.0%	0.0%	0.0%	0.0%
	k__Bacteria;p__Proteobacteria;c__Betaproteobacteria;o__Burkholderiales;f__Burkholderiaceae;g__Burkholderia	0.0%	0.0%	0.0%	0.0%	0.0%	0.0%	0.0%
	k__Bacteria;p__Proteobacteria;c__Betaproteobacteria;o__Burkholderiales;f__Burkholderiaceae;g__Lautropia	0.0%	0.0%	0.0%	0.0%	0.0%	0.0%	0.0%
	k__Bacteria;p__Proteobacteria;c__Betaproteobacteria;o__Burkholderiales;f__Burkholderiaceae;g__Pandoraea	0.0%	0.0%	0.0%	0.0%	0.0%	0.0%	0.0%
	k__Bacteria;p__Proteobacteria;c__Betaproteobacteria;o__Burkholderiales;f__Comamonadaceae;Other	0.1%	0.1%	0.1%	0.2%	0.0%	0.1%	0.1%
	k__Bacteria;p__Proteobacteria;c__Betaproteobacteria;o__Burkholderiales;f__Comamonadaceae;g__	0.3%	0.2%	0.5%	0.5%	0.1%	0.1%	0.1%
	k__Bacteria;p__Proteobacteria;c__Betaproteobacteria;o__Burkholderiales;f__Comamonadaceae;g__Acidovorax	0.0%	0.0%	0.0%	0.0%	0.0%	0.0%	0.0%
	k__Bacteria;p__Proteobacteria;c__Betaproteobacteria;o__Burkholderiales;f__Comamonadaceae;g__Azohydromonas	0.0%	0.0%	0.0%	0.0%	0.0%	0.0%	0.0%
	k__Bacteria;p__Proteobacteria;c__Betaproteobacteria;o__Burkholderiales;f__Comamonadaceae;g__Comamonas	0.0%	0.0%	0.0%	0.0%	0.0%	0.0%	0.0%
	k__Bacteria;p__Proteobacteria;c__Betaproteobacteria;o__Burkholderiales;f__Comamonadaceae;g__Delftia	0.0%	0.0%	0.0%	0.0%	0.0%	0.0%	0.0%
	k__Bacteria;p__Proteobacteria;c__Betaproteobacteria;o__Burkholderiales;f__Comamonadaceae;g__Hydrogenophaga	0.0%	0.0%	0.0%	0.0%	0.0%	0.0%	0.0%
	k__Bacteria;p__Proteobacteria;c__Betaproteobacteria;o__Burkholderiales;f__Comamonadaceae;g__Hylemonella	0.0%	0.0%	0.0%	0.0%	0.0%	0.0%	0.0%
	k__Bacteria;p__Proteobacteria;c__Betaproteobacteria;o__Burkholderiales;f__Comamonadaceae;g__Leptothrix	0.0%	0.0%	0.0%	0.0%	0.0%	0.0%	0.0%
	k__Bacteria;p__Proteobacteria;c__Betaproteobacteria;o__Burkholderiales;f__Comamonadaceae;g__Limnobacter	0.0%	0.0%	0.0%	0.0%	0.0%	0.0%	0.0%
	k__Bacteria;p__Proteobacteria;c__Betaproteobacteria;o__Burkholderiales;f__Comamonadaceae;g__Limnohabitans	0.0%	0.0%	0.0%	0.0%	0.0%	0.0%	0.0%
	k__Bacteria;p__Proteobacteria;c__Betaproteobacteria;o__Burkholderiales;f__Comamonadaceae;g__Methylibium	0.0%	0.0%	0.1%	0.1%	0.0%	0.0%	0.0%
	k__Bacteria;p__Proteobacteria;c__Betaproteobacteria;o__Burkholderiales;f__Comamonadaceae;g__Polaromonas	0.0%	0.0%	0.0%	0.0%	0.0%	0.0%	0.0%
	k__Bacteria;p__Proteobacteria;c__Betaproteobacteria;o__Burkholderiales;f__Comamonadaceae;g__Ramlibacter	0.1%	0.1%	0.2%	0.1%	0.0%	0.0%	0.0%
	k__Bacteria;p__Proteobacteria;c__Betaproteobacteria;o__Burkholderiales;f__Comamonadaceae;g__Rhodoferax	0.1%	0.0%	0.1%	0.0%	0.1%	0.1%	0.0%
	k__Bacteria;p__Proteobacteria;c__Betaproteobacteria;o__Burkholderiales;f__Comamonadaceae;g__Roseateles	0.0%	0.0%	0.0%	0.0%	0.0%	0.0%	0.0%
	k__Bacteria;p__Proteobacteria;c__Betaproteobacteria;o__Burkholderiales;f__Comamonadaceae;g__Rubrivivax	0.0%	0.0%	0.0%	0.0%	0.0%	0.0%	0.0%
	k__Bacteria;p__Proteobacteria;c__Betaproteobacteria;o__Burkholderiales;f__Comamonadaceae;g__Simplicispira	0.0%	0.0%	0.0%	0.0%	0.0%	0.0%	0.0%

	k__Bacteria;p__Proteobacteria;c__Betaproteobacteria;o__Burkholderiales;f__Comamonadaceae;g__Tepidimonas	0.0%	0.0%	0.0%	0.0%	0.0%	0.0%	0.0%
	k__Bacteria;p__Proteobacteria;c__Betaproteobacteria;o__Burkholderiales;f__Comamonadaceae;g__Variovorax	0.0%	0.0%	0.0%	0.0%	0.0%	0.0%	0.0%
	k__Bacteria;p__Proteobacteria;c__Betaproteobacteria;o__Burkholderiales;f__Oxalobacteraceae;Other	0.0%	0.0%	0.0%	0.0%	0.1%	0.0%	0.0%
	k__Bacteria;p__Proteobacteria;c__Betaproteobacteria;o__Burkholderiales;f__Oxalobacteraceae;g__	0.4%	0.1%	0.5%	0.6%	0.6%	0.3%	0.1%
	k__Bacteria;p__Proteobacteria;c__Betaproteobacteria;o__Burkholderiales;f__Oxalobacteraceae;g__Cupriavidus	0.0%	0.0%	0.0%	0.0%	0.0%	0.0%	0.0%
	k__Bacteria;p__Proteobacteria;c__Betaproteobacteria;o__Burkholderiales;f__Oxalobacteraceae;g__Janthinobacterium	0.2%	0.0%	0.4%	0.4%	0.0%	0.1%	0.0%
	k__Bacteria;p__Proteobacteria;c__Betaproteobacteria;o__Burkholderiales;f__Oxalobacteraceae;g__Oxalobacter	0.0%	0.0%	0.0%	0.0%	0.0%	0.0%	0.0%
	k__Bacteria;p__Proteobacteria;c__Betaproteobacteria;o__Burkholderiales;f__Oxalobacteraceae;g__Ralstonia	0.0%	0.0%	0.0%	0.0%	0.0%	0.0%	0.0%
	k__Bacteria;p__Proteobacteria;c__Betaproteobacteria;o__Ellin6067;f__;g__	0.3%	0.2%	0.4%	0.5%	0.0%	0.2%	0.2%
	k__Bacteria;p__Proteobacteria;c__Betaproteobacteria;o__Gallionellales;f__Gallionellaceae;g__Gallionella	0.0%	0.0%	0.0%	0.0%	0.0%	0.0%	0.1%
	k__Bacteria;p__Proteobacteria;c__Betaproteobacteria;o__Hydrogenophilales;f__Hydrogenophilaceae;g__Thiobacillus	0.0%	0.0%	0.0%	0.0%	0.0%	0.0%	0.0%
	k__Bacteria;p__Proteobacteria;c__Betaproteobacteria;o__IS-44;f__;g__	0.1%	0.2%	0.1%	0.4%	0.0%	0.1%	0.0%
	k__Bacteria;p__Proteobacteria;c__Betaproteobacteria;o__MND1;f__;g__	0.6%	0.4%	0.7%	0.3%	0.0%	0.5%	1.9%
	k__Bacteria;p__Proteobacteria;c__Betaproteobacteria;o__Methylophilales;Other;Other	0.0%	0.0%	0.0%	0.0%	0.0%	0.0%	0.0%
	k__Bacteria;p__Proteobacteria;c__Betaproteobacteria;o__Methylophilales;f__;g__	0.0%	0.0%	0.0%	0.0%	0.0%	0.0%	0.0%
	k__Bacteria;p__Proteobacteria;c__Betaproteobacteria;o__Methylophilales;f__Methylophilaceae;Other	0.0%	0.0%	0.0%	0.0%	0.0%	0.0%	0.0%
	k__Bacteria;p__Proteobacteria;c__Betaproteobacteria;o__Methylophilales;f__Methylophilaceae;g__	0.1%	0.0%	0.1%	0.0%	0.2%	0.0%	0.0%
	k__Bacteria;p__Proteobacteria;c__Betaproteobacteria;o__Methylophilales;f__Methylophilaceae;g__Methylotenera	0.0%	0.0%	0.1%	0.0%	0.0%	0.0%	0.0%
	k__Bacteria;p__Proteobacteria;c__Betaproteobacteria;o__Neisseriales;f__Neisseriaceae;Other	0.0%	0.0%	0.0%	0.0%	0.0%	0.0%	0.0%
	k__Bacteria;p__Proteobacteria;c__Betaproteobacteria;o__Neisseriales;f__Neisseriaceae;g__	0.0%	0.0%	0.0%	0.0%	0.2%	0.0%	0.0%
	k__Bacteria;p__Proteobacteria;c__Betaproteobacteria;o__Neisseriales;f__Neisseriaceae;g__Deefgea	0.0%	0.0%	0.0%	0.0%	0.0%	0.0%	0.0%
	k__Bacteria;p__Proteobacteria;c__Betaproteobacteria;o__Neisseriales;f__Neisseriaceae;g__Vogesella	0.0%	0.0%	0.0%	0.0%	0.0%	0.0%	0.0%
	k__Bacteria;p__Proteobacteria;c__Betaproteobacteria;o__Nitrosomonadales;Other;Other	0.0%	0.0%	0.0%	0.0%	0.0%	0.0%	0.0%
	k__Bacteria;p__Proteobacteria;c__Betaproteobacteria;o__Nitrosomonadales;f__;g__	0.3%	0.0%	0.1%	0.2%	1.9%	0.0%	0.0%
	k__Bacteria;p__Proteobacteria;c__Betaproteobacteria;o__Nitrosomonadales;f__Nitrosomonadaceae;Other	0.0%	0.0%	0.0%	0.0%	0.0%	0.0%	0.0%
	k__Bacteria;p__Proteobacteria;c__Betaproteobacteria;o__Nitrosomonadales;f__Nitrosomonadaceae;g__	0.5%	0.0%	0.5%	0.1%	2.0%	0.4%	0.0%
	k__Bacteria;p__Proteobacteria;c__Betaproteobacteria;o__Nitrosomonadales;f__Nitrosomonadaceae;g__Nitrosovibrio	0.2%	0.0%	0.1%	0.0%	0.9%	0.1%	0.0%
	k__Bacteria;p__Proteobacteria;c__Betaproteobacteria;o__Procabacteriales;f__Procabacteriaceae;g__	0.0%	0.0%	0.0%	0.0%	0.0%	0.0%	0.0%
	k__Bacteria;p__Proteobacteria;c__Betaproteobacteria;o__Procabacteriales;f__Procabacteriaceae;g__Procabacter	0.0%	0.0%	0.0%	0.0%	0.0%	0.0%	0.0%
	k__Bacteria;p__Proteobacteria;c__Betaproteobacteria;o__Rhodocyclales;f__Rhodocyclaceae;Other	0.0%	0.0%	0.0%	0.0%	0.0%	0.0%	0.0%
	k__Bacteria;p__Proteobacteria;c__Betaproteobacteria;o__Rhodocyclales;f__Rhodocyclaceae;g__	0.0%	0.0%	0.0%	0.2%	0.0%	0.0%	0.0%
	k__Bacteria;p__Proteobacteria;c__Betaproteobacteria;o__Rhodocyclales;f__Rhodocyclaceae;g__Azoarcus	0.0%	0.0%	0.0%	0.0%	0.0%	0.0%	0.0%
	k__Bacteria;p__Proteobacteria;c__Betaproteobacteria;o__Rhodocyclales;f__Rhodocyclaceae;g__Azospira	0.0%	0.0%	0.0%	0.0%	0.0%	0.0%	0.0%
	k__Bacteria;p__Proteobacteria;c__Betaproteobacteria;o__Rhodocyclales;f__Rhodocyclaceae;g__Dechloromonas	0.0%	0.0%	0.0%	0.0%	0.0%	0.0%	0.0%
	k__Bacteria;p__Proteobacteria;c__Betaproteobacteria;o__Rhodocyclales;f__Rhodocyclaceae;g__Dok59	0.0%	0.0%	0.0%	0.0%	0.0%	0.0%	0.0%
	k__Bacteria;p__Proteobacteria;c__Betaproteobacteria;o__Rhodocyclales;f__Rhodocyclaceae;g__Propionivibrio	0.0%	0.0%	0.0%	0.0%	0.0%	0.0%	0.0%
	k__Bacteria;p__Proteobacteria;c__Betaproteobacteria;o__Rhodocyclales;f__Rhodocyclaceae;g__Rhodocyclus	0.0%	0.0%	0.0%	0.0%	0.0%	0.0%	0.0%
	k__Bacteria;p__Proteobacteria;c__Betaproteobacteria;o__Rhodocyclales;f__Rhodocyclaceae;g__Sulfuritalea	0.0%	0.0%	0.0%	0.0%	0.0%	0.0%	0.0%
	k__Bacteria;p__Proteobacteria;c__Betaproteobacteria;o__Rhodocyclales;f__Rhodocyclaceae;g__Thauera	0.0%	0.0%	0.0%	0.0%	0.0%	0.0%	0.0%
	k__Bacteria;p__Proteobacteria;c__Betaproteobacteria;o__Rhodocyclales;f__Rhodocyclaceae;g__Uliginosibacterium	0.0%	0.0%	0.0%	0.0%	0.0%	0.0%	0.0%
	k__Bacteria;p__Proteobacteria;c__Betaproteobacteria;o__SBla14;f__;g__	0.0%	0.0%	0.0%	0.0%	0.0%	0.0%	0.0%
	k__Bacteria;p__Proteobacteria;c__Betaproteobacteria;o__SC-I-84;f__;g__	0.3%	0.1%	0.5%	0.4%	0.1%	0.2%	0.7%
	k__Bacteria;p__Proteobacteria;c__Betaproteobacteria;o__Thiobacterales;f__;g__	0.0%	0.0%	0.0%	0.0%	0.0%	0.0%	0.0%
	k__Bacteria;p__Proteobacteria;c__Deltaproteobacteria;Other;Other;Other	0.0%	0.0%	0.0%	0.0%	0.0%	0.0%	0.0%
	k__Bacteria;p__Proteobacteria;c__Deltaproteobacteria;o__;f__;g__	0.1%	0.0%	0.1%	0.1%	0.0%	0.1%	0.3%
	k__Bacteria;p__Proteobacteria;c__Deltaproteobacteria;o__BPC076;f__;g__	0.0%	0.0%	0.0%	0.0%	0.0%	0.0%	0.0%
	k__Bacteria;p__Proteobacteria;c__Deltaproteobacteria;o__Bdellovibrionales;f__Bacteriovoracaceae;Other	0.0%	0.0%	0.0%	0.0%	0.0%	0.0%	0.0%
	k__Bacteria;p__Proteobacteria;c__Deltaproteobacteria;o__Bdellovibrionales;f__Bacteriovoracaceae;g__	0.1%	0.1%	0.1%	0.2%	0.0%	0.0%	0.0%
	k__Bacteria;p__Proteobacteria;c__Deltaproteobacteria;o__Bdellovibrionales;f__Bacteriovoracaceae;g__Bacteriovorax	0.0%	0.0%	0.0%	0.0%	0.0%	0.0%	0.0%
	k__Bacteria;p__Proteobacteria;c__Deltaproteobacteria;o__Bdellovibrionales;f__Bacteriovoracaceae;g__Peredibacter	0.0%	0.0%	0.0%	0.0%	0.0%	0.0%	0.0%
	k__Bacteria;p__Proteobacteria;c__Deltaproteobacteria;o__Bdellovibrionales;f__Bdellovibrionaceae;g__	0.0%	0.0%	0.0%	0.0%	0.0%	0.0%	0.0%
	k__Bacteria;p__Proteobacteria;c__Deltaproteobacteria;o__Bdellovibrionales;f__Bdellovibrionaceae;g__Bdellovibrio	0.2%	0.3%	0.2%	0.4%	0.0%	0.1%	0.3%
	k__Bacteria;p__Proteobacteria;c__Deltaproteobacteria;o__Desulfarculales;f__Desulfarculaceae;g__	0.0%	0.0%	0.0%	0.0%	0.0%	0.0%	0.0%
	k__Bacteria;p__Proteobacteria;c__Deltaproteobacteria;o__Desulfobacterales;f__Desulfobacteraceae;g__Desulfococcus	0.0%	0.0%	0.0%	0.0%	0.0%	0.0%	0.0%
	k__Bacteria;p__Proteobacteria;c__Deltaproteobacteria;o__Desulfobacterales;f__Desulfobulbaceae;g__	0.0%	0.0%	0.0%	0.0%	0.0%	0.0%	0.1%
	k__Bacteria;p__Proteobacteria;c__Deltaproteobacteria;o__Desulfobacterales;f__Desulfobulbaceae;g__Desulfobulbus	0.0%	0.0%	0.0%	0.0%	0.0%	0.0%	0.0%
	k__Bacteria;p__Proteobacteria;c__Deltaproteobacteria;o__Desulfovibrionales;f__Desulfovibrionaceae;g__	0.0%	0.0%	0.0%	0.0%	0.0%	0.0%	0.0%
	k__Bacteria;p__Proteobacteria;c__Deltaproteobacteria;o__Desulfovibrionales;f__Desulfovibrionaceae;g__Desulfovibrio	0.0%	0.0%	0.0%	0.0%	0.0%	0.0%	0.0%
	k__Bacteria;p__Proteobacteria;c__Deltaproteobacteria;o__Desulfuromonadales;Other;Other	0.0%	0.0%	0.0%	0.0%	0.0%	0.0%	0.0%
	k__Bacteria;p__Proteobacteria;c__Deltaproteobacteria;o__Desulfuromonadales;f__Desulfuromonadaceae;g__	0.0%	0.0%	0.0%	0.0%	0.0%	0.0%	0.0%
	k__Bacteria;p__Proteobacteria;c__Deltaproteobacteria;o__Desulfuromonadales;f__Geobacteraceae;g__	0.0%	0.0%	0.0%	0.0%	0.0%	0.0%	0.0%
	k__Bacteria;p__Proteobacteria;c__Deltaproteobacteria;o__Desulfuromonadales;f__Geobacteraceae;g__Geobacter	0.1%	0.0%	0.0%	0.1%	0.0%	0.0%	0.6%
	k__Bacteria;p__Proteobacteria;c__Deltaproteobacteria;o__Desulfuromonadales;f__Pelobacteraceae;Other	0.0%	0.0%	0.0%	0.0%	0.0%	0.0%	0.0%
	k__Bacteria;p__Proteobacteria;c__Deltaproteobacteria;o__Desulfuromonadales;f__Pelobacteraceae;g__	0.0%	0.0%	0.0%	0.0%	0.0%	0.0%	0.1%
	k__Bacteria;p__Proteobacteria;c__Deltaproteobacteria;o__Desulfuromonadales;f__Pelobacteraceae;g__Pelobacter	0.0%	0.0%	0.0%	0.0%	0.0%	0.0%	0.0%
	k__Bacteria;p__Proteobacteria;c__Deltaproteobacteria;o__FAC87;f__;g__	0.0%	0.0%	0.0%	0.0%	0.0%	0.0%	0.0%
	k__Bacteria;p__Proteobacteria;c__Deltaproteobacteria;o__GMD14H09;f__;g__	0.0%	0.0%	0.0%	0.0%	0.0%	0.0%	0.0%

k__Bacteria;p__Proteobacteria;c__Deltaproteobacteria;o__MBNT15;f__;g__	0.0%	0.0%	0.0%	0.0%	0.0%	0.0%	0.1%
k__Bacteria;p__Proteobacteria;c__Deltaproteobacteria;o__MIZ46;f__;g__	0.1%	0.0%	0.1%	0.2%	0.0%	0.1%	0.1%
k__Bacteria;p__Proteobacteria;c__Deltaproteobacteria;o__Myxococcales;Other;Other	0.0%	0.0%	0.0%	0.0%	0.0%	0.0%	0.0%
k__Bacteria;p__Proteobacteria;c__Deltaproteobacteria;o__Myxococcales;f__;g__	1.0%	2.1%	0.7%	1.3%	0.3%	0.5%	1.0%
k__Bacteria;p__Proteobacteria;c__Deltaproteobacteria;o__Myxococcales;f__0319-6G20;g__	0.0%	0.0%	0.0%	0.0%	0.0%	0.0%	0.0%
k__Bacteria;p__Proteobacteria;c__Deltaproteobacteria;o__Myxococcales;f__Cystobacterineae;g__	0.0%	0.0%	0.0%	0.0%	0.0%	0.0%	0.0%
k__Bacteria;p__Proteobacteria;c__Deltaproteobacteria;o__Myxococcales;f__Haliangiaceae;Other	0.0%	0.0%	0.0%	0.0%	0.0%	0.0%	0.0%
k__Bacteria;p__Proteobacteria;c__Deltaproteobacteria;o__Myxococcales;f__Haliangiaceae;g__	0.2%	0.3%	0.2%	0.1%	0.0%	0.1%	0.2%
k__Bacteria;p__Proteobacteria;c__Deltaproteobacteria;o__Myxococcales;f__Haliangiaceae;g__Haliangium	0.0%	0.0%	0.0%	0.0%	0.0%	0.0%	0.0%
k__Bacteria;p__Proteobacteria;c__Deltaproteobacteria;o__Myxococcales;f__Myxococcaceae;Other	0.0%	0.0%	0.0%	0.0%	0.0%	0.0%	0.0%
k__Bacteria;p__Proteobacteria;c__Deltaproteobacteria;o__Myxococcales;f__Myxococcaceae;g__	0.0%	0.0%	0.0%	0.0%	0.0%	0.0%	0.0%
k__Bacteria;p__Proteobacteria;c__Deltaproteobacteria;o__Myxococcales;f__Myxococcaceae;g__Anaeromyxobacter	0.0%	0.0%	0.0%	0.0%	0.0%	0.0%	0.1%
k__Bacteria;p__Proteobacteria;c__Deltaproteobacteria;o__Myxococcales;f__Myxococcaceae;g__Myxococcus	0.0%	0.0%	0.0%	0.0%	0.0%	0.0%	0.0%
k__Bacteria;p__Proteobacteria;c__Deltaproteobacteria;o__Myxococcales;f__Nannocystaceae;g__Nannocystis	0.0%	0.0%	0.0%	0.0%	0.0%	0.0%	0.0%
k__Bacteria;p__Proteobacteria;c__Deltaproteobacteria;o__Myxococcales;f__Nannocystaceae;g__Plesiocystis	0.0%	0.1%	0.0%	0.0%	0.0%	0.0%	0.0%
k__Bacteria;p__Proteobacteria;c__Deltaproteobacteria;o__Myxococcales;f__OM27;g__	0.1%	0.2%	0.1%	0.1%	0.0%	0.1%	0.0%
k__Bacteria;p__Proteobacteria;c__Deltaproteobacteria;o__Myxococcales;f__Polyangiaceae;Other	0.0%	0.0%	0.0%	0.0%	0.0%	0.0%	0.0%
k__Bacteria;p__Proteobacteria;c__Deltaproteobacteria;o__Myxococcales;f__Polyangiaceae;g__	0.0%	0.0%	0.0%	0.0%	0.0%	0.0%	0.0%
k__Bacteria;p__Proteobacteria;c__Deltaproteobacteria;o__Myxococcales;f__Polyangiaceae;g__Chondromyces	0.0%	0.0%	0.0%	0.0%	0.0%	0.0%	0.0%
k__Bacteria;p__Proteobacteria;c__Deltaproteobacteria;o__NB1-j;f__;g__	0.1%	0.0%	0.1%	0.1%	0.0%	0.1%	0.1%
k__Bacteria;p__Proteobacteria;c__Deltaproteobacteria;o__NB1-j;f__JTB38;g__	0.0%	0.0%	0.0%	0.0%	0.0%	0.0%	0.0%
k__Bacteria;p__Proteobacteria;c__Deltaproteobacteria;o__NB1-j;f__MND4;g__	0.0%	0.0%	0.0%	0.0%	0.0%	0.0%	0.0%
k__Bacteria;p__Proteobacteria;c__Deltaproteobacteria;o__NB1-j;f__NB1-i;g__	0.1%	0.0%	0.0%	0.1%	0.0%	0.1%	0.1%
k__Bacteria;p__Proteobacteria;c__Deltaproteobacteria;o__NKB15;f__;g__	0.0%	0.0%	0.0%	0.0%	0.0%	0.0%	0.0%
k__Bacteria;p__Proteobacteria;c__Deltaproteobacteria;o__PB19;f__;g__	0.0%	0.0%	0.0%	0.0%	0.0%	0.0%	0.0%
k__Bacteria;p__Proteobacteria;c__Deltaproteobacteria;o__Spirobacillales;f__;g__	0.1%	0.1%	0.1%	0.1%	0.0%	0.0%	0.0%
k__Bacteria;p__Proteobacteria;c__Deltaproteobacteria;o__Sva0853;f__JTB36;g__	0.0%	0.0%	0.0%	0.0%	0.0%	0.0%	0.0%
k__Bacteria;p__Proteobacteria;c__Deltaproteobacteria;o__Syntrophobacterales;f__Syntrophaceae;g__	0.0%	0.0%	0.0%	0.0%	0.0%	0.0%	0.0%
k__Bacteria;p__Proteobacteria;c__Deltaproteobacteria;o__Syntrophobacterales;f__Syntrophaceae;g__Desulfobacca	0.0%	0.0%	0.0%	0.0%	0.0%	0.0%	0.0%
k__Bacteria;p__Proteobacteria;c__Deltaproteobacteria;o__Syntrophobacterales;f__Syntrophaceae;g__Smithella	0.0%	0.0%	0.0%	0.0%	0.0%	0.0%	0.0%
k__Bacteria;p__Proteobacteria;c__Deltaproteobacteria;o__Syntrophobacterales;f__Syntrophobacteraceae;Other	0.0%	0.0%	0.0%	0.0%	0.0%	0.0%	0.0%
k__Bacteria;p__Proteobacteria;c__Deltaproteobacteria;o__Syntrophobacterales;f__Syntrophobacteraceae;g__	1.5%	1.1%	1.0%	1.4%	1.4%	1.2%	2.9%
k__Bacteria;p__Proteobacteria;c__Deltaproteobacteria;o__Syntrophobacterales;f__Syntrophobacteraceae;g__Syntrophobacter	0.0%	0.0%	0.0%	0.0%	0.0%	0.0%	0.1%
k__Bacteria;p__Proteobacteria;c__Deltaproteobacteria;o__Syntrophobacterales;f__Syntrophorhabdaceae;g__	0.0%	0.0%	0.0%	0.0%	0.0%	0.0%	0.0%
k__Bacteria;p__Proteobacteria;c__Deltaproteobacteria;o__[Entotheonellales];f__;g__	0.0%	0.0%	0.0%	0.0%	0.0%	0.0%	0.0%
k__Bacteria;p__Proteobacteria;c__Deltaproteobacteria;o__[Entotheonellales];f__[Entotheonellaceae];g__	0.3%	0.1%	0.1%	0.3%	0.0%	0.1%	1.0%
k__Bacteria;p__Proteobacteria;c__Deltaproteobacteria;o__[Entotheonellales];f__[Entotheonellaceae];g__Candidatus Entotheonella	0.1%	0.4%	0.0%	0.0%	0.0%	0.0%	0.0%
k__Bacteria;p__Proteobacteria;c__Epsilonproteobacteria;o__Campylobacterales;f__Helicobacteraceae;g__Sulfuricurvum	0.0%	0.0%	0.0%	0.0%	0.0%	0.0%	0.0%
k__Bacteria;p__Proteobacteria;c__Gammaproteobacteria;Other;Other;Other	0.0%	0.0%	0.0%	0.0%	0.0%	0.0%	0.0%
k__Bacteria;p__Proteobacteria;c__Gammaproteobacteria;o__;f__;g__	0.1%	0.0%	0.0%	0.1%	0.3%	0.0%	0.0%
k__Bacteria;p__Proteobacteria;c__Gammaproteobacteria;o__34P16;f__;g__	0.0%	0.0%	0.0%	0.0%	0.0%	0.0%	0.0%
k__Bacteria;p__Proteobacteria;c__Gammaproteobacteria;o__Acidithiobacillales;f__;g__	0.0%	0.0%	0.0%	0.0%	0.0%	0.0%	0.0%
k__Bacteria;p__Proteobacteria;c__Gammaproteobacteria;o__Aeromonadales;f__Aeromonadaceae;g__	0.0%	0.0%	0.0%	0.0%	0.0%	0.0%	0.0%
k__Bacteria;p__Proteobacteria;c__Gammaproteobacteria;o__Alteromonadales;Other;Other	0.0%	0.1%	0.0%	0.1%	0.0%	0.0%	0.0%
k__Bacteria;p__Proteobacteria;c__Gammaproteobacteria;o__Alteromonadales;f__;g__	0.0%	0.0%	0.0%	0.0%	0.0%	0.0%	0.0%
k__Bacteria;p__Proteobacteria;c__Gammaproteobacteria;o__Alteromonadales;f__125ds10;g__	0.0%	0.0%	0.0%	0.0%	0.0%	0.0%	0.0%
k__Bacteria;p__Proteobacteria;c__Gammaproteobacteria;o__Alteromonadales;f__211ds20;g__	0.0%	0.2%	0.0%	0.0%	0.0%	0.0%	0.0%
k__Bacteria;p__Proteobacteria;c__Gammaproteobacteria;o__Alteromonadales;f__Alteromonadaceae;Other	0.0%	0.0%	0.0%	0.0%	0.0%	0.0%	0.0%
k__Bacteria;p__Proteobacteria;c__Gammaproteobacteria;o__Alteromonadales;f__Alteromonadaceae;g__	0.0%	0.0%	0.0%	0.0%	0.0%	0.0%	0.0%
k__Bacteria;p__Proteobacteria;c__Gammaproteobacteria;o__Alteromonadales;f__Alteromonadaceae;g__Cellvibrio	0.0%	0.0%	0.0%	0.0%	0.0%	0.0%	0.0%
k__Bacteria;p__Proteobacteria;c__Gammaproteobacteria;o__Alteromonadales;f__Alteromonadaceae;g__Marinobacter	0.0%	0.0%	0.0%	0.0%	0.0%	0.0%	0.0%
k__Bacteria;p__Proteobacteria;c__Gammaproteobacteria;o__Alteromonadales;f__HTCC2188;g__HTCC	0.0%	0.0%	0.0%	0.0%	0.0%	0.0%	0.0%
k__Bacteria;p__Proteobacteria;c__Gammaproteobacteria;o__Alteromonadales;f__OM60;g__	0.0%	0.1%	0.0%	0.2%	0.0%	0.0%	0.0%
k__Bacteria;p__Proteobacteria;c__Gammaproteobacteria;o__Alteromonadales;f__[Chromatiaceae];g__Rheinheimera	0.0%	0.0%	0.0%	0.0%	0.0%	0.0%	0.0%
k__Bacteria;p__Proteobacteria;c__Gammaproteobacteria;o__Chromatiales;Other;Other	0.0%	0.0%	0.0%	0.0%	0.0%	0.0%	0.0%
k__Bacteria;p__Proteobacteria;c__Gammaproteobacteria;o__Chromatiales;f__;g__	1.0%	0.1%	0.2%	0.0%	0.0%	5.7%	0.2%
k__Bacteria;p__Proteobacteria;c__Gammaproteobacteria;o__Chromatiales;f__Chromatiaceae;Other	0.0%	0.0%	0.0%	0.0%	0.0%	0.0%	0.0%
k__Bacteria;p__Proteobacteria;c__Gammaproteobacteria;o__Chromatiales;f__Chromatiaceae;g__	0.0%	0.0%	0.0%	0.0%	0.0%	0.0%	0.0%
k__Bacteria;p__Proteobacteria;c__Gammaproteobacteria;o__Chromatiales;f__Chromatiaceae;g__Nitrosococcus	0.0%	0.0%	0.0%	0.0%	0.0%	0.0%	0.0%
k__Bacteria;p__Proteobacteria;c__Gammaproteobacteria;o__Chromatiales;f__Ectothiorhodospiraceae;g__	0.0%	0.0%	0.0%	0.0%	0.0%	0.0%	0.0%
k__Bacteria;p__Proteobacteria;c__Gammaproteobacteria;o__Chromatiales;f__Ectothiorhodospiraceae;g__Halorhodospira	0.0%	0.0%	0.0%	0.0%	0.0%	0.0%	0.0%
k__Bacteria;p__Proteobacteria;c__Gammaproteobacteria;o__Chromatiales;f__Ectothiorhodospiraceae;g__Natronocella	0.0%	0.0%	0.0%	0.0%	0.0%	0.0%	0.0%
k__Bacteria;p__Proteobacteria;c__Gammaproteobacteria;o__Enterobacteriales;f__Enterobacteriaceae;Other	0.0%	0.0%	0.0%	0.0%	0.0%	0.0%	0.0%
k__Bacteria;p__Proteobacteria;c__Gammaproteobacteria;o__Enterobacteriales;f__Enterobacteriaceae;g__	0.0%	0.0%	0.0%	0.0%	0.0%	0.0%	0.0%
k__Bacteria;p__Proteobacteria;c__Gammaproteobacteria;o__Enterobacteriales;f__Enterobacteriaceae;g__Providencia	0.0%	0.0%	0.0%	0.0%	0.0%	0.0%	0.0%
k__Bacteria;p__Proteobacteria;c__Gammaproteobacteria;o__HOC36;f__;g__	0.0%	0.0%	0.0%	0.0%	0.0%	0.0%	0.1%
k__Bacteria;p__Proteobacteria;c__Gammaproteobacteria;o__HTCC2188;f__HTCC2089;g__	0.1%	0.1%	0.0%	0.3%	0.0%	0.0%	0.1%

	k__Bacteria;p__Proteobacteria;c__Gammaproteobacteria;o__Legionellales;Other;Other	0.0%	0.0%	0.0%	0.0%	0.0%	0.0%	0.0%
	k__Bacteria;p__Proteobacteria;c__Gammaproteobacteria;o__Legionellales;f__;g__	0.1%	0.0%	0.1%	0.1%	0.1%	0.0%	0.0%
	k__Bacteria;p__Proteobacteria;c__Gammaproteobacteria;o__Legionellales;f__Coxiellaceae;Other	0.0%	0.0%	0.0%	0.0%	0.0%	0.0%	0.0%
	k__Bacteria;p__Proteobacteria;c__Gammaproteobacteria;o__Legionellales;f__Coxiellaceae;g__	0.2%	0.2%	0.1%	0.3%	0.4%	0.2%	0.1%
	k__Bacteria;p__Proteobacteria;c__Gammaproteobacteria;o__Legionellales;f__Coxiellaceae;g__Aquicella	0.1%	0.0%	0.0%	0.1%	0.5%	0.0%	0.0%
	k__Bacteria;p__Proteobacteria;c__Gammaproteobacteria;o__Legionellales;f__Coxiellaceae;g__Coxiella	0.0%	0.0%	0.0%	0.0%	0.0%	0.0%	0.0%
	k__Bacteria;p__Proteobacteria;c__Gammaproteobacteria;o__Legionellales;f__Coxiellaceae;g__Rickettsiella	0.0%	0.0%	0.0%	0.0%	0.0%	0.0%	0.0%
	k__Bacteria;p__Proteobacteria;c__Gammaproteobacteria;o__Legionellales;f__Legionellaceae;Other	0.0%	0.0%	0.0%	0.0%	0.0%	0.0%	0.0%
	k__Bacteria;p__Proteobacteria;c__Gammaproteobacteria;o__Legionellales;f__Legionellaceae;g__	0.0%	0.1%	0.0%	0.1%	0.0%	0.0%	0.0%
	k__Bacteria;p__Proteobacteria;c__Gammaproteobacteria;o__Legionellales;f__Legionellaceae;g__Legionella	0.0%	0.0%	0.0%	0.1%	0.0%	0.0%	0.0%
	k__Bacteria;p__Proteobacteria;c__Gammaproteobacteria;o__Legionellales;f__Legionellaceae;g__Tatlockia	0.0%	0.0%	0.0%	0.0%	0.0%	0.0%	0.0%
	k__Bacteria;p__Proteobacteria;c__Gammaproteobacteria;o__Methylococcales;f__Crenotrichaceae;g__Crenothrix	0.0%	0.0%	0.0%	0.0%	0.0%	0.0%	0.0%
	k__Bacteria;p__Proteobacteria;c__Gammaproteobacteria;o__Methylococcales;f__Methylococcaceae;Other	0.0%	0.0%	0.0%	0.0%	0.0%	0.0%	0.1%
	k__Bacteria;p__Proteobacteria;c__Gammaproteobacteria;o__Methylococcales;f__Methylococcaceae;g__	0.0%	0.0%	0.0%	0.0%	0.0%	0.0%	0.1%
	k__Bacteria;p__Proteobacteria;c__Gammaproteobacteria;o__Methylococcales;f__Methylococcaceae;g__Methylocaldum	0.0%	0.0%	0.0%	0.0%	0.0%	0.0%	0.0%
	k__Bacteria;p__Proteobacteria;c__Gammaproteobacteria;o__Methylococcales;f__Methylococcaceae;g__Methylomonas	0.0%	0.0%	0.0%	0.0%	0.0%	0.0%	0.0%
	k__Bacteria;p__Proteobacteria;c__Gammaproteobacteria;o__Methylococcales;f__Methylococcaceae;g__Methylosarcina	0.0%	0.0%	0.0%	0.0%	0.0%	0.0%	0.0%
	k__Bacteria;p__Proteobacteria;c__Gammaproteobacteria;o__Oceanospirillales;f__Halomonadaceae;g__	0.0%	0.0%	0.0%	0.0%	0.0%	0.0%	0.0%
	k__Bacteria;p__Proteobacteria;c__Gammaproteobacteria;o__Oceanospirillales;f__Halomonadaceae;g__Halomonas	0.0%	0.0%	0.0%	0.0%	0.0%	0.0%	0.0%
	k__Bacteria;p__Proteobacteria;c__Gammaproteobacteria;o__Oceanospirillales;f__Oceanospirillaceae;g__	0.0%	0.0%	0.0%	0.0%	0.0%	0.0%	0.0%
	k__Bacteria;p__Proteobacteria;c__Gammaproteobacteria;o__Oceanospirillales;f__Oleiphilaceae;g__	0.0%	0.0%	0.0%	0.0%	0.0%	0.0%	0.0%
	k__Bacteria;p__Proteobacteria;c__Gammaproteobacteria;o__PYR10d3;f__;g__	0.0%	0.0%	0.0%	0.0%	0.0%	0.0%	0.0%
	k__Bacteria;p__Proteobacteria;c__Gammaproteobacteria;o__Pseudomonadales;Other;Other	0.0%	0.0%	0.0%	0.0%	0.0%	0.0%	0.0%
	k__Bacteria;p__Proteobacteria;c__Gammaproteobacteria;o__Pseudomonadales;f__Moraxellaceae;Other	0.0%	0.0%	0.0%	0.0%	0.0%	0.0%	0.0%
	k__Bacteria;p__Proteobacteria;c__Gammaproteobacteria;o__Pseudomonadales;f__Moraxellaceae;g__	0.1%	0.0%	0.0%	0.0%	0.6%	0.0%	0.0%
	k__Bacteria;p__Proteobacteria;c__Gammaproteobacteria;o__Pseudomonadales;f__Moraxellaceae;g__Acinetobacter	0.0%	0.0%	0.0%	0.0%	0.0%	0.0%	0.0%
	k__Bacteria;p__Proteobacteria;c__Gammaproteobacteria;o__Pseudomonadales;f__Moraxellaceae;g__Perlucidibaca	0.0%	0.0%	0.1%	0.0%	0.0%	0.0%	0.0%
	k__Bacteria;p__Proteobacteria;c__Gammaproteobacteria;o__Pseudomonadales;f__Pseudomonadaceae;Other	0.0%	0.0%	0.0%	0.0%	0.0%	0.0%	0.0%
	k__Bacteria;p__Proteobacteria;c__Gammaproteobacteria;o__Pseudomonadales;f__Pseudomonadaceae;g__	0.0%	0.0%	0.0%	0.0%	0.0%	0.0%	0.0%
	k__Bacteria;p__Proteobacteria;c__Gammaproteobacteria;o__Pseudomonadales;f__Pseudomonadaceae;g__Pseudomonas	0.1%	0.2%	0.1%	0.2%	0.3%	0.1%	0.0%
	k__Bacteria;p__Proteobacteria;c__Gammaproteobacteria;o__Thiotrichales;f__Piscirickettsiaceae;g__	0.6%	2.3%	0.2%	0.6%	0.0%	0.1%	0.4%
	k__Bacteria;p__Proteobacteria;c__Gammaproteobacteria;o__Xanthomonadales;f__Sinobacteraceae;g__	1.2%	1.6%	0.6%	1.5%	1.4%	1.1%	0.8%
	k__Bacteria;p__Proteobacteria;c__Gammaproteobacteria;o__Xanthomonadales;f__Sinobacteraceae;g__Nevskia	0.0%	0.0%	0.0%	0.0%	0.0%	0.0%	0.0%
	k__Bacteria;p__Proteobacteria;c__Gammaproteobacteria;o__Xanthomonadales;f__Sinobacteraceae;g__Steroidobacter	0.3%	0.8%	0.4%	0.3%	0.0%	0.1%	0.4%
	k__Bacteria;p__Proteobacteria;c__Gammaproteobacteria;o__Xanthomonadales;f__Xanthomonadaceae;Other	0.1%	0.1%	0.6%	0.0%	0.0%	0.0%	0.0%
	k__Bacteria;p__Proteobacteria;c__Gammaproteobacteria;o__Xanthomonadales;f__Xanthomonadaceae;g__	0.3%	0.8%	0.5%	0.5%	0.2%	0.1%	0.0%
	k__Bacteria;p__Proteobacteria;c__Gammaproteobacteria;o__Xanthomonadales;f__Xanthomonadaceae;g__Arenimonas	0.0%	0.1%	0.0%	0.0%	0.0%	0.0%	0.0%
	k__Bacteria;p__Proteobacteria;c__Gammaproteobacteria;o__Xanthomonadales;f__Xanthomonadaceae;g__Dokdonella	0.0%	0.0%	0.0%	0.0%	0.0%	0.0%	0.0%
	k__Bacteria;p__Proteobacteria;c__Gammaproteobacteria;o__Xanthomonadales;f__Xanthomonadaceae;g__Luteibacter	0.0%	0.0%	0.0%	0.0%	0.0%	0.0%	0.0%
	k__Bacteria;p__Proteobacteria;c__Gammaproteobacteria;o__Xanthomonadales;f__Xanthomonadaceae;g__Luteimonas	0.0%	0.0%	0.1%	0.0%	0.0%	0.0%	0.0%
	k__Bacteria;p__Proteobacteria;c__Gammaproteobacteria;o__Xanthomonadales;f__Xanthomonadaceae;g__Lysobacter	0.1%	0.1%	0.3%	0.0%	0.0%	0.0%	0.0%
	k__Bacteria;p__Proteobacteria;c__Gammaproteobacteria;o__Xanthomonadales;f__Xanthomonadaceae;g__Pseudoxanthomonas	0.0%	0.0%	0.0%	0.0%	0.0%	0.0%	0.0%
	k__Bacteria;p__Proteobacteria;c__Gammaproteobacteria;o__Xanthomonadales;f__Xanthomonadaceae;g__Rhodanobacter	0.1%	0.0%	0.0%	0.1%	0.5%	0.0%	0.0%
	k__Bacteria;p__Proteobacteria;c__Gammaproteobacteria;o__Xanthomonadales;f__Xanthomonadaceae;g__Stenotrophomonas	0.0%	0.0%	0.0%	0.0%	0.0%	0.0%	0.0%
	k__Bacteria;p__Proteobacteria;c__Gammaproteobacteria;o__Xanthomonadales;f__Xanthomonadaceae;g__Thermomonas	0.0%	0.0%	0.0%	0.0%	0.0%	0.0%	0.0%
	k__Bacteria;p__Proteobacteria;c__Gammaproteobacteria;o__[Marinicellales];f__[Marinicellaceae];g__	0.0%	0.0%	0.0%	0.0%	0.0%	0.0%	0.0%
	k__Bacteria;p__Proteobacteria;c__TA18;o__CV90;f__;g__	0.0%	0.0%	0.0%	0.0%	0.0%	0.0%	0.0%
	k__Bacteria;p__Proteobacteria;c__TA18;o__PHOS-HD29;f__;g__	0.0%	0.0%	0.0%	0.1%	0.0%	0.0%	0.0%
	k__Bacteria;p__SBR1093;c__;o__;f__;g__	0.0%	0.0%	0.0%	0.0%	0.0%	0.2%	0.0%
	k__Bacteria;p__SBR1093;c__VHS-B5-50;o__;f__;g__	0.0%	0.0%	0.0%	0.0%	0.0%	0.0%	0.0%
	k__Bacteria;p__SC4;c__;o__;f__;g__	0.0%	0.0%	0.0%	0.0%	0.0%	0.0%	0.0%
	k__Bacteria;p__SR1;c__;o__;f__;g__	0.0%	0.0%	0.0%	0.0%	0.0%	0.0%	0.0%
	k__Bacteria;p__Spirochaetes;c__GN05;o__;f__;g__	0.0%	0.0%	0.0%	0.0%	0.0%	0.0%	0.0%
	k__Bacteria;p__Spirochaetes;c__[Leptospirae];o__[Leptospirales];f__Leptospiraceae;g__Leptonema	0.0%	0.0%	0.0%	0.0%	0.0%	0.0%	0.0%
	k__Bacteria;p__Spirochaetes;c__[Leptospirae];o__[Leptospirales];f__Leptospiraceae;g__Leptospira	0.0%	0.0%	0.0%	0.0%	0.0%	0.0%	0.0%
	k__Bacteria;p__Spirochaetes;c__[Leptospirae];o__[Leptospirales];f__Leptospiraceae;g__Turneriella	0.0%	0.0%	0.0%	0.0%	0.0%	0.0%	0.0%
	k__Bacteria;p__TM6;c__SBRH58;o__;f__;g__	0.0%	0.0%	0.0%	0.0%	0.0%	0.0%	0.0%
	k__Bacteria;p__TM6;c__SJA-4;Other;Other;Other	0.0%	0.0%	0.0%	0.0%	0.0%	0.0%	0.0%
	k__Bacteria;p__TM6;c__SJA-4;o__;f__;g__	0.1%	0.2%	0.1%	0.1%	0.1%	0.1%	0.2%
	k__Bacteria;p__TM6;c__SJA-4;o__S1198;f__;g__	0.0%	0.0%	0.0%	0.0%	0.0%	0.0%	0.0%
	k__Bacteria;p__TM6;c__SJA-4;o__YJF2-48;f__;g__	0.0%	0.0%	0.0%	0.0%	0.0%	0.0%	0.0%
	k__Bacteria;p__TM7;Other;Other;Other;Other	0.0%	0.0%	0.0%	0.0%	0.0%	0.0%	0.0%
	k__Bacteria;p__TM7;c__;o__;f__;g__	0.0%	0.0%	0.0%	0.0%	0.0%	0.0%	0.0%
	k__Bacteria;p__TM7;c__MJK10;o__;f__;g__	0.0%	0.0%	0.0%	0.0%	0.0%	0.0%	0.0%
	k__Bacteria;p__TM7;c__SC3;o__;f__;g__	0.0%	0.0%	0.0%	0.0%	0.0%	0.0%	0.0%
	k__Bacteria;p__TM7;c__TM7-1;o__;f__;g__	0.4%	0.5%	0.4%	0.5%	0.1%	0.3%	0.4%
	k__Bacteria;p__TM7;c__TM7-3;Other;Other;Other	0.0%	0.0%	0.0%	0.0%	0.1%	0.0%	0.0%

	k__Bacteria;p__TM7;c__TM7-3;o__;f__;g__	0.1%	0.1%	0.2%	0.0%	0.0%	0.0%	0.0%
	k__Bacteria;p__TM7;c__TM7-3;o__Blgi18;f__;g__	0.0%	0.0%	0.0%	0.0%	0.0%	0.0%	0.0%
	k__Bacteria;p__TM7;c__TM7-3;o__CW040;f__F16;g__	0.0%	0.0%	0.0%	0.0%	0.0%	0.0%	0.0%
	k__Bacteria;p__TM7;c__TM7-3;o__EW055;f__;g__	0.0%	0.0%	0.0%	0.0%	0.0%	0.0%	0.0%
	k__Bacteria;p__TM7;c__TM7-3;o__I025;f__;g__	0.0%	0.0%	0.0%	0.0%	0.0%	0.0%	0.0%
	k__Bacteria;p__Tenericutes;c__Mollicutes;o__;f__;g__	0.0%	0.0%	0.0%	0.0%	0.0%	0.0%	0.0%
	k__Bacteria;p__Tenericutes;c__Mollicutes;o__Anaeroplasmatales;f__Anaeroplasmataceae;g__	0.0%	0.0%	0.0%	0.0%	0.0%	0.0%	0.0%
	k__Bacteria;p__Tenericutes;c__Mollicutes;o__Anaeroplasmatales;f__Anaeroplasmataceae;g__Asteroleplasma	0.0%	0.0%	0.0%	0.0%	0.0%	0.0%	0.0%
	k__Bacteria;p__Verrucomicrobia;c__;o__;f__;g__	0.0%	0.0%	0.0%	0.0%	0.0%	0.0%	0.0%
	k__Bacteria;p__Verrucomicrobia;c__Opitutae;o__;f__;g__	0.0%	0.1%	0.0%	0.0%	0.0%	0.0%	0.0%
	k__Bacteria;p__Verrucomicrobia;c__Opitutae;o__Opitutales;f__Opitutaceae;g__	0.0%	0.1%	0.0%	0.1%	0.0%	0.0%	0.0%
	k__Bacteria;p__Verrucomicrobia;c__Opitutae;o__Opitutales;f__Opitutaceae;g__Opitutus	0.1%	0.2%	0.1%	0.1%	0.0%	0.0%	0.1%
	k__Bacteria;p__Verrucomicrobia;c__Opitutae;o__[Cerasicoccales];f__[Cerasicoccaceae];g__	0.0%	0.0%	0.0%	0.0%	0.0%	0.0%	0.0%
	k__Bacteria;p__Verrucomicrobia;c__Verruco-5;o__LD1-PB3;f__;g__	0.0%	0.0%	0.0%	0.0%	0.0%	0.0%	0.0%
	k__Bacteria;p__Verrucomicrobia;c__Verruco-5;o__WCHB1-41;f__;g__	0.0%	0.0%	0.0%	0.0%	0.0%	0.0%	0.0%
	k__Bacteria;p__Verrucomicrobia;c__Verrucomicrobiae;o__Verrucomicrobiales;f__Verrucomicrobiaceae;g__	0.1%	0.1%	0.0%	0.2%	0.0%	0.0%	0.1%
	k__Bacteria;p__Verrucomicrobia;c__Verrucomicrobiae;o__Verrucomicrobiales;f__Verrucomicrobiaceae;g__Luteolibacter	0.0%	0.1%	0.0%	0.1%	0.0%	0.0%	0.0%
	k__Bacteria;p__Verrucomicrobia;c__Verrucomicrobiae;o__Verrucomicrobiales;f__Verrucomicrobiaceae;g__Prosthecobacter	0.0%	0.0%	0.0%	0.0%	0.0%	0.0%	0.0%
	k__Bacteria;p__Verrucomicrobia;c__Verrucomicrobiae;o__Verrucomicrobiales;f__Verrucomicrobiaceae;g__Verrucomicrobium	0.0%	0.0%	0.0%	0.0%	0.0%	0.0%	0.0%
	k__Bacteria;p__Verrucomicrobia;c__[Methylacidiphilae];o__Methylacidiphilales;f__;g__	0.0%	0.0%	0.0%	0.0%	0.0%	0.0%	0.0%
	k__Bacteria;p__Verrucomicrobia;c__[Methylacidiphilae];o__Methylacidiphilales;f__LD19;g__	0.0%	0.0%	0.0%	0.0%	0.0%	0.0%	0.0%
	k__Bacteria;p__Verrucomicrobia;c__[Methylacidiphilae];o__S-BQ2-57;f__;g__	0.0%	0.0%	0.0%	0.1%	0.0%	0.0%	0.0%
	k__Bacteria;p__Verrucomicrobia;c__[Pedosphaerae];o__[Pedosphaerales];Other;Other	0.0%	0.0%	0.0%	0.0%	0.0%	0.0%	0.0%
	k__Bacteria;p__Verrucomicrobia;c__[Pedosphaerae];o__[Pedosphaerales];f__;g__	0.3%	0.2%	0.1%	0.5%	0.0%	0.1%	0.6%
	k__Bacteria;p__Verrucomicrobia;c__[Pedosphaerae];o__[Pedosphaerales];f__Ellin515;g__	0.1%	0.0%	0.0%	0.2%	0.0%	0.0%	0.4%
	k__Bacteria;p__Verrucomicrobia;c__[Pedosphaerae];o__[Pedosphaerales];f__Ellin517;g__	0.1%	0.2%	0.4%	0.1%	0.0%	0.0%	0.1%
	k__Bacteria;p__Verrucomicrobia;c__[Pedosphaerae];o__[Pedosphaerales];f__OPB35;g__	0.1%	0.0%	0.2%	0.0%	0.0%	0.1%	0.1%
	k__Bacteria;p__Verrucomicrobia;c__[Pedosphaerae];o__[Pedosphaerales];f__R4-41B;g__	0.0%	0.0%	0.0%	0.0%	0.0%	0.0%	0.0%
	k__Bacteria;p__Verrucomicrobia;c__[Pedosphaerae];o__[Pedosphaerales];f__[Pedosphaeraceae];Other	0.0%	0.0%	0.0%	0.0%	0.0%	0.0%	0.0%
	k__Bacteria;p__Verrucomicrobia;c__[Pedosphaerae];o__[Pedosphaerales];f__[Pedosphaeraceae];g__	0.0%	0.0%	0.0%	0.0%	0.0%	0.0%	0.0%
	k__Bacteria;p__Verrucomicrobia;c__[Pedosphaerae];o__[Pedosphaerales];f__[Pedosphaeraceae];g__Pedosphaera	0.0%	0.0%	0.0%	0.0%	0.0%	0.0%	0.0%
	k__Bacteria;p__Verrucomicrobia;c__[Pedosphaerae];o__[Pedosphaerales];f__auto67_4W;g__	0.1%	0.1%	0.0%	0.3%	0.0%	0.0%	0.1%
	k__Bacteria;p__Verrucomicrobia;c__[Spartobacteria];o__[Chthoniobacterales];f__01D2Z36;g__	0.0%	0.0%	0.0%	0.0%	0.0%	0.0%	0.0%
	k__Bacteria;p__Verrucomicrobia;c__[Spartobacteria];o__[Chthoniobacterales];f__[Chthoniobacteraceae];Other	0.0%	0.0%	0.0%	0.0%	0.0%	0.0%	0.0%
	k__Bacteria;p__Verrucomicrobia;c__[Spartobacteria];o__[Chthoniobacterales];f__[Chthoniobacteraceae];g__	0.1%	0.1%	0.1%	0.2%	0.0%	0.0%	0.2%
	k__Bacteria;p__Verrucomicrobia;c__[Spartobacteria];o__[Chthoniobacterales];f__[Chthoniobacteraceae];g__Candidatus Xiphinematobacter	0.4%	0.1%	0.0%	0.3%	0.5%	0.2%	1.1%
	k__Bacteria;p__Verrucomicrobia;c__[Spartobacteria];o__[Chthoniobacterales];f__[Chthoniobacteraceae];g__Chthoniobacter	0.0%	0.0%	0.0%	0.1%	0.0%	0.0%	0.0%
	k__Bacteria;p__Verrucomicrobia;c__[Spartobacteria];o__[Chthoniobacterales];f__[Chthoniobacteraceae];g__DA101	0.4%	0.0%	0.0%	1.0%	0.1%	0.0%	1.0%
	k__Bacteria;p__Verrucomicrobia;c__[Spartobacteria];o__[Chthoniobacterales];f__[Chthoniobacteraceae];g__Ellin506	0.0%	0.0%	0.0%	0.0%	0.0%	0.0%	0.0%
	k__Bacteria;p__Verrucomicrobia;c__[Spartobacteria];o__[Chthoniobacterales];f__[Chthoniobacteraceae];g__OR-59	0.0%	0.0%	0.0%	0.0%	0.0%	0.0%	0.0%
	k__Bacteria;p__Verrucomicrobia;c__[Spartobacteria];o__[Chthoniobacterales];f__[Chthoniobacteraceae];g__heteroC45_4W	0.0%	0.0%	0.0%	0.0%	0.0%	0.0%	0.0%
	k__Bacteria;p__WPS-2;c__;o__;f__;g__	0.3%	0.0%	0.0%	0.0%	1.6%	0.0%	0.0%
	k__Bacteria;p__WS2;c__Kazan-3B-09;o__;f__;g__	0.0%	0.0%	0.0%	0.0%	0.0%	0.0%	0.0%
	k__Bacteria;p__WS2;c__SHA-109;o__;f__;g__	0.0%	0.0%	0.0%	0.0%	0.0%	0.0%	0.0%
	k__Bacteria;p__WS3;c__PRR-12;o__;f__;g__	0.0%	0.0%	0.0%	0.0%	0.0%	0.0%	0.0%
	k__Bacteria;p__WS3;c__PRR-12;o__LD1-PA13;f__;g__	0.0%	0.0%	0.0%	0.0%	0.0%	0.0%	0.1%
	k__Bacteria;p__WS3;c__PRR-12;o__PBS-III-9;f__;g__	0.0%	0.0%	0.0%	0.0%	0.0%	0.0%	0.0%
	k__Bacteria;p__WS3;c__PRR-12;o__Sediment-1;Other;Other	0.0%	0.0%	0.0%	0.0%	0.0%	0.0%	0.0%
	k__Bacteria;p__WS3;c__PRR-12;o__Sediment-1;f__;g__	0.1%	0.0%	0.2%	0.1%	0.0%	0.3%	0.2%
	k__Bacteria;p__WS3;c__PRR-12;o__Sediment-1;f__CV106;g__	0.0%	0.0%	0.0%	0.0%	0.0%	0.0%	0.0%
	k__Bacteria;p__WS3;c__PRR-12;o__Sediment-1;f__PRR-10;g__	0.1%	0.0%	0.2%	0.2%	0.0%	0.4%	0.2%
	k__Bacteria;p__WS3;c__PRR-12;o__wb1_H11;f__;g__	0.0%	0.0%	0.0%	0.0%	0.0%	0.0%	0.0%
	k__Bacteria;p__WS4;c__;o__;f__;g__	0.0%	0.0%	0.0%	0.0%	0.0%	0.0%	0.0%
	k__Bacteria;p__WS5;c__;o__;f__;g__	0.0%	0.0%	0.0%	0.0%	0.0%	0.0%	0.0%
	k__Bacteria;p__WS6;c__;o__;f__;g__	0.0%	0.0%	0.0%	0.0%	0.0%	0.0%	0.0%
	k__Bacteria;p__WS6;c__B142;o__;f__;g__	0.0%	0.0%	0.0%	0.0%	0.0%	0.0%	0.0%
	k__Bacteria;p__WS6;c__SC72;o__;f__;g__	0.0%	0.0%	0.0%	0.0%	0.0%	0.0%	0.0%
	k__Bacteria;p__ZB3;c__;o__;f__;g__	0.0%	0.0%	0.0%	0.0%	0.0%	0.0%	0.0%
	k__Bacteria;p__ZB3;c__BS119;o__;f__;g__	0.0%	0.0%	0.0%	0.0%	0.0%	0.0%	0.0%
	k__Bacteria;p__[Caldithrix];c__KSB1;o__Ucn15732;f__;g__	0.0%	0.0%	0.0%	0.0%	0.0%	0.0%	0.0%
	k__Bacteria;p__[Thermi];c__Deinococci;o__Deinococcales;f__Deinococcaceae;g__Deinococcus	0.0%	0.0%	0.0%	0.0%	0.0%	0.0%	0.0%
	k__Bacteria;p__[Thermi];c__Deinococci;o__Deinococcales;f__Deinococcaceae;g__R18-435	0.0%	0.0%	0.0%	0.0%	0.0%	0.0%	0.0%
	k__Bacteria;p__[Thermi];c__Deinococci;o__Deinococcales;f__Trueperaceae;g__	0.0%	0.0%	0.0%	0.0%	0.0%	0.0%	0.0%
	k__Bacteria;p__[Thermi];c__Deinococci;o__Deinococcales;f__Trueperaceae;g__B-42	0.0%	0.0%	0.0%	0.0%	0.0%	0.0%	0.0%
	k__Bacteria;p__[Thermi];c__Deinococci;o__Deinococcales;f__Trueperaceae;g__Truepera	0.0%	0.0%	0.0%	0.0%	0.0%	0.0%	0.0%
